# Are Mediterranean Societies “Cultures of Honor?”: Prevalence and Implications of a Cultural Logic of Honor Across Three World Regions

**DOI:** 10.1177/01461672241295500

**Published:** 2024-12-30

**Authors:** Vivian L. Vignoles, Alexander Kirchner-Häusler, Ayse K. Uskul, Susan E. Cross, Rosa Rodriguez-Bailón, Isabella R. L. Bossom, Vanessa A. Castillo, Meral Gezici-Yalçın, Charles Harb, Keiko Ishii, Panagiota Karamaouna, Konstantinos Kafetsios, Evangelia Kateri, Juan Matamoros-Lima, Rania Miniesy, Jinkyung Na, Zafer Özkan, Stefano Pagliaro, Charis Psaltis, Dina Rabie, Manuel Teresi, Yukiko Uchida, Michael J. A. Wohl

**Affiliations:** 1 University of Sussex, Brighton, UK; 2 University of Kent, UK; 3 Iowa State University, Ames, USA; 4 University of Granada, Spain; 5 Carleton University, Ottawa, Ontario, Canada; 6 Coe College, Cedar Rapids, IA, USA; 7 Bielefeld University, Germany; 8 Doha Institute for Graduate Studies, Qatar; 9 American University of Beirut, Lebanon; 10 Nagoya University, Japan; 11 University of Crete, Rethimno, Greece; 12 Aristotle University of Thessaloniki, Greece; 13 The British University in Egypt, Cairo, Egypt; 14 Sogang University, Seoul, South Korea; 15 Ordu University, Türkiye; 16 University di Chieti-Pescara, Italy; 17 University of Cyprus, Cyprus; 18 Kyoto University, Japan

**Keywords:** Mediterranean region, honor, face, dignity, cognitive style, social orientation

## Abstract

Mediterranean societies are often labeled as “honor cultures,” in contrast with presumed “dignity” and “face” cultures of Anglo-Western and East Asian societies. We measured these cultural logics in two large-scale surveys (Studies 1 & 3: *N* = 2,942 students from 11 societies; Study 2: *N* = 5,471 adults from 14 societies). Middle Eastern and North African groups perceived honor values as the most normative in their societies, followed by Southeast European, and then Latin-European groups (who were comparable to Anglo-Western and East-Asian groups). East-Asian and Anglo-Western groups, respectively, perceived face and dignity values as most normative. Culture-level variation in perceived normative honor values, but not personal values, accounted for previously reported differences between Mediterranean and non-Mediterranean samples in several (but not all) measures of social cognitive tendencies. We conclude that a cultural logic of honor plays a role in Mediterranean societies, but labeling these societies as “honor cultures” is oversimplistic.

Social scientists increasingly link the cultural construct of “honor” to core questions in their fields. Researchers across numerous disciplines have considered the role of honor in behaviors ranging from health-care seeking to warfare (Aslani et al., 2016; Brooks et al., 2018; Dafoe & Caughey, 2016; Fosse et al., 2017; Foster et al., 2021; Gerodimos, 2022; Grosjean, 2014; Korteweg & Yurdakul, 2009; Messner et al., 2005; Pely, 2011; Rodriguez Mosquera, 2016; Tschantret, 2020; Wikan, 2008). This surging interest heightens the need for a comprehensive conceptual and empirical investigation of the construct of “honor,” its variation across cultural groups and societies, and its potential for explaining how culture shapes psychological tendencies and characteristics.

The current research provides a major step forward by mapping culture-level variation in the perceived normativity and personal endorsement of honor values and honor-related concerns across cultural groups from three global regions and assessing their potential to explain cultural variations in social orientation and cognitive style. We evaluate the prevalence and role of honor in diverse societies around the Mediterranean basin—often labeled as “honor cultures”—compared to more commonly studied societies in East Asian and Anglo-Western world regions. Our work provides novel insights into the structure, measurement, and predictive utility of honor as a distinct, but multifaceted, cultural construct.

## Cultural Logics of Honor, Face, and Dignity

According to cultural psychologists, societies have different *cultural logics* (Leung & Cohen, 2011)—constellations of shared beliefs, values, and practices that cohere around a central theme, affording distinct approaches to questions about social order (e.g., cooperation) and valuation (e.g., sources of self-worth), and shaping individuals’ orientations to core social psychological issues such as morality, punishment, and reciprocity. Literature currently adopts a tripartite distinction between cultural logics of *honor*, *face*, and *dignity*. Cultural psychology research to date often compares Anglo-Western and East Asian cultural contexts, thought to promote cultural logics of dignity and face, respectively, whereas societies that are thought to promote a cultural logic of honor have been studied much less often.

In societies thought to promote a cultural logic of *dignity* (e.g., North American and Northwest European societies), individuals are presumed to have inherent, equal, and inalienable worth that is not easily lost through one’s own or others’ actions (Stewart, 1994). Accordingly, people living in these societies expect to behave according to their own internal standards, beliefs, and values, rather than external influences such as social condemnation or punishment (Ayers, 1984), and they expect legal systems to enforce lawful behavior, provide protection, and administer justice fairly and equally to all (A. Smith, 1976).

In societies thought to promote a cultural logic of *face* (e.g., East Asian societies), individuals’ self-worth and social status are believed to be largely afforded by others, based on adhering to social and relational obligations and fulfilling the expectations associated with one’s societal roles (Heine, 2001; Ho, 1976). Face is easily lost if norms are violated or expectations are unmet (Hamamura et al., 2009). Hence, individuals adopt actions (e.g., cooperation, self-restraint, conformity) to protect their own and close others’ face, and concerns for humility, ingroup harmony, and hierarchy are widespread (Kim & Cohen, 2010).

Recently, models describing a cultural logic of *honor* have received increasing attention in comparative research, often focusing on cultural settings in Southern Europe, Middle East and North Africa (MENA), Latin America, or parts of the southern USA (for reviews, see Uskul et al., 2019; Uskul, Cross, & Günsoy, 2023). A cultural logic of honor is thought to characterize societies where livelihoods historically depended on portable resources (e.g., herds of cattle) and where the rule of law is lacking (Nisbett & Cohen, 1996). Members of these societies develop strategies to express and gain a reputation for toughness, strength, and morality, while deriving social capital and support from their interdependence with close-knit family groups. Honor is thus shaped by one’s self-view but also by one’s social reputation and the societal standards against which one’s behavior is assessed (Cross et al., 2014; Gilmore, 1987; Peristiany, 1966). Honor is highly relational; individuals’ actions have direct implications for the honor of close others and vice versa (Araji, 2000; Korteweg & Yurdakul, 2009; Rodriguez Mosquera et al., 2008; Uskul et al., 2012). Social expectations for how to maintain and defend one’s honor are highly gendered; societies often emphasize different norms related to claiming and maintaining honor for men and women (e.g., strength for men, purity for women; Rodriguez Mosquera, 2016).

## Goals of the Current Research

Research comparing inhabitants of societies where dignity and face logics are thought to prevail has revealed numerous differences in psychological functioning (Markus & Kitayama, 2010). Research into contexts where honor logic is thought to prevail has emerged more recently, and existing literature suffers from several limitations, which we seek to address.

### Mapping Regional Variation in Honor, Face, and Dignity Logics

Crucially, existing literature has lacked a systematic investigation into whether people from the theoretically expected societal contexts (a) perceive their societies to emphasize honor, face, and dignity logics to different degrees and (b) personally endorse the corresponding values and concerns differently. These crucial theoretical assumptions seemingly underlie the common approach of comparing cultural samples from locations thought to represent “honor,” “face,” and “dignity” cultures but have not been tested comprehensively (for an initial attempt, see P. B. Smith et al., 2021). Here, we sought to map the distribution of culture-level variation in the perceived and actual prevalence of honor, face, and dignity values and concerns across societies spanning three world regions that are commonly labeled as prototypical “honor,” “face,” and “dignity” cultures: societies around the Mediterranean basin, societies in East Asia, and Anglo-Western societies.

We focused on Mediterranean societies for several reasons. Although anthropological work has repeatedly asserted the importance of honor for social processes in Mediterranean societies (Gregg, 2005; Peristiany, 1966; Pitt-Rivers, 1966), this region has been relatively neglected in cultural psychology. Thus, we respond to growing demands to make psychology a global science by studying a wider range of cultural contexts (Ghai, 2021; Krys et al., 2022; Syed & Kathawalla, 2022). Notably, this region hosts groups from different ethnic, religious, and cultural backgrounds that live under different political systems and economic conditions, allowing us to examine whether honor is a defining characteristic of Mediterranean cultures despite considerable diversity in other respects. Moreover, diverse Mediterranean societies are sometimes thought to embody different forms of honor (Gilmore, 1987; Horden & Purcell, 2000), allowing us to compare across potentially different honor systems.^1^

Research has largely neglected how honor logics might differ across societies that have been collectively labeled as “cultures of honor” (for a partial exception, see Guerra et al., 2013). This labeling implies that dynamics of honor are comparable across sometimes highly different societies, which may lead to a false expectation that findings from one cultural group speak for all. For example, researchers have used cultural contexts in Latin Europe (e.g., Spain: Rodriguez Mosquera et al., 2002b) and the Middle East (e.g.: Türkiye: Uskul & Cross, 2019) as exemplars of “honor cultures” to compare against Northwest European (e.g., the Netherlands) or North American (e.g., northern US) “non-honor” contexts. Such comparisons have yielded highly valuable insights, but it remains possible that honor concerns and values might be important to a different extent—or in different ways—in cultural contexts with different linguistic and religious heritages and different contemporary economic circumstances, such as Türkiye and Spain. Hence, we sought to compare profiles of honor values and concerns across three regions *within* the Mediterranean area—Latin Europe, Southeastern Europe, and the MENA region—as well as against Anglo-Western and East Asian comparators.

### Capturing the Multifaceted and Gendered Aspects of Honor

Previous research has often ignored the multifaceted and gendered nature of honor when comparing cultural groups. Much work has focused on interpersonal retaliation, highlighting that members of “honor cultures” are more willing than members of “dignity cultures” to retaliate following threats to their reputation (reviewed by Uskul & Cross, 2019). Yet, honor is a multifaceted concept that encompasses other important elements, which may not necessarily co-occur (e.g., concerns about maintaining moral integrity, sexual propriety, dominance, Rodriguez Mosquera, 2016; Saucier et al., 2016). For example, Rodriguez Mosquera and colleagues (2002b) compared four different facets of honor among Dutch and Spanish students, finding a cross-cultural difference in concern for family reputation, a gender difference in concern for “feminine honor,” and no significant culture or gender differences in concerns for moral integrity or “masculine honor.” These elements are beginning to receive more attention in conceptual work on honor (Rodriguez Mosquera, 2016). However, the literature has lacked a large-scale comparison of differences and similarities on these dimensions among cultures thought to foster honor, face, and dignity logics (but see Guerra et al., 2013).

Relatedly, the role of gender in honor-related processes has been under-researched. Acts of retaliation to defend one’s reputation from honor threats have been construed primarily as cultural masculinity norms, leaving women’s perceptions and endorsement of honor within and across cultural groups relatively less studied and understood (but see recent efforts such as those of Glick et al., 2016; Lopez-Zafra et al., 2019; Pomerantz et al., 2023). Here, we defined our cultural samples by the intersection of society and gender, allowing us to explore systematically to what extent women and men within the societies studied could be said to experience different cultural environments in terms of the emphasis placed on honor, face, and dignity in general, as well as four specific dimensions of honor-related concerns: family reputation, family authority, sexual propriety, and integrity (based on the work of Rodriguez Mosquera, 2016).

### Clarifying the Relation Between Honor Logic and Social Cognitive Tendencies

Describing a given society as an “honor culture” implies not only that honor values and concerns should be prevalent but also that honor should play an important role in explaining core aspects of societal members’ social and psychological functioning. The idea that cultural environments foster different ways of being and relating is supported by a long tradition of research, much of which relies on a binary theoretical framework contrasting Western and East Asian cultural emphases on independence versus interdependence (Kitayama et al., 2009; Markus & Kitayama, 1994; but see Kitayama et al., 2022; Vignoles, 2018). Yet, evidence has been lacking regarding how cultural logics of honor, face, and dignity are linked to frequently studied cultural variations in social orientation and cognitive style.

Recently, Uskul, Kirchner-Häusler, and colleagues (2023) reported a distinctive pattern in implicit measures of social orientation and cognitive style among Mediterranean samples that was seemingly inexplicable in terms of independence versus interdependence: Compared to those from East Asian and/or Anglo-Western world regions, participants from Mediterranean societies on average reported a greater tendency to experience socially disengaging emotions (e.g., pride, anger) relative to socially engaging emotions (e.g., feelings of closeness, shame), their happiness was more closely associated with socially disengaging (vs. engaging) positive emotions, and they showed a more “inflated” sense of self (indexed by the relative size of the self, compared to their friends, in a diagram they drew), but they also showed a greater tendency to remember events from a third-person (vs. first-person) perspective. Uskul and colleagues speculated that this pattern of social cognitive tendencies might be explained by a cultural logic of honor, in which people must compete for social standing in unstable hierarchies. Individuals living these contexts might be expected to relate to others as competitors (fostering disengaging emotions and an inflated sense of self) while monitoring their social image carefully for potential threats to their honor (fostering a third-person perspective). However, Uskul et al. did not test the role of honor values in explaining their findings. Here, using measures from the same dataset, we provide a first empirical test of the predictive utility of culture-level variation in perceived and/or personally endorsed honor values to account for the distinctive pattern of social cognitive tendencies they had identified in Mediterranean societies.

### Measuring Cultural Logics Through Personal Endorsement or Perceived Norms

Honor, face, and dignity have been theorized as *cultural* logics but are usually measured through individuals’ *personal* values or concerns. Theoretically, cultural logics are properties of social systems, promoting appropriate behavior through social norms, institutions, traditions, and other socio-cultural processes that are not simply reducible to individuals’ personal values or concerns. Yet, previous research into cultural logics has usually measured individuals’ *personal endorsement* of honor, face, and dignity values or concerns, rather than assessing these constructs as perceived characteristics of the *cultural groups or societies* they inhabit. Recent research has begun to address this by treating participants as “informants” about their local cultural contexts, asking them to rate their perceptions of the normative values in their respective societies rather than rating their personal values (P. B. Smith et al., 2017; Yao et al., 2017). These measures are not necessarily statistically accurate predictions of the values or concerns that other cultural members endorse, but they reflect participants’ lived experiences of the cultural contexts they inhabit. Here, we sought to test systematically the unique insights that measuring perceived societal norms versus personal endorsement may provide when mapping cultural differences in honor, face, and dignity logics and in accounting for differences in psychological functioning. Moreover, we used multilevel models to separate genuinely culture-level variance in both sets of measures from compositional effects of individual differences.

## The Current Studies

We report three sets of analyses addressing the goals described above, using data from two large-scale cross-cultural studies. In Study 1, we explored the prevalence of honor values and specific honor-related concerns across diverse Mediterranean societies within a broader mapping of perceived normative and personally endorsed honor, face, and dignity values and concerns among university student samples from 11 societies: seven in the Mediterranean region (Spain, Italy, Greece, Greek Cypriot community in Cyprus, Türkiye, Lebanon, Egypt), two in East Asia (Japan, Korea), and two Anglo-Western societies (the United Kingdom, the United States). In Study 2, we narrowed the focus to honor values, replicating key findings from Study 1 among general population samples and across more societies (all Study 1 sites, plus Canada, Tunisia, the Turkish Cypriot Community in Cyprus). For Study 3, we used additional measures from the Study 1 dataset, testing the ability of honor values to account for previously reported cultural differences between Mediterranean and non-Mediterranean societies in measures of social orientation and cognitive style. These studies were not preregistered.

Thus, we set out to answer four main research questions (RQs):

RQ1. Are Mediterranean, East Asian, and Anglo-Western regions characterized, respectively, by higher perceived normativity, as well as personal endorsement, of honor, face, and dignity values (Studies 1/2) and concerns (Study 1), as commonly assumed?
RQ2. What is the extent of variation in perceived normative and personally endorsed honor values (Studies 1/2) and honor-related concerns (Study 1) across different cultural subregions within the Mediterranean, which have been characterized as “cultures of honor” in past social science research?
RQ3. To what extent do cultural samples of women and men differ in perceiving as normative, and personally endorsing, specific honor-related concerns (Study 1), as well as honor values more generally (Studies 1/2)?
RQ4. To what extent can cultural variation in perceived normativity and/or personal endorsement of honor values help explain distinctive tendencies in social orientation and cognitive style previously observed among members of Mediterranean societies (Study 3)?

## Study 1

Measures were included in a large-scale multinational study focusing on honor, face, and dignity logics, social cognitive tendencies, self-construals, and well-being (see also Kirchner-Häusler et al., 2023, 2024; Psaltis et al., 2023; Uskul, Kirchner-Häusler, et al., 2023).^2^ We measured participants’ personal values and concerns related to honor, face, and dignity, as well as their perceptions of normative values and concerns in their societies. To address RQ1 to RQ3, we mapped variations across 22 cultural samples defined by the intersection of society and gender, using multilevel modeling to separate culture-level variation from compositional effects of individual differences.

### Method

#### Participants

In 11 sites, 4,583 participants (*Min* = 261 in Nagoya, Japan, and the U.K., *Max* = 767 in Crete, Greece) answered our survey between December 2019 and February 2021. Given the difficulties of assessing statistical power for such a complex and multifaceted study, we defined target sample sizes by seeking to match or exceed those in most previous research into honor cultures (Salvador et al., 2020; San Martin et al., 2018; P. B. Smith et al., 2017). Thus, we aimed for samples of at least 100 men and 100 women per country (resources and COVID-related restrictions permitting); we met our goal in all but two countries (Egypt and Lebanon, each ≥ 95 men). We recruited samples primarily via participant pools of collaborating institutions; in the U.K., we also recruited student participants using Prolific to reach the targeted sample size (34.3% of U.K. participants). At different sites, participants received course credit, money, a COVID-related charity donation, or a raffle entry.

Participants had to be (a) at least 18 years old, (b) born in the country of data collection, and (c) residents in the country of data collection for at least half their lives. For analyses, we included only participants who self-identified as (d) members of the majority ethnic group of the respective country (e.g., White British in the UK; Spanish in Spain), and (e) female or male. Since our cultural samples were defined by the intersection of society with female and male gender categories, we could not include participants who identified as non-binary or who did not specify their gender identity. Finally, we excluded participants who failed one or more of four attention checks included across the survey. Thus, we retained 2,942 participants for our analyses (see Table 1 for sample sizes and characteristics per site).

**Table 1.** Overview of Sample Characteristics (Study 1).

Research Site	Women			Men			Language	Data Collection	Local Institution	Compensation
	*n*	Age	SES	*n*	Age	SES				
Cyprus	214	20.44 (2.32)	6.06 (1.2)	103	21.83 (2.18)	6.00 (1.17)	Greek	Online, In-Lab	University of Cyprus	Course Credit, Raffle
Egypt	110	20.66 (1.56)	6.58 (1.07)	95	20.81 (1.56)	6.28 (1.52)	Arabic	Online	British University of Egypt	Donation to Charity
Greece	196	22.79 (5.85)	6.10 (1.22)	284	23.38 (6.22)	6.00 (1.21)	Greek	Online	University of Crete	Course Credit
Italy	135	21.61 (2.56)	5.97 (1.31)	112	24.14 (5.03)	5.81 (1.47)	Italian	Online, In-Lab	University of Chieti-Pescara	Course Credit
Japan	114	20.57 (2.49)	6.10 (1.34)	105	20.36 (1.00)	6.11 (1.63)	Japanese	Online, In-Lab	University of Nagoya	Monetary
Korea	101	21.91 (2.91)	6.35 (1.64)	105	22.88 (2.67)	6.04 (1.70)	Korean	Online	Sogang University	Online Voucher
Lebanon	165	19.06 (1.72)	6.78 (1.34)	96	19.27 (1.47)	6.57 (1.51)	English	Online	American University of Beirut	Course Credit
Spain	116	20.50 (3.30)	6.03 (1.34)	124	24.43 (7.27)	5.43 (1.53)	Spanish	Online	University of Granada	Course Credit
Türkiye	241	20.78 (1.42)	5.66 (1.27)	111	20.86 (1.93)	5.59 (1.32)	Turkish	Online	Bolu Abant Izzet Baysal University Ordu University	Course Credit
UK	104	19.64 (1.52)	5.56 (1.35)	103	20.86 (2.29)	5.65 (1.43)	English	Online, In-Lab	University of Kent	Course Credit, Monetary
US	105	19.21 (1.91)	5.98 (1.43)	103	19.95 (4.28)	6.43 (1.42)	English	Online, In-Lab	Iowa State University	Course Credit
Total	1601	20.72 (3.09)	6.08 (1.35)	1341	22.00 (4.62)	5.98 (1.45)	-	-	-	-

*Note.* Presented are the sample characteristics for cultural samples of women and men from the 11 included research sites in Study 1. For age and SES, the mean is presented with the standard deviation in brackets. Data in Cyprus were collected from the Greek Cypriot community

The overall gender distribution was balanced (54.4% women; *M_age_* = 21.31, *SD* = 3.91, range: 18 to 69). Self-reported socio-economic status (SES: *M* = 6.03, *SD* = 1.40) averaged slightly above the midpoint of an 11-point scale from 0 (*Bottom*) to 10 (*Top*). In 9 out of 11 sites, most participants reported having lived exclusively in urban environments (53.43% across complete sample, vs. rural environments [23.25%] or both [23.32%]); however, most Korean participants (83.5%) and nearly half of Japanese participants (44.75%) reported having lived mainly in rural environments.

#### Procedure

We invited participants to take part in an online study, which they completed in the lab (18.95%) or on their own devices (81.05%).^3^ After providing consent, participants completed numerous tasks as part of a larger study on cultural differences in social orientation and cognitive style (Uskul, Kirchner-Häusler, et al., 2023; see also Study 3 of the current paper). Next, participants completed the key measures analyzed here, focusing on their personal endorsement of honor, face, and dignity concerns and values, as well as the corresponding perceived societal norms. Finally, participants provided demographic information and were thanked and debriefed. The study received approval from ethical committees of all collaborating institutions.

#### Measures

All materials were originally collated and developed in English. We used a team translation approach: tasks were translated by native speakers of the respective languages and then reviewed for accuracy and local language conventions by other team members fluent in both the local language and English (Survey Research Center, 2022). In case of disagreement, further individuals were consulted to reach a final version.

##### Honor, Face, and Dignity Values

Participants rated 22 items assessing their personal endorsement of honor, face, and dignity values, using a 7-point scale (1 = *strongly disagree* to 7 = *strongly agree*). Sixteen items were developed by Yao and colleagues (2017) to measure values of dignity (six items; e.g., “*People should be true to themselves regardless of what others think.*”), face (six items; e.g., “*People should be very humble to maintain good relationships.*”), and honor (four items; e.g., “*People should not allow others to insult their family.*”). To increase coverage of honor values, we added six items from the study by Smith and colleagues (2017) (e.g., “*People always need to show off their power in front of their competitors.*”). We slightly rephrased some items to capture endorsement of cultural values rather than states or behaviors. Participants rated their personal agreement with these items (i.e., *personal values*), followed by how much they perceived most people in their society would agree or disagree (i.e., *perceived normative values*).

##### Honor, Face, and Dignity Concerns

Participants completed the 16-item Honor Scale by Guerra and colleagues (2013; abridged from Rodriguez Mosquera et al., 2002b). Items ask to what extent behaving in a specific way or having a specific reputation would make participants feel bad about themselves (e.g., “*How bad would you feel about yourself if … you did something to damage your family’s reputation?*”). We devised eight additional items assessing face (four items, e.g., “*… you failed to show humility about your achievements?*”) and dignity concerns (four items, e.g., “… *you did not stand up for what you believe?*”), based on central goals for face cultures (i.e., harmony, humility, self-moderation) and dignity cultures (i.e., authenticity, self-reliance, self-worth) (Leung & Cohen, 2011). Participants rated these items twice: indicating first how bad they themselves would feel (i.e., *personal concerns*) and then how badly they perceived most people in their society would feel (i.e., *perceived normative concerns*).

##### Demographic Information

Participants in all cultures reported their gender, age, country of birth, country where they attended high school, parents’ country of birth, parents’ highest education, length of stay in the country of data collection, native language, type of environment they mainly lived in (urban, rural, both), religious background, religiosity, and their perceived SES in the country of residence (MacArthur Scale of Subjective Social Status: Adler et al., 2000). Self-reported ethnicity was assessed in all countries except Lebanon and Egypt. Demographic materials were adjusted by local collaborators to assess locally meaningful categories (e.g., ethnic background was excluded where the local political climate may prevent sharing such information).

#### Measurement Models

We tested multilevel measurement models to confirm the internal structure of our measures and refine their validity for use in our main analyses (see Supplemental Materials for full details). Given the importance of gender norms in conceptualizations of honor (Lopez-Zafra et al., 2019) and the extent of gender segregation in some societies studied here (Bussemakers et al., 2017), we considered women and men in each society as potentially experiencing different cultural environments. Hence, we defined cultural samples by the intersection of gender and society. We did not include individual-level covariates in these models. All models included a method factor at each level to adjust for individual and cultural response styles.

For both perceived normative and personal *values*, measurement models revealed three substantive between-samples (i.e., culture level) factors comprising dimensions of *honor values*, *face values*, and *dignity values*. At the within-samples level, honor values formed two distinct factors, focused on (a) defending family reputation and (b) self-promotion and retaliation (see Supplemental Tables S1–S3 and S7–S9). Note that our main analyses focus on the between-samples part of these models, reflecting our RQs.

Perceived normative and personal *concerns* showed a multidimensional structure, consistent with previous research (Guerra et al., 2013) and theory (Rodriguez Mosquera, 2016). Our measurement model for perceived normative concerns revealed a six-factor structure at both levels of analysis, comprising variation in concerns about losing *dignity*, *face*, and four distinct bases of honor: *family reputation*, *family authority*, *sexual propriety*, and *integrity*.^4^ Our measurement model for personal concerns showed only four culture-level factors, comprising variation in *concern about losing dignity* and in concerns about losing three distinct bases of honor: *family reputation*, *family authority*, and *sexual propriety*. Personal concerns about losing face and losing integrity showed within-sample differences but no measurable culture-level variation (see Supplemental Tables S4–S6 and S10–S12).

### Results

To address RQ1–RQ3, our main analyses focused on modeling *culture-level* variation in perceived normativity and personal endorsement of honor, face, and dignity values and concerns across 22 cultural samples (i.e., women and men from 11 societies).

#### Regional Comparisons

**Figure 1.**
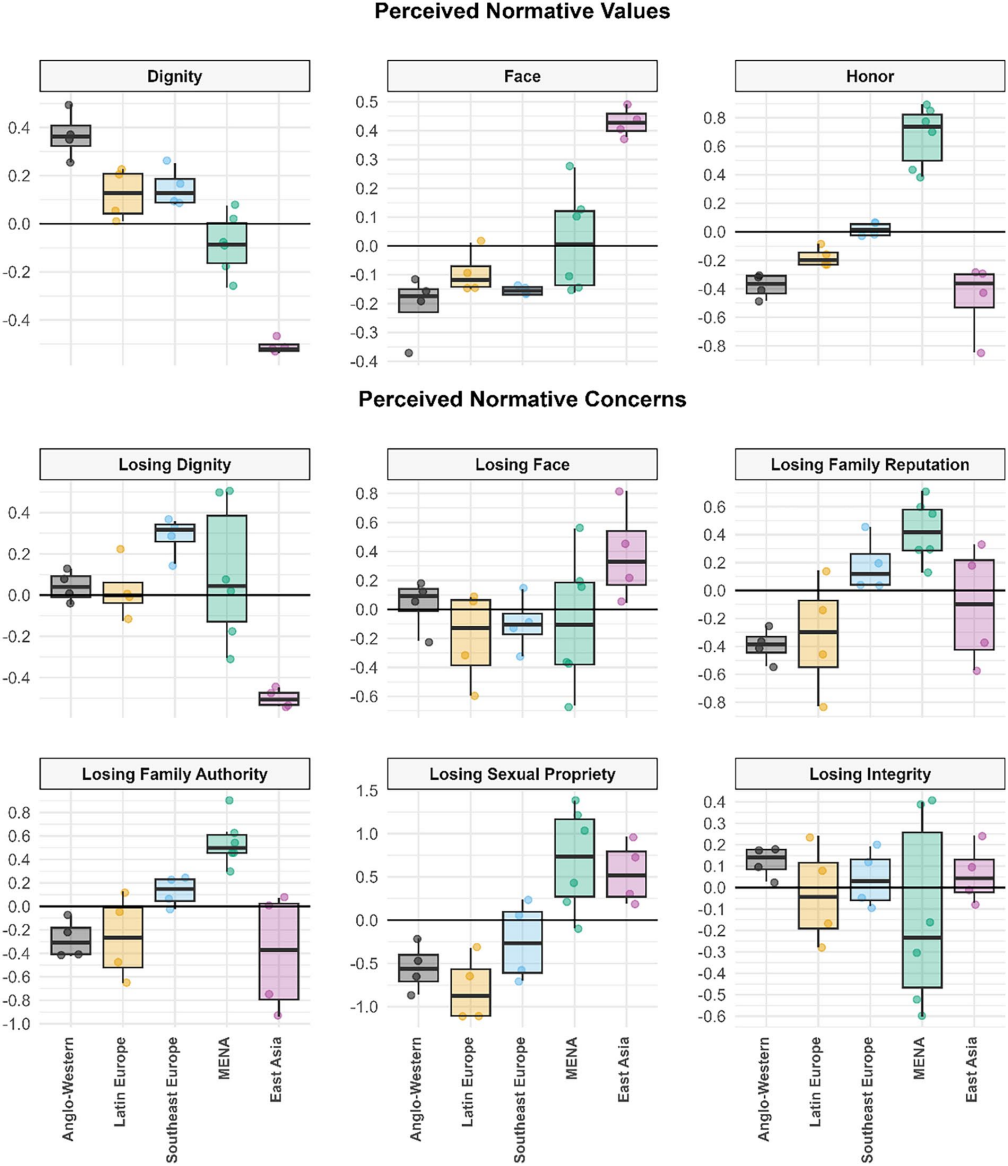
Boxplots Showing Perceived Normativity of Dignity, Face, and Honor Values and Concerns Across Five Geographical Regions (Study 1). *Note*. Boxplots are based on the higher-level factor scores for all 22 cultural samples (defined by the intersection of country × gender), grouped into five larger regions. The black line within each box represents the respective region median. Boxes designate the range of the inner 50% of samples (i.e., interquartile range), whereas the whiskers represent the outer 50% of samples. *Anglo-Western:* United States, United Kingdom; *Latin Europe*: Spain, Italy; *Southeast Europe*: Greece, Greek Cypriot community; *MENA*: Türkiye, Lebanon, Egypt; *East Asia*: South Korea, Japan.

**Figure 2.**
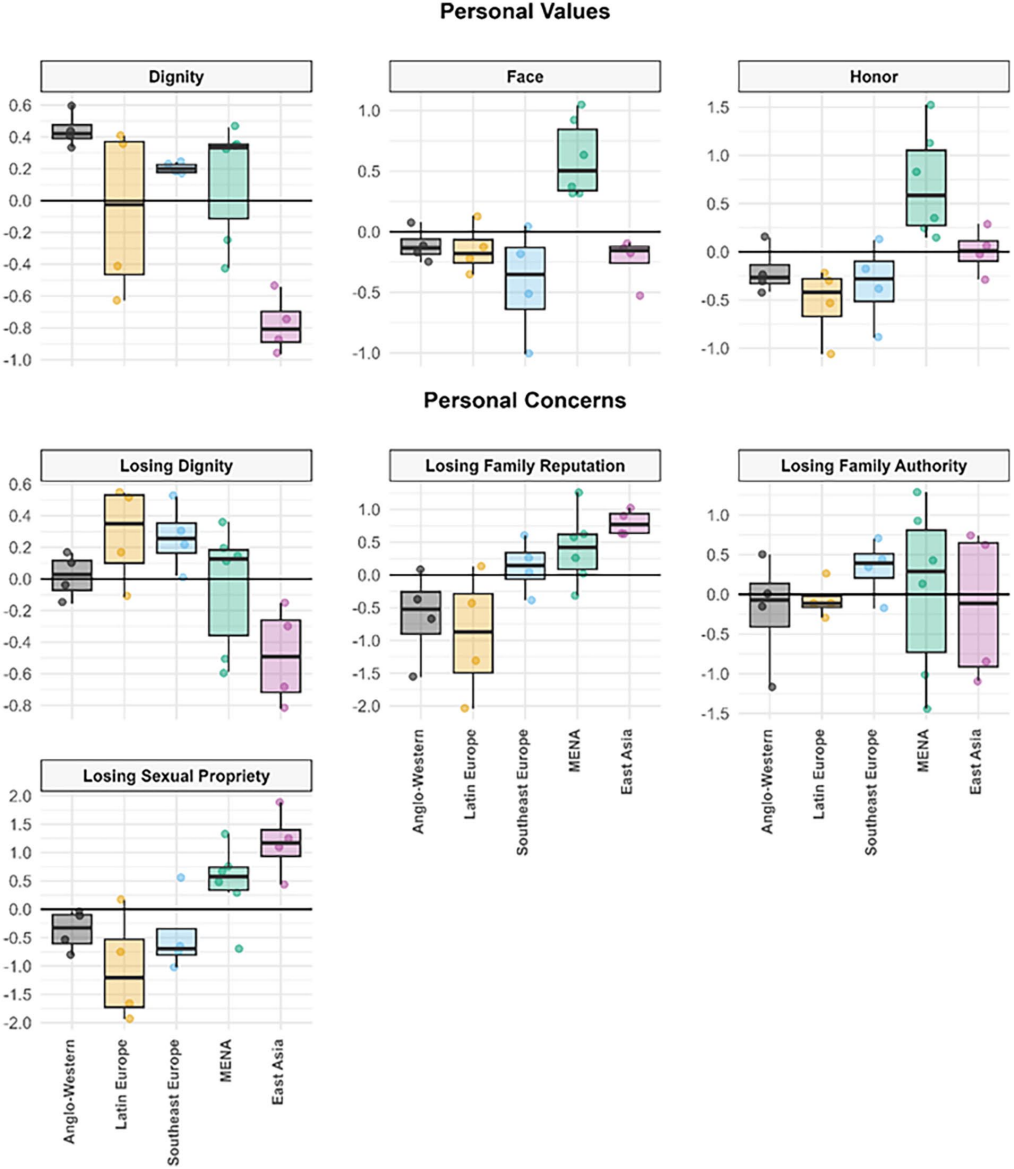
Boxplots Showing Personal Endorsement of Dignity, Face, and Honor Values and Concerns Across Five Geographical Regions (Study 1). *Note*. Boxplots are based on the higher-level factor scores for all 22 cultural samples (defined by the intersection of country × gender), grouped into five larger regions. The black line within each box represents the respective region median. Boxes designate the range of the inner 50% of samples (i.e., inter-quartile range), whereas the whiskers represent the outer 50% of samples. *Anglo-Western:* United States, United Kingdom; *Latin Europe*: Spain, Italy; *Southeast Europe*: Greece, Greek Cypriot community; *MENA*: Türkiye, Lebanon, Egypt; *East Asia*: South Korea, Japan.

To examine RQ1 and RQ2, we grouped our 22 cultural samples into five cultural regions (*Anglo-Western*: the U.K., the U.S.; *Latin Europe*: Spain, Italy; *Southeast Europe*: Greece, Cyprus [Greek Cypriot Community]; *MENA*: Türkiye, Egypt, Lebanon; *East Asia*: South Korea, Japan) using an existing taxonomy reflecting countries’ ethnic, religious, and linguistic background, and their geographic proximity (Mensah & Chen, 2012), thus dividing our Mediterranean samples into three subregions. Graphs illustrate the regional patterns in perceived normativity (Figure 1) and personal endorsement (Figure 2) of dignity, face, and honor values and concerns, using factor scores saved from our measurement models.

To test the statistical significance of pairwise regional differences, we adapted our measurement models by regressing the culture-level latent factors onto sets of four contrasts distinguishing the five regions. For each dimension, we corrected for familywise error among the 10 possible pairwise comparisons using a Holm-Bonferroni sequentially adjusted alpha level starting at .05/10 = .005 (Holm, 1979); however, to guard against Type II error, we interpret as “marginal” those nonsignificant findings that nonetheless reached *p* ≤ .05. Table 2 summarizes these results (see Supplemental Tables S14–S17 for full details).

**Table 2.** Relative Levels of Endorsement of Value and Concern Dimensions for each Region (Study 1).

Variable	Anglo-Western		Latin Europe		Southeast Europe		MENA		East Asia	
	*M*	*SE*	*M*	*SE*	*M*	*SE*	*M*	*SE*	*M*	*SE*
**Perceived normative values**										
Dignity	0.476_a_	0.006	0.066_bc_	0.008	0.116_b_	0.016	−0.071_c_	0.003	−0.587_d_	0.034
Face	−0.060_a_	0.002	−0.192_a_	0.007	−0.214_a_	0.010	0.087_ab_	0.009	0.377_b_	0.068
Honor	−0.238_a_	0.003	−0.169_a_	0.007	0.061_b_	0.005	0.799_c_	0.007	−0.455_a_	0.089
**Perceived normative concerns**										
Losing Dignity	−0.007_a_	0.073	0.036_a_	0.058	0.346_b_	0.059	0.120_ab_	0.130	−0.495_c_	0.076
Losing Face	−0.096_a_	0.144	−0.259_a_	0.139	−0.026_a_	0.101	−0.126_a_	0.177	0.508_a_	0.214
Losing Family Reputation	−0.458_a_	0.095	−0.308_a_	0.130	0.281_b_	0.121	0.501_b_	0.093	−0.016_ab_	0.224
Losing Family Authority	−0.330_a_	0.104	−0.255_a_	0.127	0.232_b_	0.076	0.642_c_	0.086	−0.289_a_	0.156
Losing Sexual Propriety	−0.561_a_	0.142	−0.761_a_	0.140	−0.154_a_	0.189	0.814_b_	0.194	0.663_b_	0.179
Losing Integrity	0.038_a_	0.079	−0.078_a_	0.145	0.058_a_	0.065	−0.116_a_	0.179	0.099_a_	0.113
**Personal values**										
Dignity	0.490_a_	0.070	−0.066_b_	0.198	0.208_b_	0.062	0.153_b_	0.128	−0.785_c_	0.090
Face	−0.028_a_	0.108	−0.002_a_	0.104	−0.480_a_	0.192	0.731_b_	0.110	−0.221_a_	0.104
Honor	−0.101_a_	0.124	−0.528_b_	0.161	−0.269_ab_	0.169	0.804_c_	0.190	0.094_a_	0.120
**Personal concerns**										
Losing dignity	−0.043_ab_	0.098	0.262_a_	0.159	0.316_a_	0.131	0.003_ab_	0.150	−0.538_b_	0.157
Losing family reputation	−0.667_a_	0.291	−0.913_a_	0.344	0.232_ab_	0.198	0.502_bc_	0.229	0.846_c_	0.131
Losing family authority	−0.231_a_	0.305	−0.077_a_	0.177	0.369_a_	0.202	0.066_a_	0.377	−0.127_a_	0.405
Losing sexual propriety	−0.382_a_	0.185	−1.041_a_	0.327	−0.374_ab_	0.250	0.581_bc_	0.274	1.216_c_	0.280

*Note*. Scores for each region were taken from the final measurement model with added deviation-coded region indicators and indicate how each region differs from the mean of the five regions. Means within each row that do not share a subscript differed significantly in post hoc pairwise comparisons using dummy-coded region indicators with Holm-Bonferroni adjustment. See Supplemental Tables S14–S17 for details of all pairwise comparisons. *Anglo-Western Heritage*: United States, United Kingdom; *Latin Europe*: Spain, Italy; *Southeast Europe*: Greece, Greek Cypriot community; *MENA*: Türkiye, Lebanon, Egypt; *East Asia*: South Korea, Japan.

##### Perceived Normative Values and Concerns

As shown in the top row of Figure 1, the five regions differed in *perceived normative values* largely as expected, but with some notable geographical variation across the three Mediterranean subregions.

As expected, Anglo-Western samples perceived *dignity values* as significantly more normative than samples from all other regions (*p*s < .001), whereas East-Asian samples perceived dignity values as significantly less normative than samples from all other regions (*p*s < .001); samples from the three Mediterranean regions showed varying intermediate levels. As expected, East-Asian samples perceived *face values* as the most normative, significantly or marginally higher than samples from all other regions (range of *ps*: <.001–.032), which did not differ significantly from each other (see Table 2).

Crucially, perceived normative *honor values* showed a very different pattern: MENA samples perceived honor values as significantly more normative than samples from all other regions (*p*s < .001). Southeast European samples, in turn, perceived honor values as more normative than the remaining regions (*p*s ≤ .006). Perceived normativity of honor values in Latin-European samples was comparable to that in Anglo-Western samples (*p* = .457), and only marginally stronger than that in East Asian samples (*p* = .028). Thus, Mediterranean samples collectively perceived relatively high normative honor values (RQ1), but these perceptions varied with geographical position around the Mediterranean basin: strongest among MENA samples and weakest among Latin-European samples (RQ2).

As shown in the middle and bottom rows of Figure 1, the patterning of *perceived normative concerns* was comparable to perceived normative values, but with certain notable differences. As expected, Anglo-Western samples perceived stronger normative concern about losing dignity than East-Asian samples (*p* < .001). However, Southeast European samples perceived the highest normative concern about losing dignity, significantly stronger than that in all other regions (*p*s < .001) except the MENA region (*p* = .17). In contrast, East Asian samples perceived significantly weaker normative concern about losing *dignity* than samples from all other regions (*p*s ≤ .001). Directionally consistent with expectations, East-Asian samples perceived marginally stronger normative concern about losing *face* than all other regions (*p*s ≤ .05), yet no comparison reached the Holm-Bonferroni significance threshold.

Perceived normative *honor-related concerns* about losing *family reputation*, *family authority*, and *sexual propriety* showed a similar pattern to perceived normative honor values: MENA samples generally perceived the strongest normative concerns about losing all three forms of honor—significantly stronger than the other four regions in most cases (*p*s ≤ .002), except that they differed nonsignificantly from Southeast European (*p* = .134) and East-Asian samples (*p* = .072) in concern about losing family reputation, and from East-Asian samples in concern about losing sexual propriety (*p* = .621). Anglo-Western, East Asian, and Latin-European samples mostly perceived the weakest normative concerns for these three forms of honor. However, East-Asian samples perceived significantly stronger normative concern about losing sexual propriety than all except MENA samples (*p*s ≤ .006). Finally, samples did not differ significantly in perceived normative concern about losing integrity.

##### Personal Values and Concerns

Culture-level variation in personal endorsement of honor, face, and dignity values and concerns (Figure 2) showed similarities but also some notable differences, compared to perceived normative values and concerns.

Personal endorsement of dignity values and concerns was mostly consistent with the pattern of perceived normative values and concerns. Anglo-Western samples personally endorsed *dignity values* most strongly of all regions—significantly or marginally higher than all other regions (*ps* < .028), whereas East-Asian samples’ endorsement of dignity values was significantly lower than that in all other regions (*p*s ≤ .005). East-Asian samples also showed marginally or significantly weaker *concerns about losing dignity* than samples from all other regions (*ps* < .030). However, personal concerns about losing dignity were at least as high in all three Mediterranean regions as in Anglo-Western samples.

Although members of East-Asian cultures distinctively perceived *face values* were normative in their societies (described earlier), they did not distinctively endorse these values themselves. Unexpectedly, MENA samples personally endorsed face values significantly more strongly than samples from all other regions (*p*s < .001), among which there were no significant differences (*p*s ≥ .062).

Consistent with their perceived normative values, MENA samples showed the strongest personal endorsement of honor values, significantly stronger than all other regions (*p*s ≤ .005). Unexpectedly, Latin-European samples showed the lowest personal endorsement of honor values—comparable to Southeast European (*p* = .332) and Anglo-Western samples (*p* = .053), and significantly lower than East-Asian samples (*p* = .004).

Finally, personal concerns about losing *family reputation* and *sexual propriety* were highest not only in MENA samples but unexpectedly also in East-Asian samples, whereas they were lowest in Anglo-Western and Latin-European samples (all comparisons between the former two regions and the latter two regions were significant: *p*s ≤ .006). Southeast European samples showed relatively high personal concern about losing family reputation, comparable to MENA samples (*p* = .394), but lower concern about losing sexual propriety, similar to Anglo-Western and Latin-European samples (*p*s ≥ .160). Unlike perceived normative concerns, personal concern about losing *family authority* was largely similar across all regions, albeit marginally higher in Southeast Europe than in Latin Europe (*p* = .028).

#### Gender Comparisons

**Figure 3.**
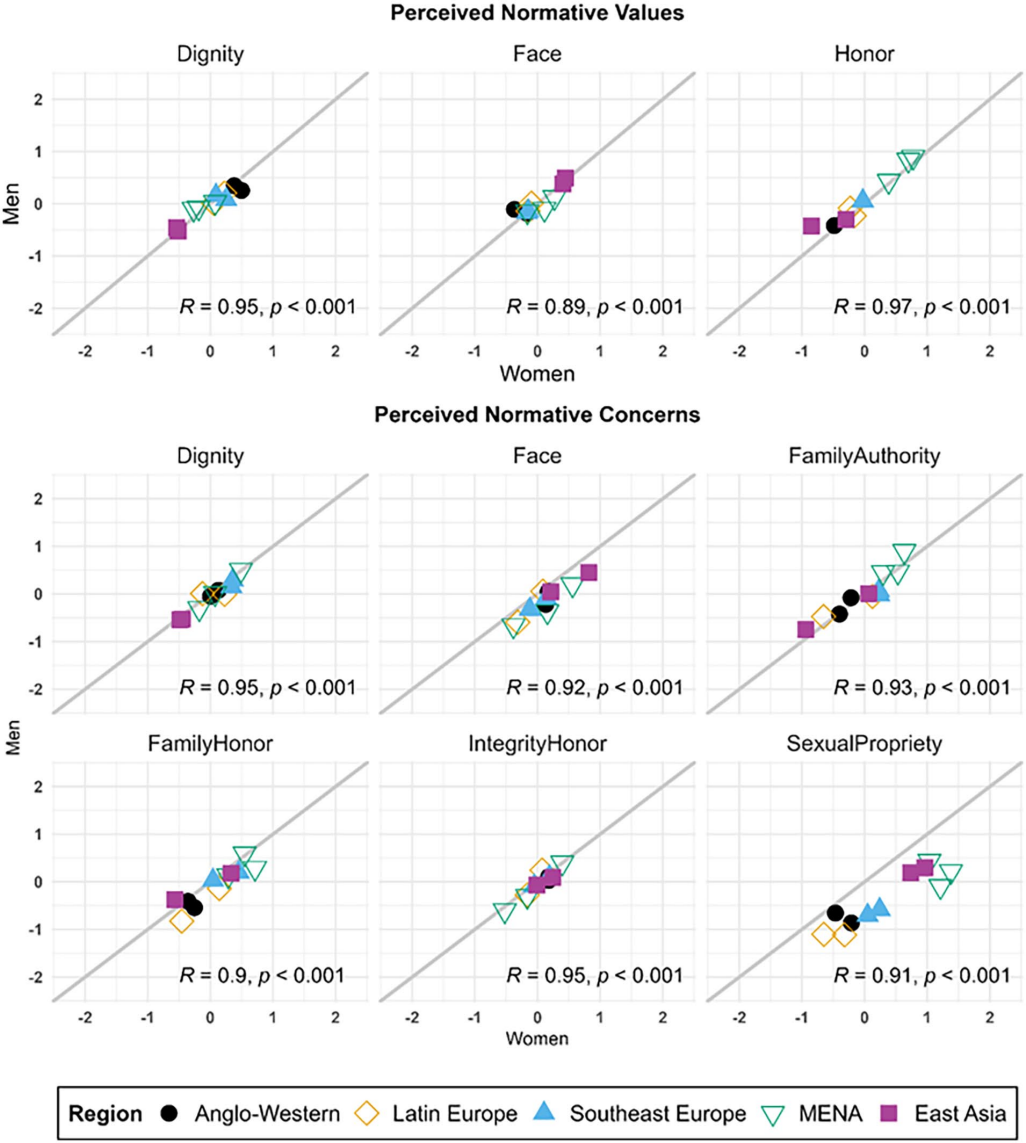
Comparison of Culture-Level Factor Scores for Cultural Samples of Women and Men Across Perceived Normative Endorsement of Dignity, Face, and Honor Values and Concerns (Study 1). *Note*. Shown are scores for perceived normative values and concerns among women and men in all samples. The gray line indicates where gender groups from the same society would have equal scores. Values in the lower right corner are Pearson correlations between gender groups across societies (*N* = 11; all *ps* < .001).

**Figure 4.**
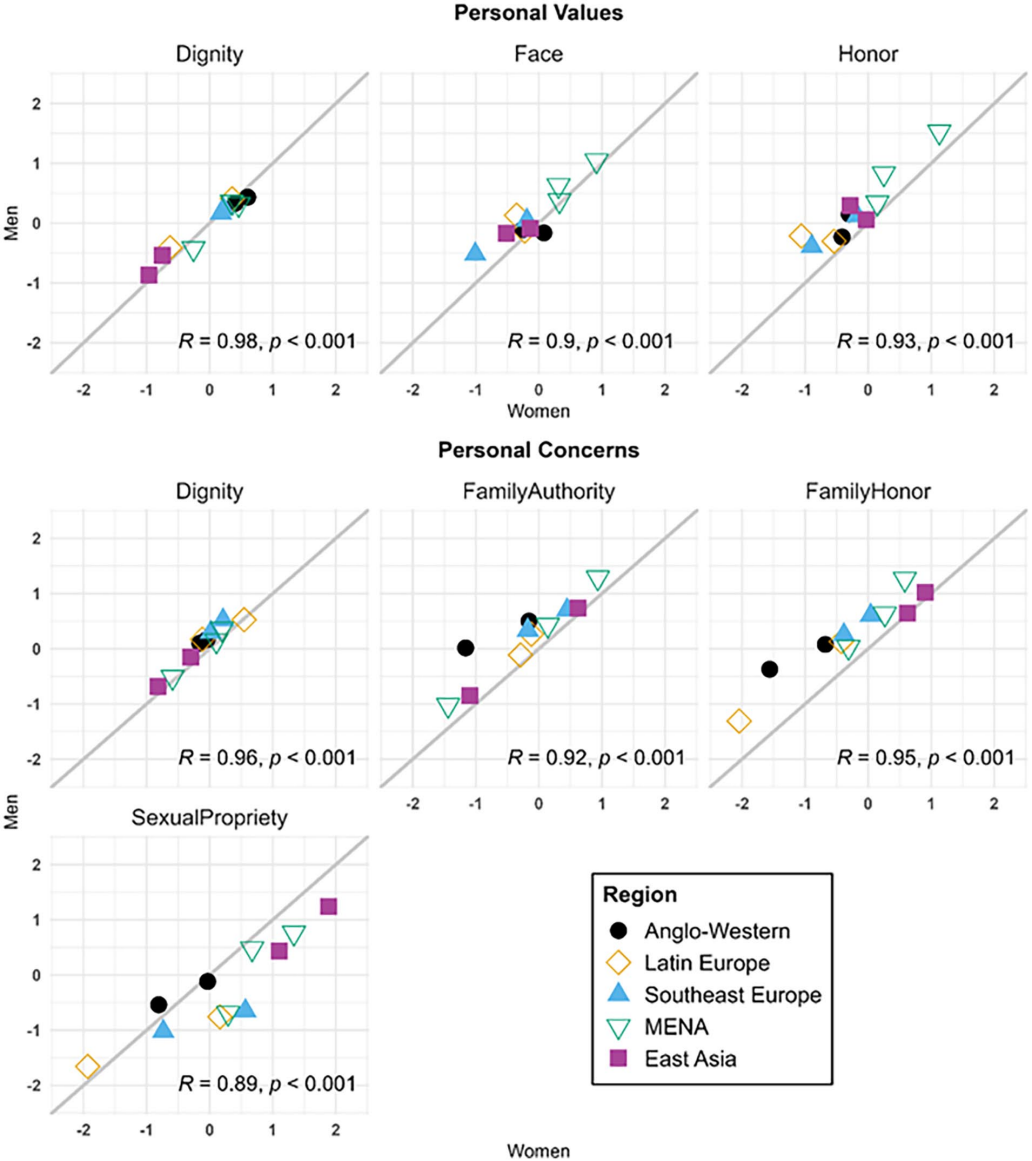
Comparison of Culture-Level Factor Scores for Cultural Samples of Women and Men Across Personal Endorsement of Dignity, Face, and Honor Values and Concerns (Study 1). *Note*. Shown are scores for personal values and concerns among women and men in all samples. The gray line indicates where gender groups from the same society would have equal scores. Values in the lower right corner are Pearson correlations between gender groups across societies (*N* = 11; all *ps* < .001).

As seen in Figures 3 and 4, all dimensions were remarkably consistent across women and men in all societies. Correlations between scores for men and women were highly positive and significant for all variables (*p*s < .001), ranging from .890 (perceived normative face values) to .978 (personal dignity values), indicating very high rank-order consistency between gendered samples from each of the 11 societies in our study.

To test for gender differences in mean levels of perceived normative and personal values and concerns (RQ3), we added gender into our final measurement models as a dummy-coded predictor of all culture-level latent factors (see Supplemental Table S18 for full results). Only one difference reached significance: Women perceived significantly more than men that people in their societies would be concerned about losing sexual propriety (*b* = 0.761, *p* = .008, *d* = 0.909).

### Discussion

As expected, Mediterranean groups perceived that honor values were more normative in their societies than groups from East Asian and Anglo-Western regions—whereas groups from the latter two regions respectively perceived that face and dignity values were more normative. Cultural groups in MENA societies perceived the greatest normative prevalence of honor values in their societies, followed by those in Southeast European societies—whereas Latin-European cultural groups perceived a comparable prevalence of honor values to those in Anglo-Western and East-Asian societies. Cultural groups’ perceptions of the normative prevalence of specific honor-related concerns followed a similar pattern, although concerns about losing integrity did not vary significantly across regions. In contrast, personal endorsement of values and concerns showed more complex regional patterns, highlighting that cultural logics are not reducible to individuals’ personal endorsements. These cultural patterns were remarkably consistent across women and men, although samples of women on average reported that people in their societies would be more concerned about losing sexual propriety.

## Study 2

In Study 2, we replicated key findings of Study 1 among general population samples from 14 societies (all Study 1 sites plus Canada, Tunisia, and the Turkish Cypriot Community in Cyprus), mapping culture-level variation in perceived normativity and personal endorsement of honor values among 5,471 participants from 28 cultural samples defined by the intersection of society and gender (see Table 3). A full report of Study 2 Method and Results can be found in Supplemental Materials.

**Table 3.** Overview of Sample Characteristics (Study 2).

Research Site	Women			Men			Language
	*n*	Age	SES	*n*	Age	SES	
Canada	210	48.21 (15.81)	6.04 (1.72)	197	48.88 (16.01)	6.16 (1.71)	English
Cyprus (Greek Cypriot)	132	44.11 (15.60)	5.41 (1.55)	147	47.46 (15.24)	5.81 (1.64)	Greek
Cyprus (Turkish Cypriot)	188	41.83 (13.24)	5.72 (2.07)	213	45.46 (13.31)	5.85 (2.03)	Turkish
Egypt	196	32.15 (8.71)	5.56 (1.95)	200	32.95 (10.53)	5.12 (1.99)	Arabic
Greece	200	41.52 (13.09)	5.52 (1.59)	200	46.26 (13.43)	5.39 (1.80)	Greek
Italy	200	40.42 (16.34)	5.81 (1.62)	200	45.62 (17.13)	5.85 (1.59)	Italian
Japan	199	47.85 (12.68)	4.91 (1.88)	200	51.16 (14.49)	4.80 (1.99)	Japanese
Korea	200	42.03 (12.53)	4.88 (1.87)	198	46.61 (13.76)	4.76 (2.08)	Korean
Lebanon	198	31.22 (9.29)	5.40 (2.14)	200	31.88 (10.66)	5.01 (1.99)	English
Spain	200	42.81 (13.03)	6.05 (1.62)	198	45.80 (15.54)	5.81 (1.64)	Spanish
Tunisia	197	31.27 (8.38)	5.27 (1.97)	200	37.42 (11.16)	5.41 (1.71)	Arabic
Türkiye	200	36.09 (10.83)	6.29 (1.82)	200	40.51 (14.53)	6.14 (1.87)	Turkish
UK	200	49.15 (14.19)	5.15 (1.89)	200	51.07 (18.84)	5.45 (1.92)	English
US	199	46.85 (15.96)	5.86 (2.37)	199	47.40 (16.61)	7.06 (2.09)	English
Total	2719	41.08 (14.37)	5.57 (1.92)	2752	44.11 (15.69)	5.61 (1.96)	-

*Note.* Presented are the sample characteristics for cultural samples of women and men from the 14 included research sites in Study 2. For age and SES, the mean is presented with the standard deviation in brackets.

**Figure 5.**
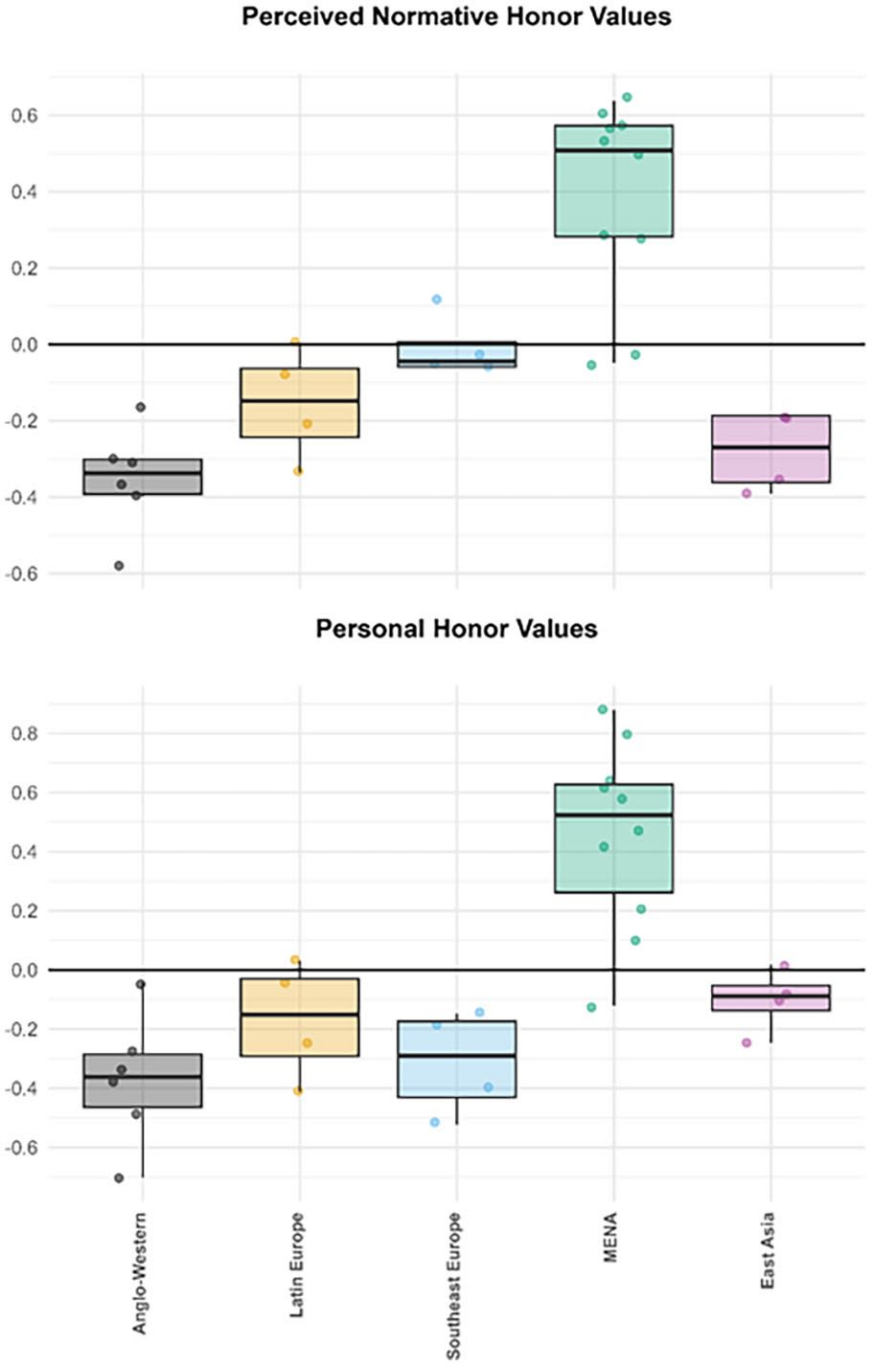
Boxplots Showing Perceived Normativity and Personal Endorsement of Honor Values Across Five Geographical Regions (Study 2). *Note*. Boxplots are based on the higher-level factor scores for all 28 cultural samples (defined by the intersection of country × gender), grouped into five larger regions. The black line within each box represents the respective region median. Boxes designate the range of the inner 50% of samples (i.e., interquartile range), whereas the whiskers represent the outer 50% of samples. *Anglo-Western*: United States, United Kingdom, Canada; *Latin Europe*: Spain, Italy; *Southeast Europe*: Greece, Greek Cypriot community; *MENA*: Türkiye, Turkish Cypriot community; Lebanon, Egypt, Tunisia; *East Asia*: South Korea, Japan.

**Figure 6.**
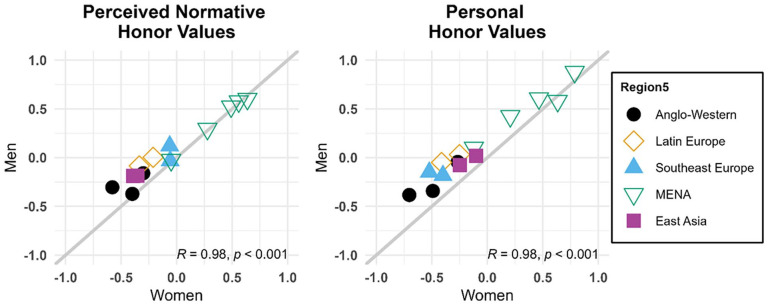
Comparison of Culture-Level Factor Scores for Cultural Samples of Women and Men Across Perceived Normative and Personal Honor Values (Study 2). *Note*. Shown are scores for women and men in all samples. The gray line indicates where gender groups from the same society would have equal scores. Values in the lower right corner are Pearson correlations between gender groups across societies (*N* = 14; all *ps* < .001).

Measurement models adjusting for individual and cultural response styles replicated the previously observed multilevel factor structure of honor values. (As in Study 1, we did not include individual-level covariates in these models.) Figure 5 and Table 4 show that regional differences closely matched those we had observed in Study 1. Perceived normative and personally endorsed honor values were again remarkably consistent across women and men (see Figure 6). Among the 22 cultural groups sampled in both studies, factor scores representing culture-level variance in honor values were highly correlated across studies (perceived normative honor values: *r* = .90; personally endorsed honor values: *r* = .82). This close convergence supports the validity of relying on student participants in Study 1, especially as informants about the perceived normativity of honor values in the societies they inhabited.

**Table 4.** Relative Levels of Endorsement of Value and Concern Dimensions for Each Region (Study 2).

Region	Perceived normative Honor values		Personal Honor values	
	*M*	*SE*	*M*	*SE*
Anglo-West	−0.293_a_	0.046	−0.289_a_	0.071
Latin Europe	−0.096_ab_	0.062	−0.090_ab_	0.082
Southeast Europe	0.074_b_	0.046	−0.221_ab_	0.072
MENA	0.500_c_	0.072	0.583_c_	0.088
East Asia	−0.186_a_	0.050	0.017_b_	0.058

*Note*. Scores for each region were taken from the final measurement model with added deviation-coded region indicators and indicate how each region differs from the mean of the five regions. Means within each column that do not share a subscript differed significantly in post hoc pairwise comparisons using dummy-coded region indicators with Holm-Bonferroni adjustment. See Supplemental Table S24 for details of all pairwise comparisons. *Anglo-Western Heritage*: United States, United Kingdom, Canada; *Latin Europe*: Spain, Italy; *Southeast Europe*: Greece, Greek Cypriot community; *MENA*: Türkiye, Lebanon, Egypt, Tunisia, Turkish Cypriot Community; *East Asia*: South Korea, Japan.

## Study 3

Having established that the observed pattern of regional differences in honor values was not due to idiosyncrasies of student samples, we returned to the Study 1 data to test RQ4. We conducted a series of multilevel mediation models testing to what extent culture-level variation in perceived normative and personally endorsed honor values could explain previously reported differences in social cognitive tendencies between samples from Mediterranean societies and those from more commonly studied world regions (Uskul, Kirchner-Häusler, et al., 2023).

Uskul, Kirchner-Häusler, and colleagues (2023) proposed that a cultural logic of honor might account for four social cognitive tendencies observed in Mediterranean societies, compared to East Asian and/or Anglo-Western societies: (a) experiencing more socially disengaging emotions (e.g., pride, anger) relative to engaging emotions (e.g., closeness with others, shame), (b) linking happiness more closely to socially disengaging relative to engaging positive emotions, (c) viewing the self as more “inflated” relative to others, and (d) remembering events from a third-person perspective. They interpreted these tendencies as likely consequences of competing to maintain an “honorable” self in the unstable hierarchical contexts of societies where a cultural logic of honor prevails (e.g., Leung & Cohen, 2011; Stewart, 1994). Individuals living in such competitive environments may be expected to adapt by developing a clear sense of their personal importance and positive distinctiveness—hence, they might represent themselves symbolically as “larger” than others, and their emotional life might be especially focused on disengaged compared to engaged emotions. However, since judgments of honor are largely bestowed by others, individuals living in honor-based societies must also be closely attentive to how they appear to others—hence, they may develop a strong tendency to experience, and thus also remember, events from a third-person perspective.

Having found that honor values were perceived to be—and actually were—more prevalent in some Mediterranean societies than in others, we wanted to test to what extent culture-level variation in honor values would account for these previously reported trends in psychological tendencies among Mediterranean samples—potentially providing a more proximal and precise explanation than a simple contrast between geographical regions.

### Method

Study 1 participants also completed eight implicit measures resulting in 10 indicators of independent versus interdependent social orientation and analytical versus holistic cognitive style, summarized in Table 5, that were previously reported by Uskul, Kirchner-Häusler, and colleagues (2023).

**Table 5.** Implicit Measures of Social Cognitive Tendencies Used in the Mediational Analyses (RQ4).

Task	Measure	Operationalization/Assessment	Original interpretation
**Hypothesized outcomes (used in main analyses)**			
Implicit Social Orientation Questionnaire^a^	Experiencing Disengaging (vs. Engaging) Emotions	Intensity of socially disengaging emotions (e.g., ashamed) minus intensity of socially engaging emotions (e.g., proud)	Stronger relative intensity of socially disengaging (vs. engaging) emotions interpreted as social orientation toward independence (vs. interdependence)
	Personal (vs. Social) Happiness	Regression coefficient for socially disengaging emotions for happiness minus regression coefficient for socially engaging emotions	Stronger relative prediction of happiness by socially disengaging (vs. engaging) emotions interpreted as social orientation toward independence (vs. interdependence)
Sociogram Task	Symbolic Self-Inflation	Size of circle drawn for the self minus the average size of all circles drawn for others	Larger “self” (relative to others) interpreted as social orientation toward independence (vs. interdependence)
Outside-In Task	Third-Person Perspective Taking	Extent to which somebody took a third- versus a first-person perspective when remembering specific situations	Stronger tendency to take a third-person (vs. first-person) perspective interpreted as more holistic (vs. analytic) cognitive style
**Exploratory outcomes (used in supplementary analyses)**			
Inclusion of Other in the Self Scale (IOS)	Ingroup (vs. Outgroup) Closeness Bias	Average of felt closeness to ingroup members (the person they feel closest to, a good friend and family members) minus average of felt closeness to outgroup members (others in general, a stranger on the street)	Relatively greater closeness to ingroup (vs. outgroup) others interpreted as social orientation toward interdependence (vs. independence)
Nepotism Task	Nepotism in Reward Situations	The amount of money allocated to reward an *honest friend* minus the amount of money allocated to reward an *honest stranger*	Greater monetary reward of friends than strangers interpreted as social orientation toward interdependence (vs. independence)
	Nepotism in Punishment Situations	The amount of money allocated to punish a *dishonest stranger* minus the amount of money allocated to punish a *dishonest friend*	Greater monetary punishment of strangers than friends interpreted as social orientation toward interdependence (vs. independence)
Attribution Task	Causal Dispositional (vs. Situational) Attribution	Average across dispositional attribution items minus average across situational attribution items	Relatively greater attribution of causality to dispositional (vs. situational) factors interpreted as more analytic (vs. holistic) cognitive style
Triad Task	Thematic (vs. Taxonomic) Categorization	Percentage of items with thematic categorizations (based on their spatial, causal, or temporal relationships) out of all items	Relatively greater tendency to categorize objects in thematic terms interpreted as more holistic (vs. analytic) cognitive style
Inclusion Task	Inclusion of Contextual Information	Number of pieces of information perceived as relevant in resolving a murder case	Higher number of relevant pieces of information interpreted as more holistic (vs. analytic) cognitive style

a See Kirchner-Häusler et al. (2023) for evidence of scalar invariance of the Implicit Social Orientation Questionnaire in our current data.

#### Hypothesized Outcome Measures

Our main analyses focus on four measures: *experiencing disengaging (vs. engaging) emotions* and *personal (vs. social) happiness* obtained from the Implicit Social Orientation Questionnaire (Kitayama et al., 2009), *symbolic self-inflation* obtained from the sociogram task (Kitayama et al., 2009), and *third-person perspective taking* obtained from a memory task (Cohen & Gunz, 2002). Further details are in Table 5 (see also Uskul, Kirchner-Häusler, et al., 2023).

#### Additional Exploratory Outcome Measures

We used six further implicit measures in exploratory analyses (see Supplemental Materials): *ingroup closeness bias* obtained from the inclusion of the other in the self (IOS) task (Aron et al., 1992), *nepotism in reward situations* and *nepotism in punishment situations* obtained from the nepotism/loyalty task (Wang et al., 2011), *dispositional attribution bias* obtained from the attribution task (Kitayama et al., 2009), *thematic categorization* obtained from the triad task (L.-H. Chiu, 1972), and *inclusion of contextual information* obtained from a task describing a hypothetical murder case (Choi et al., 2003). Further details are in Table 5 (see also Uskul, Kirchner-Häusler, et al., 2023).

#### Analyses

We conducted eight multilevel mediation models with a 2-2-1 design (Preacher et al., 2010) using culture-level variation in either perceived normative or personally endorsed honor values to account for differences between Mediterranean (combining MENA, Southeast European and Latin-European samples) and non-Mediterranean regions (East Asian and Anglo-Western samples) in the four hypothesized outcome measures. We modeled between-samples variation in each outcome as a function of cultural region, coded with orthogonal contrasts, the first indicating category membership in Anglo-Western versus East Asian societies (coded: −0.5 = *East Asian societies*, 0.5 = *Anglo-Western societies*, 0 = *Mediterranean societies*), and the second—our focal contrast—indicating category membership in Mediterranean societies (coded: −0.5 = *East Asian societies*, −0.5 = *Anglo-Western societies*, 0.5 = *Mediterranean societies*); we included factor scores for perceived normative or personally endorsed honor values as a potential mediator explaining variation across the three regions in each outcome. Each model also included paths predicting within-sample variance in the outcome based on the two dimensions of individual-level variation in perceived normative or personally endorsed honor values (*defense of family reputation* and *self-promotion and retaliation*), controlling for age and SES (both uncentered).

Considering the small *N* (22 cultural groups) at the between-samples level, we kept these models as simple as possible. To avoid potential multicollinearity, we modeled differences across Mediterranean, East Asian, and Anglo-Western world regions—without attempting to differentiate zones within the Mediterranean region—and we focused narrowly on the theoretically relevant measures of perceived societal and personal honor values as potential mediators without attempting to include face or dignity values in parallel. Since perceived societal and personal honor values were strongly correlated (*r* = .654), we conducted separate models with each potential mediator, including the corresponding dimensions of individual-level variation in honor values at the within-samples level, yielding eight models in total: four with perceived normative honor values, four with personal honor values. Accordingly, we interpreted the significance of each pair of mediation effects (one for perceived normative honor values, one for personal honor values) using a Holm-Bonferroni sequentially adjusted alpha level beginning at 0.05 / 2 = .025 (Holm, 1979). Results are in Tables 6 and 7.

**Table 6.** Mediation Results for Cultural Differences in Social Cognitive Tendencies via Perceived Normative Honor Values.

Model parameters	Outcome															
	Experiencing disengaging (vs. engaging) emotions				Personal (vs. social) happiness				Symbolic self-inflation				Third-person perspective taking			
	*β*	*SE*	*p*	95% CI	*β*	*SE*	*p*	95% CI	*β*	*SE*	*p*	95% CI	*β*	*SE*	*p*	95% CI
**Between-samples parameters**																
Mediterranean contrast																
Region → Honor (path a)	.687	.081	<.001	.529, .845	.687	.081	<.001	.529, .845	.687	.081	<.001	.529, .845	.687	.081	<.001	.529, .845
Honor → Outcome (path b)	.616	.118	<.001	.384, .849	-.060	.165	.716	-.383, .263	-.317	.319	.319	-.942, .307	.729	.217	.001	.303, 1.154
Region → Outcome (path c’)	.258	.143	.072	-.023, .539	.833	.189	<.001	.346, 1.203	.801	.274	.003	.264, 1.338	-.313	.188	.096	-.681, .055
Total effect (path c)	.681	.115	<.001	.456, .906	.791	.120	<.001	.555, 1.028	.583	.222	.009	.147, 1.018	.188	.162	.246	-.130, .505
Indirect effect (a * b)	.423	.093	<.001*	.241, .606	-.041	.114	.718	-.265, .183	-.218	.223	.328	-.654, .218	.500	.165	.002*	.176, .825
West-East contrast																
Region → Honor (path a2)	.057	.076	.448	-.091, .206	.057	.076	.448	-.091, .206	.057	.076	.448	-.091, .206	.057	.076	.448	-.091, .206
Honor → Outcome (path b)	.616	.118	<.001	.384, .849	-.060	.165	.716	-.383, .263	-.317	.319	.319	-.942, .307	.729	.217	.001	.303, 1.154
Region → Outcome (path c2’)	.372	.124	.003	.129, .614	.578	.129	< .001	.324, .831	-.224	.130	.085	-.479, .031	-.512	.133	< .001	-.772, -.252
Total effect (path c2)	.407	.113	<.001	.185, .629	.574	.127	< .001	.325, .823	-.243	.116	.036	-.469, .016	-.470	.130	< .001	-.725, -.215
Indirect effect (a2 * b)	.035	.047	.453	-.057, .128	-.003	.011	.748	-.024, .018	-.018	.029	.523	-.074, .038	.042	.0.56	.454	-.068, .151
Modeled variance																
* R*^2^ (Honor)			47.5%				47.5%				47.5%				47.5%	
* R*^2^ (Outcome)			82.9%				95.8%				45.1%				53.5%	
**Within-samples parameters**																
Defense of Family Reputation → Outcome	-.005	.019	.805	-.042, .032	-.038	.017	.023	-.071, -.005	.005	.018	.780	-.030, .040	-.017	.020	.399	-.056, .022
Self-Promotion & Retaliation → Outcome	.030	.023	.186	-.015, .076	-.006	.019	.772	-.043, .032	-.015	.015	.333	-.044, .015	-.016	.016	.317	-.047, .015
Age → Outcome	-.037	.021	.076	-.077, .004	-.003	.024	.903	-.049, .044	-.037	.017	.033	-.072, -.003	-.015	.013	.234	-.041, .010
SES → Outcome	.028	.019	.144	-.010, .065	.021	.012	.085	-.003, .046	.052	.011	< .001	.029, .074	-.019	.013	.135	-.044, .006
Modeled variance																
* R*^2^ (Outcome)			0.3%				0.2%				0.5%				0.1%	

*Note*. Presented are the standardized estimates for models testing the role of culture-level perceived normative honor values as a mediator of regional differences in hypothesized outcomes. Models control for individual differences in two within-samples dimensions of perceived honor values, age, and SES. Cultural regions were coded using orthogonal contrasts for West-East category membership (0.5 = Anglo-West, −0.5 = East Asia, 0 = Mediterranean) and for Mediterranean category membership (−0.5 = Anglo-West, −0.5 = East Asia, 0.5 = Mediterranean). Indirect effects marked with an * meet a Holm-Bonferroni sequentially adjusted alpha level starting at .05 / 2 = .025, to adjust for familywise error in combination with the corresponding tests involving personally endorsed honor values in Table 7.

**Table 7.** Mediation Results for Cultural Differences in Social Cognitive Tendencies via Personal Honor Values.

Model parameters	Outcome															
	Experiencing disengaging (vs. engaging) emotions				Personal (vs. social) happiness				Symbolic self-inflation				Third-person perspective taking			
	*β*	*SE*	*p*	95% CI	*β*	*SE*	*p*	95% CI	*β*	*SE*	*p*	95% CI	*β*	*SE*	*p*	95% CI
**Between-samples parameters**																
Mediterranean contrast																
Region → Honor (path a)	.125	.160	.436	-.189, .438	.125	.160	.436	-.189, .438	.125	.160	.436	-.189, .438	.125	.160	.436	-.189, .438
Honor → Outcome (path b)	.453	.113	<.001	.232, .675	-.030	.090	.741	-.206, .147	-.422	.256	.100	-.924, .080	.548	.210	.009	.137, .959
Region → Outcome (path c’)	.633	.099	<.001	.439, .826	.794	.122	<.001	.555, 1.033	.633	.204	.002	.234, 1.033	.126	.156	.420	-.180, .431
Total effect (path c)	.689	.112	<.001	.469, .909	.790	.119	<.001	.218, .327	.581	.223	.009	.145, 1.017	.194	.166	.242	-.131, .519
Indirect effect (a * b)	.053	.065	.382	-.070, .183	-.004	.011	.745	-.009, .006	-.053	.064	.414	-.179, .074	.068	.094	.466	-.115, .252
West-East contrast																
Region → Honor (path a2)	-.105	.079	.184	-.261, .050	-.105	.079	.184	-.261, .050	-.105	.079	.184	-.261, .050	-.105	.079	.184	-.261, .050
Honor → Outcome (path b)	.453	.113	<.001	.232, .675	-.030	.090	.741	-.206, .147	-.422	.256	.100	-.924, .080	.548	.210	.009	.137, .959
Region → Outcome (path c2’)	.456	.110	<.001	.240, .673	.570	.126	<.001	.323, .817	-.298	.124	.016	-.542, -.055	-.412	.156	.008	-.718, -.106
Total effect (path c2)	.408	.113	<.001	.187, .630	.573	.126	<.001	.238, .393	-.254	.117	.030	-.483, -.025	-.469	.132	< .001	-.729, -.210
Indirect effect (a2 * b)	-.048	.038	.203	-.121, .026	.003	.010	.750	-.009, .012	.044	.045	.323	-.044, .133	-.058	.046	.208	-.148, .032
Modeled variance																
* R*^2^ (Honor)			2.7%				2.7%				2.7%				2.7%	
* R*^2^ (Outcome)			84.2%				95.4%				57.5%				55.0%	
**Within-samples parameters**																
Defense of Family Reputation → Outcome	.002	.031	.944	-.059, .064	.015	.015	.325	-.015, .045	.007	.022	.739	-.036, .051	-.072	.025	.004	-.121, -.023
Self-Promotion & Retaliation → Outcome	.168	.020	<.001	.130, .207	.034	.013	.011	.008, .060	-.031	.015	.033	-.060, -.003	.042	.020	.032	.004, .081
Age → Outcome	-.050	.021	.018	-.091, -.008	-.006	.023	.808	-.052, .040	-.038	.018	.040	-.074, -.002	-.018	.013	.165	-.043, .007
SES → Outcome	.025	.018	.177	-.011, .061	.017	.012	.175	-.007, .041	.053	.012	< .001	.029, .077	-.006	.012	.618	-.028, .017
Modeled variance																
* R*^2^ (Outcome)			3.1%				0.2%				0.5%				0.5%	

*Note*. Presented are the standardized estimates for models testing the role of culture-level personal honor values as a mediator of regional differences in hypothesized outcomes. Models control for individual differences in two within-samples dimensions of perceived honor values, age, and SES. Cultural regions were coded using orthogonal contrasts for West-East category membership (0.5 = Anglo-West, −0.5 = East Asia, 0 = Mediterranean) and for Mediterranean category membership (−0.5 = Anglo-West, −0.5 = East Asia, 0.5 = Mediterranean). Indirect effects marked with a * meet a Holm-Bonferroni sequentially adjusted alpha level starting at .05 / 2 = .025, to adjust for familywise error in combination with the corresponding tests involving perceived normative honor values in Table 6.

We conducted similar exploratory mediation models predicting the remaining six outcomes, on some of which Uskul, Kirchner-Häusler and colleagues (2023) had found significant differences between Mediterranean and non-Mediterranean samples but not linked these differences theoretically to honor. We interpreted the significance of mediation effects in these 12 models (six for perceived normative honor, six for personal honor) using Holm-Bonferroni corrections with a sequentially adjusted alpha level starting from .05/12 = .0042. Results are in Supplemental Tables S28 and S29. To guard against Type II error, we again interpreted any nonsignificant findings that reached *p* ≤ .05 as “marginal.”

### Results and Discussion

#### Perceived Normative Honor Values

**Figure 7.**
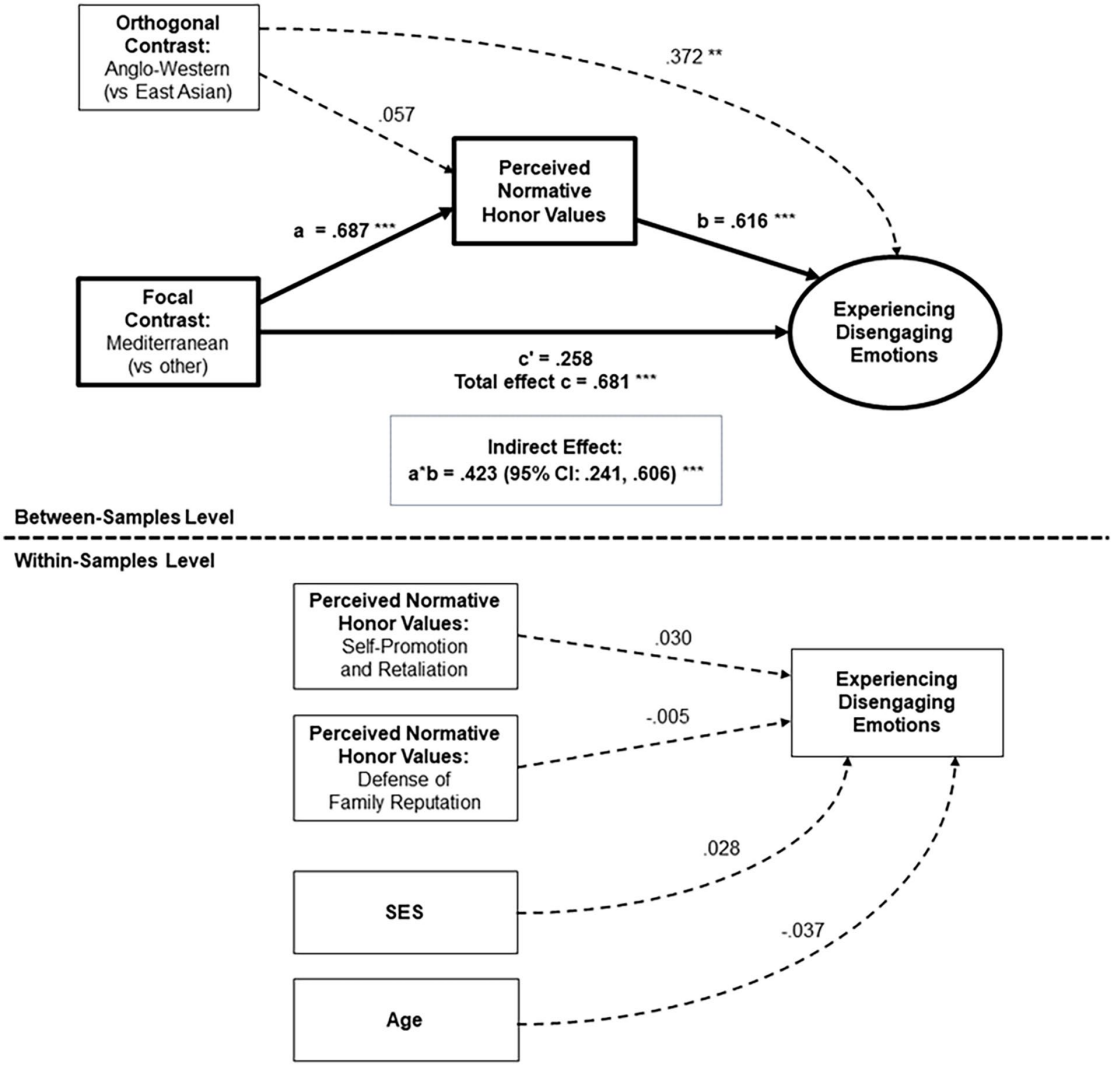
Mediation of Cultural Differences in Experiencing Disengaging (vs. Engaging) Emotions via Perceived Normative Honor Values. *Note*. The outcome of the mediation (Experiencing Disengaging Emotions) was coded so that higher values represent a greater tendency to experience disengaging (vs. engaging) emotions. *p < .05; **p < .01; ***p < .001.

**Figure 8.**
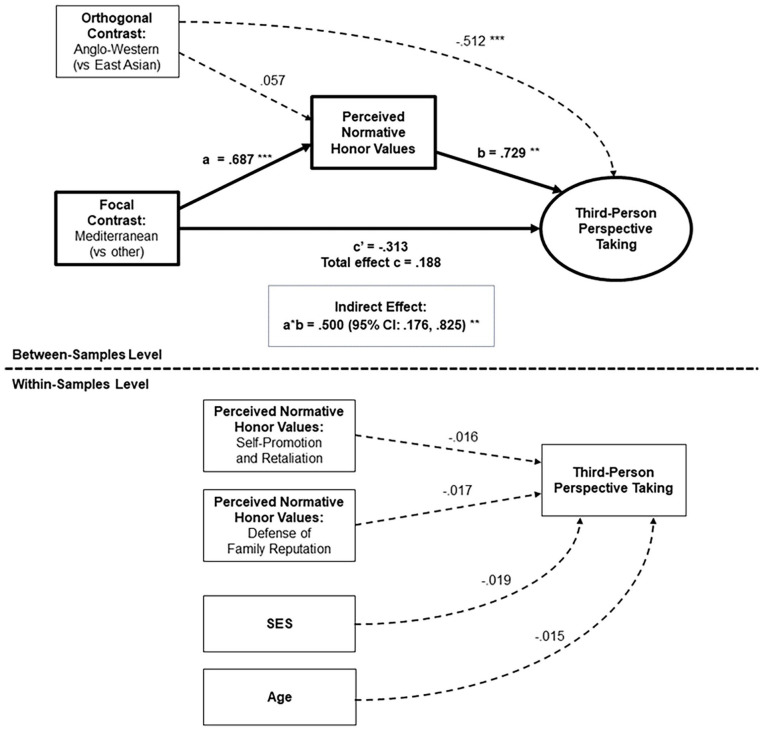
Mediation of Cultural Differences in Third-Person Perspective Taking via Perceived Normative Honor Values. *Note*. The outcome of the mediation (Third-Person Perspective Taking) was coded so that higher values represent a greater tendency to remember events from a third-person (vs. first-person) perspective. *p < .05; **p < .01; ***p < .001.

Perceived normative honor values accounted for differences between Mediterranean and non-Mediterranean cultural samples on two of the four predicted outcomes: *experiencing disengaging (vs. engaging) emotions*, and *third-person perspective taking* (Table 6). Figure 7 shows the significant indirect path from the focal contrast for Mediterranean cultures to experiencing disengaging (vs. engaging) emotions, through perceived normative honor values: Mediterranean cultural samples perceived higher prevalence of honor values in their societies (in all models: β = .687, *p* < .001). In turn, cultural samples with higher perceived normative honor values showed a greater tendency to experience disengaging versus engaging emotions (β = .616, *p* < .001), constituting a positive indirect effect: β = .423, *p* < .001 (95% CI: .241, .606). Figure 8 shows the significant indirect path from our focal contrast to third-person perspective taking. Here, cultural samples with higher perceived normative honor values showed a greater tendency to remember past situations from a third-person perspective (β = .729, *p* = .001), constituting a positive indirect effect: β = .500, *p* = .002 (95% CI: .176, .825). In both models, the direct effect from our focal contrast to the outcome was nonsignificant, consistent with full mediation.^5^

In contrast, we found no evidence to suggest that perceived normative honor values accounted for differences between Mediterranean and non-Mediterranean cultural samples in *personal (vs. social) happiness*, nor in *symbolic self-inflation*. Thus, perceived normative honor values seemingly provided an excellent explanation for the characteristics of Mediterranean cultural groups on two of the four predicted outcomes, but not the other two.

Exploratory analyses provided evidence for indirect paths through perceived normative honor values predicting two of the six additional outcomes: a significant path predicting lower nepotism in reward situations (i.e., a lower tendency to reward friends more than strangers for the same behavior) and a marginal path predicting dispositional (vs. situational) attribution bias (see Supplemental Materials and Table S28). We found no signs of mediation for the remaining exploratory outcomes.

#### Personal Honor Values

Table 7 shows results of our main mediation models involving personal honor values. Personal honor values were not significantly higher in Mediterranean cultural samples (β = 0.125, *p* = .436) and thus could not account for cultural differences between Mediterranean and non-Mediterranean societies in hypothesized or exploratory outcomes. Nonetheless, samples with higher personal honor values showed greater tendencies toward experiencing disengaging (vs. engaging) emotions and toward third-person memory perspective (see Table 7), as well as lower nepotism in reward situations (see Supplemental Table S29).

#### West-East Differences

Consistent with previous analyses of these data reported by Uskul, Kirchner-Häusler and colleagues (2023), total effects of the “West-East” contrast in both main and supplementary analyses, Tables 6 and 7, Supplemental Tables S28 and S29) showed differences across most outcomes, with Anglo-Western samples averaging a significantly more independent/analytic profile than East-Asian samples on 7 out of 10 tasks. As expected, we found no evidence to suggest that these West-East differences were explained by perceived normative honor values, nor by personal honor values (*p*s ≥ .180 for all 20 indirect effects tested). Thus, cultural variation in perceived normative honor values accounted for some of the distinctive characteristics of Mediterranean samples, but not for differences between Anglo-Western and East Asian regions.

#### Levels of Analysis

Within-sample paths reveal small and sometimes complex relationships between the two dimensions of honor values and selected outcome measures (Tables 6 and 7 and Supplemental Tables S28 and S29; for elaboration, see Supplemental Materials). Neither the pattern nor the magnitude of these paths suggested that culture-level associations of honor values with social cognitive tendencies could be reduced to an individual-level explanation. Thus, our findings seemingly reflect the social cognitive implications of living in a society where honor values are normatively perceived as prevalent—that is, where a certain “cultural logic” prevails—rather than being explicable as aggregated effects of individuals’ personal endorsement of honor values, nor even as effects of their individual perceptions of their cultural environments. This is consistent with viewing honor logic as a property of cultural groups, not individuals (Leung & Cohen, 2011).

## General Discussion

The tripartite distinction between cultural logics of honor, dignity, and face (Leung & Cohen, 2011) provides a theoretical framework for studying previously overlooked regions of the world, extending cultural psychology beyond its initial focus on East-West comparisons. Testing key assumptions of this framework, our findings map the prevalence of honor, dignity, and face values and concerns across Mediterranean, Anglo-Western, and East-Asian societies (RQ1); challenge the idea of uniform “honor cultures” by revealing regional differences among Mediterranean samples (RQ2); question assumptions about honor and gender by revealing more similarities than differences in endorsement of different honor facets (RQ3); and provide the first direct test of honor values in accounting for culture-level variations in social orientation and cognitive style (RQ4). Honor seemingly plays an important role in the cultural logics of Mediterranean societies—especially farther East and South within this region—but labeling societies of this region as “honor cultures” would be oversimplifying.

### Mapping Honor Values and Concerns Across the Mediterranean Region

Contrasting with the relative importance of dignity in Anglo-Western cultures, and face in East-Asian cultures, both university and general population samples from parts of the Mediterranean region showed a distinctive emphasis on honor-related values and concerns. This emphasis was stronger in samples located farther East and South within the Mediterranean region. *MENA* samples showed the highest perceived normativity and personal endorsement of most honor-related measures, especially for measures of general honor values and concerns about losing sexual propriety or family reputation.

Unexpectedly, student and general population samples from *Latin Europe*, a region traditionally described as an “honor” context (Peristiany, 1966; Rodriguez Mosquera et al., 2002a), consistently showed relatively low levels of honor endorsement, comparable to those of Anglo-Western samples. Previous studies similarly found lower-than-expected honor concerns or values in Spain (Guerra et al., 2013) and Italy (Helkama et al., 2013). These societies might have reduced their emphasis on honor since earlier characterizations; however, we did not find clear evidence of atypical age trends within Latin-European samples in Study 2 (see Supplemental Table S27), which would be expected if there were a recent generational shift specific to this cultural region. Alternatively, the seeming discrepancy with earlier findings could be due to differing frames of comparison used in different studies. Classic studies found higher honor values or concerns in Spain than in the Netherlands (Fischer et al., 1999; Rodriguez Mosquera et al., 2002a, 2002b). Notably, the Netherlands scores considerably higher than Anglo-Western countries in cultural individualism (Minkov & Kaasa, 2022)—which is antithetical to a cultural logic of honor. Consistently, Smith and colleagues (2021) found that participants from the Netherlands showed the highest perceived normative dignity values and the lowest perceived normative honor values among 29 samples from 24 different countries, whereas the UK showed a milder profile, and two US samples showed a moderate focus on honor similar to the profile observed in Italy. Thus, it is possible that Latin-European societies may have a greater culture of honor than Northern European societies, such as the Netherlands and Scandinavian countries, but not compared to Anglo-Western societies.

Even where mean endorsement is low, individual differences in honor values or concerns may still play an important role in psychological functioning. Individual differences in honor endorsement have shown meaningful associations with relevant outcomes in many different societal contexts (e.g., Finland: Helkama et al., 2013; Netherlands: IJzerman & Cohen, 2011; southern Italy: Travaglino et al., 2015, 2024; Lebanon and Syria: Levin et al., 2015; UAE: Maitner et al., 2022; Canada: Mandel & Litt, 2012), even where expected cross-cultural mean differences were not found (Cross et al., 2014; Uskul et al., 2015). Thus, the presence or absence of culture-level variation does not capture the whole story about the role of honor in predicting or shaping individuals’ psychological processes.

Cultural groups from *Southeast European* societies showed a more complex pattern, typically perceiving honor values, as well as concerns about losing family reputation, family authority, and sexual propriety, as moderately prevalent in their societies, but showing relatively low personal endorsement of honor values and of sexual propriety concerns. Thus, individuals seemingly perceived that other society members would rate honor values and concerns as more important than they actually did. This pattern suggests that honor values and concerns currently may be contested in these societies—perhaps signaling historical change. Notably, samples from this region showed the strongest age trends in personal values of defending family reputation, implying that there may be a greater generational divide around honor values in these societies than in some others (see Supplemental Materials and Table S27). Future research should track historical changes in perceived and personally endorsed honor, face, and dignity values and concerns over forthcoming decades in these societies.

Although prevalence of honor values and concerns did not map straightforwardly onto a distinction between Mediterranean and non-Mediterranean cultures, perceived normative honor values provided a statistically convincing explanation for certain previously observed differences in social cognitive tendencies between Mediterranean and non-Mediterranean samples. Perceived normative honor values accounted for the tendency of Mediterranean samples to report stronger disengaging (vs. engaging) emotions, as well as more third-person (vs. first-person) memories, compared to samples from East-Asian and/or Anglo-Western regions. This combination of social cognitive tendencies would be hard to interpret based on classic independence-interdependence theory, which was initially developed to compare cultures with greater prevalence of dignity and face logics (e.g., Kitayama et al., 2009). However, the same combination makes good sense within an honor-based cultural system—where individuals are expected to compete for social standing in unstable hierarchies (hence, emotional disengagement from others), but their social standing is highly contingent on how they are perceived by others (hence, the need to monitor one’s social image through a third-person perspective). These two outcomes showed findings consistent with full mediation by perceived normative honor values.

Conversely, honor values explained almost no variance in tendencies for disengaging emotions to be more closely linked to happiness, nor for greater symbolic self-inflation, where Mediterranean cultural groups had also shown distinctive cultural emphases (Uskul, Kirchner-Häusler, et al., 2023). Thus, in addition to being distributed unevenly across the Mediterranean societies studied here, honor values accounted for only some of the social cognitive tendencies with proposed theoretical links to honor. This suggests caution in viewing the Mediterranean region as a homogeneous “honor context” and underscores the need for a more fine-grained geographical and conceptual approach to describing and studying cultures.

### Recognizing the Complexity of Cultural Logics

Cultural differences did not emerge equally in all dimensions of honor, showing the importance of considering honor as multifaceted (Rodriguez Mosquera, 2016). For example, perceived normative concerns about losing family reputation or family authority were stronger in MENA and Southeast European samples than in Anglo-Western and Latin-European samples, whereas we found no such differences in concerns about losing integrity. Future research should consider honor logic as a multidimensional construct (e.g., Rodriguez Mosquera, 2016), rather than a cultural “type” (e.g., Leung & Cohen, 2011). Honor values and concerns may be measured on different levels of analysis (between societies, within societies), from different perspectives (perceived norms, personal endorsement), and in multiple domains (valuing honor in general, concerns about specific ways of losing honor).

Our findings caution against using “clear-cut” categories to describe cultural variation. Supposed “dignity,” “face,” and “honor” cultures did not form perfect clusters but showed similarities and differences that were not neatly captured by the tripartite model of cultural logics. East Asian and MENA samples showed the highest personal endorsement of certain “honor concerns,” such as concerns about losing sexual propriety or family reputation; these concerns may play a similarly important role in a logic of face, where in-groups are highly important, and self-restraint is expected. Southeast European samples showed higher perceived normative and personal concerns about losing dignity than “dignity-focused” Anglo-Western samples; perhaps Anglo-Western samples may be less concerned about losing dignity, as dignity is thought to be inherent and inalienable within these societies (Leung & Cohen, 2011). MENA samples showed higher personal endorsement of face values than East Asian samples and perceived their societies to endorse face relatively highly, perhaps suggesting a shared emphasis on social harmony (Boiger et al., 2014).

### Cultural Logics as Properties of Societies, Not Individuals

Cultural differences in perceived normative values and concerns were more pronounced than the corresponding differences in personal endorsement. This highlights the value of treating research participants as *informants* about the cultural contexts they inhabit, not solely as *exemplars* of cultural groups. Research into “intersubjective culture” has shown that individuals’ perceptions of social norms or of others’ attitudes can sometimes play a stronger role than their own beliefs or attitudes in predicting behavior (C.-Y. Chiu et al., 2010). Here, in a multilevel extension of those findings, *culture-level* variation in perceived normative values predicted social cognitive tendencies more reliably than did individual differences in perceived or personally endorsed values (cf. Table 6 and Supplemental Table S28 vs. Table 7 and Supplemental Table S29). Conceptualizing honor, face, and dignity as “cultural logics” entails that ways of thinking and acting may be influenced by developing and living in a context where a certain cultural logic is prevalent, whether or not one personally agrees with the corresponding values and concerns (Leung & Cohen, 2011). Indeed, social cognitive tendencies—such as one’s repertoire of emotional responses to situations or the tendency to view oneself from others’ perspectives—plausibly might develop much earlier in life than endorsement of abstract cultural ideas such as “honor” or “interdependence” (Kitayama & Uskul, 2011). These tendencies may be closely linked to practices, habits, and worldviews that are tacit and unexamined, and thus are not easily predicted by personal beliefs and values. Future research should continue to disentangle the effects of living in a certain (perceived) cultural context from the effects of personally endorsing or internalizing the prevailing cultural beliefs, values, or concerns of a certain cultural group (Na et al., 2010).

### Honor and Gender

At odds with conceptualizing honor as a highly gendered construct, we found almost no gender differences: samples of women and men in each society endorsed perceived normative and personal values and concerns at remarkably similar levels. One sole exception was that young women tended to view concerns about losing sexual propriety as more prevalent in their society than did young men; however, the corresponding gender difference in personal concerns about losing sexual propriety did not approach significance. These findings only partially support the conceptualization of sexual propriety concerns as “feminine honor” in previous research (Guerra et al., 2013; Rodriguez Mosquera et al., 2002b). Against conceptualizations of “masculine honor,” concerns about losing family authority were not significantly higher among young men than among young women. Nonetheless, future research should test for gender differences among participants with more varying background characteristics.

## Conclusion

Our research contributes to globalizing psychological knowledge by mapping the prevalence of honor, face, and dignity values and concerns among cultural samples from three major world regions. Our findings provide conceptual and methodological insights into the structure and measurement of cultural logics as distinct, but multifaceted, constructs—comprising systematic variation in relevant values and concerns at a cultural, rather than individual, level of analysis—and illustrate how culture-level variation in these logics can be used to explain cross-cultural differences in psychological tendencies. Future research should extend our approach to a wider range of global regions and explore how cultural variations in dignity, face, and honor logics relate to the cultural dimensions measured in other theoretical frameworks (Minkov & Kaasa, 2022; Schwartz, 2006; Vignoles et al., 2016).

## Supplemental Material

sj-docx-1-psp-10.1177_01461672241295500 – Supplemental material for Are Mediterranean Societies “Cultures of Honor?”: Prevalence and Implications of a Cultural Logic of Honor Across Three World Regions Supplemental material, sj-docx-1-psp-10.1177_01461672241295500 for Are Mediterranean Societies “Cultures of Honor?”: Prevalence and Implications of a Cultural Logic of Honor Across Three World Regions by Vivian L. Vignoles, Alexander Kirchner-Häusler, Ayse K. Uskul, Susan E. Cross, Rosa Rodriguez-Bailón, Isabella R. L. Bossom, Vanessa A. Castillo, Meral Gezici-Yalçın, Charles Harb, Keiko Ishii, Panagiota Karamaouna, Konstantinos Kafetsios, Evangelia Kateri, Juan Matamoros-Lima, Rania Miniesy, Jinkyung Na, Zafer Özkan, Stefano Pagliaro, Charis Psaltis, Dina Rabie, Manuel Teresi, Yukiko Uchida and Michael J. A. Wohl in Personality and Social Psychology Bulletin

## Notes

1. Reflecting this diversity, our samples varied in religious background (Catholic, Orthodox, Muslim), dominant language (Spanish, Italian, Greek, Turkish, Arabic), and geographic/socio-political region (Southern Europe, Southeast Europe, MENA), among other characteristics. Thus, we treated the Mediterranean region as a *context*, rather than an *object* (Albera, 2006), and we compare groups from this context to those from other commonly studied cultural contexts, rather than seeking to provide a simplified narrative about a “Mediterranean identity or cultural area.” We aimed to capture complexity and diversity across the region, as much as to find commonalities.

2. Study 1 data for honor values and concerns in Mediterranean societies were previously reported by Kirchner-Häusler and colleagues (2024); Study 1 data for dignity, face, and honor values in the Greek Cypriot community were previously reported by Psaltis and colleagues (2023). Both previous papers addressed different RQs from the current paper. Study 1 data for East Asian and Anglo-Western samples, as well as dignity and face measures for all Mediterranean samples except for the Greek Cypriot community, have not previously been reported.

3. We shifted from lab-based to online data-collection setting due to COVID-related restrictions. Hence, we could gather data only from participants who were digitally connected with access to a suitable device. Although this limitation applies to most studies conducted during the pandemic, we acknowledge that this may have influenced our findings.

4. Family authority items were labeled “masculine honor,” and sexual propriety items were labeled “feminine honor” in previous research (Guerra et al., 2013). We prefer gender-neutral labels for these factors as we wanted to test, rather than prejudge, their gender distributions.

5. When predicting third-person perspective taking, the total effect (path *c*) of the Mediterranean contrast was nonsignificant, and the direct effect (path *c’*) was marginally negative (Table 6; Figure 8). In the analyses by Uskul, Kirchner-Häusler, and colleagues (2023), third-person perspective-taking was distinctively high in Mediterranean samples compared to that in Anglo-Western samples, but not compared to that in East-Asian samples, explaining the nonsignificant total effect when Mediterranean samples were contrasted here against both other regions together. The negative sign on the direct effect, even if nonsignificant, suggests a possible suppression effect, such that Mediterranean samples would be, if anything, less likely than samples from the other two regions to adopt a third-person memory perspective, if it were not for their higher perceived normative honor values. One possible explanation for this may be the relatively low perceived normative face values among Mediterranean samples, whereas East Asian samples showed relatively high perceived normative face values (Figure 1) and comparable third-person perspective taking to Mediterranean samples (Uskul, Kirchner-Häusler et al., 2023). Theoretically, face logic would be expected to predict third-person perspective taking. Future research should investigate this possibility.
