# Supplementary material for: Are Mediterranean Societies “Cultures of Honor?”: Prevalence and Implications of a Cultural Logic of Honor Across Three World Regions
Source: Pers Soc Psychol Bull. 2024 Dec 30;52(5):1157–83. doi: 10.1177/01461672241295500 (PMC13022031; doi:10.1177/01461672241295500)
Supplement: sj-docx-1-psp-10.1177_01461672241295500 – Supplemental material for Are Mediterranean Societies “Cultures of Honor?”: Prevalence and Implications of a Cultural Logic of Honor Across Three World Regions [file sj-docx-1-psp-10.1177_01461672241295500.docx]

# Supplementary Materials for:

**Are Mediterranean Societies ‘Cultures of Honor’? Prevalence and Implications of a Cultural Logic of Honor Across Three World Regions**

**Other supplementary materials for this manuscript include the following:**

The final materials, dataset, and analysis scripts and output are available on the Open Science Framework (<https://osf.io/wx5eh/?view_only=02a447f2bf9c422689d04b0207c647f4>).

# Overview of Supplementary Materials

Here, we report information regarding the (1) measurement models (RQ1 to RQ3) testing the item-level structure for measures of dignity, face and honor values and concerns in Study 1 (using student samples) and Study 2 (using general population samples), (2) further details of our analyses testing pairwise differences across cultural regions (Study 1 and Study 2), (3) full details of the Method and Results of Study 2, and (4) the honor mediation models (RQ4) for social orientation and cognitive style (Study 3: further analysis of Study 1 data).

# Study 1: Supplementary Information

We conducted measurement models to establish the factor structure of our values and concerns items at the individual and sample level, and to compare the different regions in terms of their value and concern endorsement. We conducted parallel series of measurement models for each set of items (i.e., perceived normative values, perceived normative concerns, personal values, personal concerns), accounting for the multi-level structure of the data (nesting participants within cultural samples defined by the intersection of country and gender) and for differences in response styles. Where mentioned, we used factor scores derived from the final measurement models. We also report full details of our tests for pairwise differences across five cultural regions, which are summarized in the main text.

## Measurement Models

### Model Selection Procedure

Analyses were conducted using Mplus Version 8.5 (Muthén & Muthén, 1998). We evaluated model fit using the Comparative Fit Index (CFI), Tucker-Lewis Index (TLI), Root Mean Square Error of Approximation (RMSEA), and Standardized Root Mean Squared Residual (SRMR). Values of CFI and TLI > .95 (or > .90) RMSEA < .06 (or < .08), and SRMR < .08 (or < .10) have been proposed as criteria for “good” (or “acceptable”) fit (Hu & Bentler, 1999; Kline, 2023). Note that all these cut-off values should be considered as “rules of thumb” to aid interpretation and not used to draw firm inferences. They are mostly based on simpler statistical models tested in single groups only, and so they may be excessively stringent for more complex scenarios such as our current goal of evaluating a multi-factor instrument across 22 culturally diverse samples (Marsh et al., 2004). For multilevel models, Mplus provides separate values of SRMR for the within-samples and between-samples parts of the model: SRMR_within_ and SRMR_between_. However, it is known that SRMR becomes inflated and is arguably of limited use with sample sizes below 200 (Asparouhov & Muthén, 2018). With 22 units of analysis at the between-samples level of our multilevel models, we therefore considered that values of SRMR_between_ < .20 should be considered acceptable, provided that other fit indices did not suggest otherwise.

For each of the four sets of items (i.e., perceived normative values, perceived normative concerns, personal values, personal concerns), we conducted our model selection in four sequential steps:

First, we conducted an ***exploratory factor analysis (EFA)*** on items assessing honor values or concerns to determine the most meaningful structure in these items at the individual level of analysis, using the TYPE=COMPLEX function in MPLUS to account for clustering of individuals into 22 cultural samples. For honor concerns, we expected to find a four-dimensional structure based on previous research (Guerra et al., 2013). For honor values, items were compiled from two different scales, and so we wanted to establish whether they formed a single factor or multiple factors in their own terms, before including them in a measurement model together with items measuring dignity and face values.

Second, we conducted a ***confirmatory factor analysis (CFA)*** using all honor, face, and dignity items, modeling the structure for honor items identified in the first step together with single factors for dignity and face, again using the TYPE=COMPLEX function to account for clustering. We included an additional method factor in the CFA, to model participants’ response styles (loadings of all items on this factor were set to 1, and the factor itself was uncorrelated with all substantive factors; see Welkenhuysen-Gybels et al., 2003). For models of perceived normative and personal values, this method factor captures differences in acquiescent responding—i.e., the extent to which participants tended to agree or disagree in general with the value items *irrespective of their content* (or the extent to which they perceived that others in their society would do so). Correspondingly, the method factor for perceived normative or personal concerns captures differences in the extent to which participants tended to think they would feel bad about themselves following untoward actions in general, *irrespective of which norms were described as being violated in different sets of items* (or the extent to which they perceived that others in their society would do so). Allowing items to cross-load on these method factors ensured that the substantive factors in our models were focused on the specific content of dignity, face, and honor values and concerns portrayed in the respective sets of items, as opposed to general tendencies to agree with things or to feel bad about things. Depending on the fit of the CFA, we screened the fitted solution for necessary changes in the item structure (i.e., based on suggested modification indices and/or low item loadings, combined with theoretical considerations on the meaning of the items).

Third, we tested the within-samples CFA structure for metric invariance across cultural regions and across genders using ***multigroup invariance testing***. To test invariance across regions, we grouped our 22 samples into 5 cultural regions that had been distinguished by previous research based on socio-demographic, religious, linguistic, and historical dimensions: These regions were Anglo-Western (UK, US), Latin Europe (Spain, Italy), Southeastern Europe (Greece, Cyprus: Greek Cypriot Community), MENA (Turkey, Lebanon, Egypt), and East Asia (Japan, South Korea) (Mensah & Chen, 2012). We ran these models as two-level models with an empty between-samples level; all items were centered within samples and the structure was modelled at the within-samples level only. In line with previous testing approaches, we compared a ***constrained model***, in which the loadings of all items are constrained to be equal across all comparison groups, against an ***unconstrained model***, in which item loadings were allowed to vary across all comparison groups. We adopted the conventional rule that it was tenable to assume invariance if ΔCFI ≤ .01 when comparing constrained and unconstrained models (Cheung & Rensvold, 2002). Where this criterion was not met, we considered items as potentially non-invariant and eligible for exclusion if the item showed both (a) a modification index > 10 in the constrained model that suggested freeing up the loading constraint across groups (thus implying that the size of the loading differed significantly across groups) and (b) one or more non-significant loadings in the unconstrained model (suggesting the item may not be a valid indicator of its target factor in one or more groups).

In a final step, we tested whether the identified within-samples structure would hold in the same way at the between-samples level of analysis (*N* = 22 samples), by using a ***multilevel confirmatory factor analysis*** to separately model our factors at the within- and between-samples levels. In this step, we always started with a constrained model that set the factor structure and loadings to be equal at both levels of analysis, and thus first followed a between-samples level factor structure that was the same as the existing within-samples model. We then explored various unconstrained models in which the factor structure and loadings at the between-samples level were allowed to differ from the established structure and loadings at the within-samples level. We then refined the model structure at the between-samples level, based on (a) how well the between-samples model fit the data overall, and (b) the strength of individual item loadings (i.e., removing non-significant items from the sample-level structure), until a final model had been reached that was theoretically meaningful and fit the data well.

### Perceived Normative Values

As our items for perceived normative honor values were combined from different scales, we first conducted an **exploratory factor analysis** to identify the underlying structure for these items at the individual level. Items were uncentered and clustered by the 22 samples made up by the combination of gender (male vs female) and country. A **two-factor solution** appeared to be the most theoretically meaningful and parsimonious solution. We deemed a one-factor solution as less theoretically informative than a two-factor solution. A four-factor solution included a single item factor, and was therefore considered to be overfitted, while a five-factor solution did not converge. In a three-factor solution, the items of an additional third factor showed relatively high primary loadings, but also introduced relatively higher cross-loadings to other factors; we therefore chose to proceed with a two-factor solution, (which mirrored the structure found for personal endorsement values, described below). Fit of the final two-factor solution was excellent (*χ^2^_[26]_* = 136.556, *CFI* = .991, *TLI* = .984, *SRMR* = .018, *RMSEA* = .038). As shown in Table S1, primary loadings of items were high (all above 0.4), with cross-loadings being below 0.3 for all items. Based on the pattern of item loadings, we interpreted the first factor as measuring *defense of family reputation* and the second factor as measuring *self-promotion and retaliation.*

In the second step, we conducted a **confirmatory factor analysis** of all value items with four substantial factors (i.e., dignity, face, defense of family reputation, and self-promotion & retaliation) and a method factor to account for differences in response style (see Table S2 for primary loadings on substantive factors). Fit of the model was good (*χ^2^_[202]_* = 911.192, *CFI* = .957, *TLI* = .950, *SRMR* = .043, *RMSEA* = .035), and loadings for all items were significant. Modification indices suggested some possible cross-loadings (highest χ^2^ change = 76.4); however, the suggested additional cross-loadings for items were generally low in strength (< .209 in absolute terms) and the respective primary loadings of items were at least twice as large in all cases. Given that the model already showed uniformly good fit indices, we therefore decided to retain all items for further analyses and not add any cross-loadings to the model in this step.

In the third step, we conducted **multigroup invariance testing** with the established four-factor structure. We tested invariance both across cultural regions, and across genders (female and male). An unconstrained model fit slightly better than a constrained model across cultural regions (Constrained: *χ^2^_[1192]_* = 2958.199, *CFI* = .908, *TLI* = .911, *SRMR* = .034, *RMSEA* = .050; Unconstrained: *χ^2^_[1120]_* = 2670.976, *CFI* = .920, *TLI* = .917, *SRMR* = .031, *RMSEA* = .049; *ΔCFI* = .012), but not across gender groups (Constrained: *χ^2^_[466]_* = 1216.545, *CFI* = .947, *TLI* = .947, *SRMR* = .033, *RMSEA* = .033; Unconstrained: *χ^2^_[448]_* = 1221.264, *CFI* = .945, *TLI* = .943, *SRMR* = .032, *RMSEA* = .034; *ΔCFI* = .002). We thus assumed invariance of our items across genders, but we followed up our invariance analysis on an item-by-item basis across regions. Two items failed our test of invariance across regions (“*People must always be ready to defend their honor*” and “*It is important to promote oneself to others*”). Both items had substantial modification indices in the constrained model (both χ^2^ change > 10.85) and also showed at least one non-significant loading in the unconstrained model, indicating that they performed poorly in certain regions. We therefore excluded both items from further analysis (and, in parallel, we also excluded these items from the models for personally endorsed values, see section below).

Finally, we conducted a **multilevel confirmatory factor analysis** to model the factor structure of our data at the between-samples level. To this end, we first added a between-samples factor structure that mirrored our existing within-samples four-factor model; we tested models with loadings constrained across levels (i.e., complete isomorphism) or freely varying across levels (i.e., configural isomorphism). However, in both models we found that the two dimensions of honor (defense of family reputation, and self-promotion & retaliation) were almost perfectly correlated at the between-samples level (*r* = .982). Hence, we merged these two dimensions, resulting in a three-dimensional structure at the between-samples level comprising dignity, face, and honor factors. Following some minor modifications, described below, we found that a three-factor model on the between-samples level (see Table S3) was the most theoretically meaningful, interpretable, and fit the data well (*χ^2^_[293]_* = 1232.323, *CFI* = .948, *TLI* = .939, *RMSEA* = .033, *SRMR_Within_* = .040, *SRMR_Between_* = .118). Two items measuring face values (“*People should minimize conflict in social relationships at all costs.*” and “*People should never criticize others in public.*”) were retained only at the within, but not at the between-samples level due to non-significant loadings; these items were therefore centered within samples. Furthermore, we included two cross-loadings, allowing two items of the dignity factor (“*People should make decisions based on their own opinions and not based on what others think.*” and “*People should be true to themselves regardless of what others think.*”) to load negatively on the honor factor
(-.644 and -.471, respectively). We included these cross-loadings (a) since including them lead to a substantial improvement, and best fit in general, of the between-samples level model fit (as highlighted by the *SRMR*_between_ = .118 of the final model compared to a model not including the cross-loadings, *SRMR*_between_ =.200), and (b) after considering the meaning of the two items. We considered that both items highlighted autonomy and individual characteristics disconnected from others (“*[...] not based on / regardless of what others think*”) that go against a concern for the opinion of others that is characteristic of honor (aligning with the negative suggested loadings). We decided it was most appropriate to allow these cross-loadings, rather than excluding the items in question, so that the information from these items would not be lost from our factor scores for the main analysis, whilst the measurement of each factor would be appropriately adjusted to reflect these additional loadings. (Nevertheless, the resulting factor scores were highly similar regardless of how these items were treated, *min*_r_ = .966.) All factors at the between-samples level showed significant variances in the final model.

### Perceived Normative Concerns

In line with our approach for values, in a first step we conducted an **exploratory factor analysis** to examine the underlying item structure at the individual level. Items were uncentered and clustered by the 22 samples. Our honor items were taken from Guerra and colleagues' (Guerra et al., 2013) short version of the Honor Scale originally developed by Mosquera and colleagues (Rodriguez Mosquera et al., 2002). However, we mistakenly included one item from the original scale *“...your sister or mother had the reputation of sleeping around*” in place of an item from the short version (“*You were unable to defend your family’s reputation*”); since this item was part of the same subscale as the correct item in the original version of the Honor Scale we decided not to exclude it and to proceed with all items. Consistent with the original scale, a **four-factor solution** appeared to be the most theoretically meaningful solution. We deemed a three-factor solution as less theoretically informative than a two-factor solution, as the obtained factors showed higher cross-loadings and thus were more strongly confounded; fit of the four-factor solution (*χ^2^_[62]_* = 188.792, *CFI* = .991, *TLI* = .983, *RMSEA* = .026, *SRMR* = .012) was also substantially better than that of a three-factor solution (*χ^2^_[75]_* = 1702.361, *CFI* = .890, *TLI* = .824, *RMSEA* = .086, *SRMR* = .039). A five-factor solution as well as a six-factor solution both included a single item factor and were therefore considered to be overfitted. The four factors that emerged mirrored those found by Guerra and colleagues (2013), and all except two items showed high primary loadings (above 0.47) and low cross-loadings (below 0.3) on the expected factors, as shown in Table S4. One item (“*...you were known as someone who cannot support a family?*”) loaded unexpectedly on the second rather than the first factor, and one item (“*...you had the reputation of being someone without sexual experience?*”) loaded weakly on all factors; we therefore excluded these items from further analyses, leaving us with a total of 14 honor items (the same as for the models with personal endorsement honor concerns, described in section below). We interpreted the four factors as reflecting concerns about losing *family authority*, *family reputation*, *integrity*, and *sexual propriety*. Items measuring family authority have been labelled “masculine honor” and items measuring sexual propriety have been labelled “feminine honor” in previous research (e.g., Guerra et al., 2013). We prefer gender-neutral descriptive labels for these factors as we wanted to test, rather than prejudge, their gender distribution.

In the second step, we conducted a **confirmatory factor analysis** of all concern items with six substantial factors (i.e., dignity, face, family reputation, sexual propriety, family authority, and integrity), as well as a method factor to account for differences in response style (see Table S5 for primary loadings on substantive factors). Fit of the model was very good (*χ^2^_[193]_* = 827.099, *CFI* = .972, *TLI* = .966, *SRMR* = .042, *RMSEA* = .033). The loadings for all items were significant. Cross-loadings as suggested by the modification indices for most items were low in absolute strength (≤ 0.229) and less than half their respective primary loadings, except for the item “*...your sister or mother had the reputation of sleeping around?*” of the family reputation factor with suggested cross-loading on integrity (0.118) and family authority (-0.093), and the item “*...you let other people insult your family?*” of the family reputation factor with suggested cross-loadings on family authority (-0.077), sexual propriety (-0.154), integrity (-0.141), and face (-0.209). However, since the (a) model fit was already very good, b) the EFA had showed these items to load higher on their target factor, and as highly as other items of the factor, and c) the suggested cross-loadings were still all rather low in absolute strength, we decided not to exclude any items at this step or add any cross-loadings.

In the third step, we then conducted **multigroup invariance testing** with the established six-factor structure. We tested invariance both across cultural regions, as well as genders (female and male). An unconstrained model did not fit better than a constrained model across cultural regions (Constrained: *χ^2^_[1139]_* = 2985.389, *CFI* = .930, *TLI* = .929, *SRMR* = .034, *RMSEA* = .053; Unconstrained: *χ^2^_[1075]_* = 2834.901, *CFI* = .933, *TLI* = .928, *SRMR* = .033, *RMSEA* = .053; *ΔCFI* = .003) and across genders (Constrained: *χ^2^_[446]_* = 1051.907, *CFI* = .936, *TLI* = .933, *SRMR* = .050, *RMSEA* = .055; Unconstrained: *χ^2^_[430]_* = 891.888, *CFI* = .941, *TLI* = .937, *SRMR* = .047, *RMSEA* = .053; *ΔCFI* = .005). We thus assumed metric invariance across genders as well as across regions.

In a final step, we used **multilevel confirmatory factor analysis** to model the between-samples factor structure of our data. To this end, we added a between-samples factor structure that mirrored our existing within-samples six-factor model. We started with a constrained model that set the factor structure and loadings to be equal at both levels of analysis, before exploring various unconstrained models in which the factor structure and loadings at the between-samples level were allowed to differ from the established structure and loadings at the within-samples level. We found that a model with all six factors (i.e., dignity, face, family reputation, family authority, integrity, and sexual propriety) on the between-samples level was most theoretically meaningful, interpretable, and best fitting (for loadings on substantial factors of this final model, see Table S6). The model fit the data well (*χ^2^_[336]_* = 1573.452, *CFI* = .953, *TLI* = .944, *RMSEA* = .035, *SRMR_Within_* = .042, *SRMR_Between_* = .133). The four-factor structure mirrored the factor structure found on the within-samples level, with some slight differences: We restricted one item from the dignity factor (“*...you did not stand up for what you believe?*”), one item from the face factor (“*...you lost control over your behavior in front of others?*”), and one item from the integrity factor (“*...you had the reputation of being dishonest with others?*”) to the within-samples level only due to non-significant loadings at the between-samples level; these items were therefore centered within samples. All factors at the between-samples level showed significant variances in the final model. Modification indices for the final model suggested a cross-loading for one item of the sexual propriety factor (“*...you were known as someone whom it is easy to sleep with?”)* on the family reputation factor, however the suggested strength of the loading was relatively low (0.296) compared to the primary loading of the item (0.944), and so we decided not to include it in the final model.

### Personal Values

As our items for personal honor values were combined from different scales, we conducted an **exploratory factor analysis** to identify the underlying item structure at the individual level. Items were uncentered and clustered by the 22 samples. A two-factor solution appeared to be the most theoretically meaningful and parsimonious solution. We deemed a one-factor solution as less theoretically informative, and the items of an additional third factor in a three-factor solution showed relatively low primary loadings and high cross-loadings across factors. The same was true for a four-factor solution. A five-factor solution included a single item factor and was therefore considered to be overfitted. Fit of the final two-factor solution was good (*χ^2^_[26]_* = 314.138, *CFI* = .977, *TLI* = .959, *SRMR* = .026, *RMSEA* = .061). As shown in Table S7, primary standardized loadings of all items were high (above 0.4), with cross-loadings being below 0.3 for all but one item (0.314 for the item “*People must always be ready to defend their honor.*”). Similar to the analyses for perceived normative honor values, the two factors that emerged were interpreted as measuring *defense of family reputation* and *self-promotion and retaliation.*

In the second step, we conducted a **confirmatory factor analysis** of all value items with four substantial factors (i.e., dignity, face, defense of family reputation, and self-promotion & retaliation) and a method factor to account for differences in response style (see Table S8 for primary loadings on substantive factors). Fit of the model was good (*χ^2^_[202]_* = 1055.677, *CFI* = .940, *TLI* = .931, *SRMR* = .048, *RMSEA* = .038). The loadings for all items were significant. The suggested changes in the modification indices for substantial factors were relatively low (all χ^2^ change values < 42). Furthermore, the suggested additional cross-loadings for items were generally low in strength (< .236 in absolute strength), and the respective primary loadings of items were at least twice as large in all cases; we therefore decided to retain all items for further analyses and did not add any cross-loadings to the model in this step.

In the third step, we then conducted **multigroup invariance testing** with the established four-factor structure. We tested invariance both across cultural regions, as well as gender groups (female vs male). An unconstrained model fit slightly better than a constrained model across cultural regions (Constrained: *χ^2^_[1192]_* = 3028.057, *CFI* = .881, *TLI* = .885, *SRMR* = .030, *RMSEA* = .051; Unconstrained: *χ^2^_[1120]_* = 2761.232, *CFI* = .894, *TLI* = .891, *SRMR* = .027, *RMSEA* = .050; *ΔCFI* = .013), but not across gender groups (Constrained: *χ^2^_[466]_* = 1237.099, *CFI* = .927, *TLI* = .927, *SRMR* = .026, *RMSEA* = .034; Unconstrained: *χ^2^_[448]_* = 1237.346, *CFI* = .925, *TLI* = .923, *SRMR* = .025, *RMSEA* = .035; *ΔCFI* = .002). We thus assumed invariance of our items across genders, but we followed up our invariance analysis on an item-by-item basis across regions. No items were flagged as non-invariant across regions by our criteria. Only one item (“*People should not care what others around them think.*” of the dignity factor) showed a non-significant loading on its factor in the Southeastern Europe group in the unconstrained model. However, this item did not show a substantial modification index in the constrained model, suggesting its loading in Southeastern Europe did not differ significantly from its loadings in the other regions; we therefore decided to retain the item. Nevertheless, based on parallel analyses testing invariance for the perceived normative value items (described above), we decided to exclude two items (“*People must always be ready to defend their honor*” of the factor for defense of family reputation, and “*It is important to promote oneself to others*” of the factor for self-promotion & retaliation), as these two items had been excluded following invariance testing in the perceived normative values, and we decided to keep the item selection comparable for both personal values and perceived normative values.

In a final step, we then used **multilevel confirmatory factor analysis** to model the between-samples factor structure of our data. To this end, we first added a factor structure at the between-samples level that mirrored our existing within-samples four-factor model; we tested models with loadings constrained across levels (i.e., complete isomorphism) or freely varying across levels (i.e., configural isomorphism). In both models, as in our multilevel models of perceived normative values, we found that the two dimensions of honor identified at the within-samples level (defense of family reputation, and self-promotion & retaliation) were almost perfectly correlated at the between-samples level (*r* = .992); we thus continued testing models merging the two dimensions at the higher level. We found that such a three-factor structure at the between-samples level (see Table S9 for the loadings on the substantive factors) was the most theoretically meaningful, interpretable, and fit the data acceptably (*χ^2^_[296]_* = 1179.375, *CFI* = .930, *TLI* = .919, *RMSEA* = .032, *SRMR_Within_* = .031, *SRMR_Between_* = .183). Items differed slightly between levels, with one item of the dignity factor (“*People should speak their mind.*”) and one item of the face factor (“*People should control their behavior in front of others.*”) being retained only at the within, but not at the between-samples level due to non-significant loadings; these items were therefore centered within samples. All between-samples factors showed significant variances in the final model.

### Personal Concerns

In a first step we again conducted an **exploratory factor analysis** to examine the underlying item structure for all honor concern items at the within-samples level. Items were uncentered and clustered by the 22 samples. As for perceived normative honor concerns, we mistakenly included one item from the original Honor Scale developed by Mosquera and colleagues (Rodriguez Mosquera et al., 2002) (*“...your sister or mother had the reputation of sleeping around*”) in place of an item from Guerra and colleagues (Guerra et al., 2013) short version (“*You were unable to defend your family’s reputation*”); as described above, we retained this item in our analyses. Again, a **four-factor solution** appeared to be the most theoretically meaningful solution, which matched the structure reported by the scale authors. We deemed a three-factor solution as less theoretically informative than a four-factor solution, as items showed higher cross-loadings and factors were more confounded, and several fit indices for the three-factor solution were unacceptable (*χ^2^_[75]_* = 1662.360, *CFI* = .874, *TLI* = .798, *RMSEA* = .085, *SRMR* = .043). A five-factor solution included a single item factor, as did a six-factor solution, and we therefore considered both of these solutions to be overfitted. The final four-factor solution showed excellent fit (*χ^2^_[62]_* = 250.922, *CFI* = .985, *TLI* = .971, *SRMR* = .015), and mirrored the factor structure found by Guerra and colleagues (Guerra et al., 2013), with all items showing their highest primary loadings on the expected factors, except for two items (“*....you were known as someone who cannot support a family”* and “*...you had the reputation of being someone without sexual experience?*”); we therefore excluded both of these items from further analysis. These exclusions left us with a total of 14 honor items (the same as for the models with perceived normative honor concerns, described earlier). As shown in Table S10, primary loadings for the remaining items were high (all above 0.45) with low cross-loadings (all below 0.3) for all items, except for one item (“*...your sister or mother had the reputation of sleeping around*”) which showed similar sized loadings on both its primary factor (family reputation: 0.470) and a secondary factor (sexual propriety: 0.430). Again, we interpreted the four factors as reflecting concerns about losing *family authority*, *family reputation*, *integrity*, and *sexual propriety*, adopting gender-neutral labels for the first and last factors.

In the second step, we conducted a **confirmatory factor analysis** of all concern items with six substantial factors (i.e., dignity, face, family reputation, sexual propriety, family authority, and integrity) as well as a method factor to account for differences in response style (see Table S11 for primary loadings on substantive factors). Fit of the model was very good (*χ^2^_[193]_* = 884.677, *CFI* = .967, *TLI* = .961, *SRMR* = .051, *RMSEA* = .035). The loadings for all items were significant. Cross-loadings as suggested by the modification indices for most items were low and less than half their respective primary loadings, with four exceptions: the item “*...your sister or mother had the reputation of sleeping around*” of the family reputation factor had a suggested cross-loading (0.497) on the sexual propriety factor;^[[1]](#footnote-2)^ the item “*...you did something to damage your family’s reputation?*” of the family reputation factor had a suggested cross-loading (-0.191) on the dignity factor; the item “*...you let other people insult your family?*” of the family reputation factor had suggested cross-loadings on the factors of face (-0.158) and sexual propriety (-0.163); the item “*...you failed to show humility about your achievements?*” of the face factor had suggested cross-loadings on the factors of integrity (0.193), dignity (0.153), and family reputation (-0.08); the item “*...you were hypocritical?*” of the integrity factor had suggested cross-loadings on the factors of dignity (0.106) and face (-0.159). However, since (a) the model already showed a good fit across all indices, (b) none of the suggested cross-loadings surpassed the items’ respective primary loading on their factor, and (c) the current four-factor structure with no cross-loadings allowed for better comparison of our results to previous studies that included the scale, we decided not to include any of these cross-loadings in the model, and to retain all items in the current structure for further analyses.

In the third step, we then conducted **multigroup invariance testing** with the established six factor structure. We tested invariance both across cultural regions, as well as gender groups (female and male). An unconstrained model did not fit better than a constrained model both across cultural regions (Constrained: *χ^2^_[1139]_* = 2943.995, *CFI* = .902, *TLI* = .901, *SRMR* = .032, *RMSEA* = .052; Unconstrained: *χ^2^_[1075]_* = 2854.545, *CFI* = .904, *TLI* = .896, *SRMR* = .030, *RMSEA* = .053; *ΔCFI* = .002) and across genders (Constrained: *χ^2^_[446]_* = 1219.622, *CFI* = .885, *TLI* = .881, *SRMR* = .040, *RMSEA* = .062; Unconstrained: *χ^2^_[430]_* = 1164.415, *CFI* = .891, *TLI* = .883, *SRMR* = .039, *RMSEA* = .062; *ΔCFI* = .006). We thus assumed invariance of our items across genders as well as across regions.

In a final step, we conducted a **multilevel confirmatory factor analysis** to model the between-samples factor structure of our data. To this end, we first added a between-samples factor structure that mirrored our existing within-samples six-factor model. We again started with a constrained model that set the factor structure and loadings to be equal at both levels of analysis; however, this model did not converge. Exploring various unconstrained models in which the factor structure and loadings at the between-samples level were allowed to differ from the within-samples level, we found that a model with four factors (i.e., dignity, family reputation, family authority, and sexual propriety) at the between-samples level was most theoretically meaningful, interpretable, and best fitting (see Table S12 for the loadings on the substantive factors in the final model). The model fit the data well (*χ^2^_[245]_* = 1148.925, *CFI* = .937, *TLI* = .924, *SRMR_Within_* = .040, *SRMR_Between_* = .161, *RMSEA* = .035). Factors and items differed substantially between levels: At the between-samples level, we excluded the factors of face and integrity, as models including these factors showed estimation problems, loading patterns were incoherent and non-significant, and these factors showed negligible between-samples variance; relevant items were therefore centered within samples. We further restricted two items of the family reputation factor (“*… you let other people insult your family?*” and “*…you did something to damage your family’s reputation?*” ) to the within-samples level only, due to non-significant loadings at the between-samples level; these items were therefore centered within samples. All factors at the between-samples level showed significant variances in the final model.

## Testing for Region Differences

Table S13 shows the correlations among the culture-level dimensions defined by factor scores saved from our final measurement models.

To test the statistical significance of pair-wise regional differences, we adapted our final measurement models by adding four dummy-coded (i.e., levels of “0” and “1”) variables that encoded participants’ regional origin and regressed the group-level factors onto these four dummy variables (representing tests for pair-wise regional differences). To ensure model stability given the small N at the cultural level of analysis, we fixed all factor loadings in these models to equal their estimates from the final measurement models. As this approach depends on the choice of a reference group for each model (i.e., the model for which all dummy-coded indicators are “0”, and to which the four other regions are compared to), we repeatedly recoded the reference group and reran the models in order to obtain all possible comparisons (see e.g., Hayes, 2017). For each measured dimension, we corrected for familywise error among the 10 possible pairwise comparisons using a Holm-Bonferroni sequentially adjusted alpha level (Holm, 1979); however, to guard against Type II error, we interpret as ‘marginal’ those findings that reached the conventional threshold for statistical significance (i.e., *p* ≤ .05) but did not meet the more stringent Holm-Bonferroni criterion. Table S14 to S17 show the complete list of estimates for each comparison for personal and perceived normative measures, separately.

## Testing for Gender Differences

Table S18 shows the pairwise comparisons between men and women on all sample-level dimensions from our final measurement models. To test the statistical significance of pair-wise gender differences, we again adapted our final measurement models by adding a dummy-coded (i.e., “0” for men and “1” for women) variable and regressing the group-level factors onto it, representing a pair-wise comparison of women against men as the reference group. To ensure model stability given the small N at the cultural level of analysis, we fixed all factor loadings in these models to equal their estimates from the final measurement models.

# Study 2: Supplementary Information

In Study 2, we wanted to establish whether certain findings observed in Study 1—including the unexpectedly low perceived normative honor values in Latin European samples and the differing regional profiles for personal endorsement versus perceived normative prevalence of honor values—could be replicated in general population samples from 14 societies. We focused on honor values, as this dimension provided a broad indication of the importance of honor in general in each society (rather than focusing on specific concerns such as authority or sexual propriety) and it had shown a clear pattern of regional differences in Study 1. Our analyses mapped culture-level variation in perceived normativity and personal endorsement of honor values across 28 cultural samples defined by the intersection of society and gender.

## Method

### Participants

In 14 sites (all Study 1 sites plus Canada, Tunisia, and the Turkish Cypriot Community in Cyprus), 6,577 participants (*Min* = 323 from the Greek-Cypriot Community, *Max* = 1,452 from Canada) answered our survey between September 2022 and February 2023. Samples were recruited by local or international research companies using national representative quotas based on characteristics such as age and gender. Participants were compensated with incentives adopted by the respective research company.

Participants had to be (a) at least 18 years old, (b) born in the country of data collection, and (c) residing in the country of data collection. In the Greek Cypriot community, we added nationality as a selection criterion following feedback from collaborators. We again retained participants who self-identified as female or male to allow for sizeable gender groups at the country level for comparative analyses. These inclusion criteria resulted in a final sample of 5,471 participants (not considering missing values; see Table 3 in the main article for a detailed overview of sample sizes and characteristics per site). We aimed for larger samples in each site than in Study 1 to increase the robustness of our analyses; only the Greek-Cypriot sample (147 men and 132 women) deviated from our target sample size of 200 men and 200 women, due to restrictions in the participant pool of the research company. The overall gender distribution was balanced (50.3% women; *M*_age_ = 42.61, *SD* = 15.12, range: 18 to 89). Self-reported SES (*M* = 5.59, *SD* = 1.94) averaged slightly above the midpoint of an 11-point scale from 0 (Bottom) to 10 (Top).

### Procedure

Measures were included in a larger study focusing on cultural logics and apologies. After providing consent, participants completed measures of perceived normative and personally endorsed honor values. Within each data collection site, we counterbalanced the order of rating personal honor values and perceived normative honor values. Finally, they provided demographic information before being thanked and debriefed. The study received ethical approval from the lead institution.

### Measures

**Honor Values.** Participants completed eight items that were retained in our measurement models for perceived normative and personal honor values in Study 1.^^[[2]](#footnote-3)^^ Items were unchanged, but we adjusted the rating scale for perceived normative values to maximize salience of the perspective participants were asked to take (1 = *Most people would strongly disagree* to 7 = *Most people would strongly agree*). Personal endorsement items were rated using a 7-point scale (1 = *Strongly disagree* to 7 = *Strongly agree*).

**Response Style.** To adjust for differences in response style in our measurement models, we created four indicators of acquiescent responding, each defined by averaging a pair of items with opposing substantive content from an unrelated measure (e.g., averaged agreement with “*I am unlikely to apologize if I have done something wrong*” and “*In general, I apologize after having done something wrong*” without reverse scoring). Items were rated using a 7-point scale (1 = *Strongly disagree* to 7 = *Strongly agree*). We used these items to anchor a method factor which allowed us to adjust our measures of honor values for acquiescent responding (see Supplementary Materials).

**Demographic Information.** Participants in all cultures reported their gender, age, country of birth, country where they attended high school, parents’ country of birth, parents’ highest education, length of stay in the country of data collection, native language, religious background, religiosity, and perceived SES in the country of residence (MacArthur Scale of Subjective Social Status: Adler et al., 2000). Self-reported ethnicity was again assessed in all countries except Lebanon and Egypt.

## Measurement Models

Measurement models adjusting for individual and cultural response styles replicated the internal factor structure of honor values previously observed in Study 1, showing a general *honor* factor at the between-samples level and two factors (*defense of family reputation*, and *self-promotion and retaliation*) at the within-samples level for both perceived normative and personally endorsed honor values.

### Model Selection Procedure

Analyses were again conducted using Mplus Version 8.5 (Muthén & Muthén, 1998), using the same criteria for model fit as described above for Study 1. As the focus in Study 2 for our measures of honor was on replicating the factor structure of our final models found in Study 1, we adjusted the steps of our model selection procedure accordingly and conducted our model selection in two steps separately for perceived normative and personal honor values:

First, we conducted a ***confirmatory factor analysis (CFA)*** on items assessing honor values to determine the most meaningful structure in these items at the individual level of analysis, using the TYPE=COMPLEX function in Mplus to account for clustering of individuals into 28 cultural samples. In the CFA, we modelled the found two factor structure at the within-sample level with one factor for the *defense of family reputation* (five items) and *self-promotion and retaliation* (three items). We did not model any previously included constraints on the error variances of single items. We again included an additional method factor in the CFA to model differences in response style. To create the method factor, we used two sets of four items from scale on the reluctance to apologize (as these items included both forward and reverse-coded items; four items to measure perceived societal norms, and four items to measure personal endorsement), and clustered these items into four pairs that were coded in opposite ways. Each pair contained one positively worded item and one reverse coded item that were similar in content, so that agreeing or disagreeing with both items simultaneously would be contradictory and thus a reflection of response tendencies rather than the substantive meaning of the items (for a similar approach see Alnabulsi et al., 2020). Depending on the fit of the CFA and potential errors, we again screened the fitted solution for necessary changes in the item structure.

In a second step, we then extended our confirmatory analysis to the between-samples level and conducted a ***multilevel confirmatory factor analysis*** to separately model our factors at the within- and between-samples (*N* = 28 samples) level. We started with the final structure found in the first step at the within-samples level and a one-factor structure for general honor at the between-samples level as found in Study 1. We again explored various alternative models with different structures at the between-samples level. Depending on model fit, we then refined the model structure at the between-samples level, until a final model had been reached that was theoretically meaningful and fit the data well.

### Perceived Normative Honor Values

In a first step, we conducted a **confirmatory factor analysis** modeling the final structure of perceived normative honor values at the within-samples level from Study 1, outlining a two-factor structure (one factor for defense of family reputation, and one factor for self-promotion & retaliation) as well as a method factor to account for differences in response style (see Table S19 for primary loadings on substantive factors). Fit of the model was good (*χ^2^_[56]_* = 835.338, *CFI* = .937, *TLI* = .925, *RMSEA* = .05, *SRMR* = .098), and loadings for all items were significant. Modification indices suggested some possible cross-loadings (highest χ^2^ change = 53.7); however, the suggested additional cross-loadings for items were generally low (< .155 in absolute terms) in comparison to the respective primary loadings (all > .577) which were at least twice as large in all cases. Given that the model already showed good fit indices, we therefore retained all items for further analyses.

In the next step, we then conducted a **multilevel confirmatory factor analysis** to model the factor structure of our data at the between-samples level. To this end, we added one general honor factor comprised of all items at the between-samples level, reflecting the final found structure of the perceived normative honor values in Study 1. The model showed only moderate fit in our new samples (*χ^2^_[113]_* = 1461.397, *CFI* = .886, *TLI* = .867, *RMSEA* = .047, *SRMR_Within_* = .088, *SRMR_Between_* = .174). As a potential alternative, we tested a model that mirrored the within-sample two-factor structure at the between-sample level; this model showed approximately the same degree of fit (*χ^2^_[114]_* = 1435.077, *CFI* = .886, *TLI* = .866, *RMSEA* = .047, *SRMR_Within_* = .088, *SRMR_Between_* = .174) and also showed extremely high correlations between the two factors at the between-sample level (*r* = .978), suggesting substantive overlap. We also compared our model with an alternative model that did not include the response style method factor, nor its additional indicators; this model fit the data substantially better (*χ^2^_[39]_* = 122.153, CFI = 0.989, TLI = 0.984, *RMSEA* = .020, *SRMR_Within_* = .020, *SRMR_Between_* = .049), suggesting that much of the misfit from our initial model was linked to the method factor rather than to characteristics of our substantive factors. Nonetheless freeing parameters within the method factor would have been theoretically inappropriate given the assumption that this should be capturing only variance that was not linked to item content. For these reasons, we decided to go ahead with a model with one general honor factor at the between samples level (see Table S20 for primary loadings on substantive factors). The model showed no modification indices at the between-samples level regarding the honor items; we therefore decided to not modify our model any further. The honor factor at the between-samples level showed significant variance in the final model.

### Personal Honor Values

In a first step, we conducted a **confirmatory factor analysis** modeling the final structure of person honor values at the within-samples level from Study 1, using a two-factor structure (one factor for defense of family reputation, and one factor for self-promotion & retaliation) as well as a method factor to account for differences in response style (see Table S21 for primary loadings on substantive factors). Fit of the final model was good (*χ^2^_[56]_* = 829.381, *CFI* = .925, *TLI* = .912, *RMSEA* = .050, *SRMR* = .085), and loadings for all items were significant. Again, some possible cross-loadings were suggested as modification indices (highest χ^2^ change = 41.2); however, the suggested additional cross-loadings for items were generally low (< .202 in absolute terms) in comparison to the respective primary loadings (all > .527) which were at least twice as large in all cases. Given that the model already showed good fit, we therefore decided to retain all items.

In the next step, we then conducted a **multilevel confirmatory factor analysis,** modeling the factor structure of our data at the between-samples level. We again started by adding one general honor factor comprised of all items at the between-samples level, reflecting the final found structure of the personal honor values in Study 1. We constrained the error variance of one items (“*People should be concerned about defending their families’ reputation*.”) to 0 at the between-samples level due to negative residual variance; this constraint emerged in all of the following model selection steps as well. The model showed only moderate fit in our new samples (*χ^2^_[114]_* = 1438.626, *CFI* = .877, *TLI* = .858, *RMSEA* = .046, *SRMR_Within_* = .078, *SRMR_Between_* = .164). We again compared this model with an alternative model that mirrored the within-sample two-factor structure at the between-sample level; this model fit showed approximately the same degree of fit (*χ^2^_[113]_* = 1435.077, *CFI* = .878, *TLI* = .857, *RMSEA* = .046, *SRMR_Within_* = .078, *SRMR_Between_* = .164) as the one factor model, but also showed high correlations between the two factors at the between-sample level (*r* = 0.978). We again also compared our model with an alternative model that did not include a method factor; this model fit the data significantly better (*χ^2^_[40]_* = 233.423, *CFI* = .969, *TLI* = .956, *RMSEA* = .030, *SRMR_Within_* = .034, *SRMR_Between_* = .050), again suggesting that misfit from the original model can be attributed to the method factor rather than to the substantive factors. For these reasons, we decided to again go ahead with a model with one general honor factor at the between samples level (see Table S22 for primary loadings on substantive factors). The model showed no further modification indices for honor items at the between-samples level; we thus decided to not modify our model any further. The honor factor at the between-samples level showed significant variance in the final model.

## Results

### Testing for Region Differences

Table S23 shows the correlations among the culture-level dimensions defined by factor scores saved from our final measurement models.

As in Study 1, we grouped the 14 collection sites into five regions (categorizing Canada as *Anglo-Western*, and Tunisia and Turkish-Cypriot Community within the *MENA* region). Figure 5 (in the main article) illustrates the patterns of regional differences across perceived normative and personally endorsed honor values using culture-level factor scores saved from our measurement models. To test the statistical significance of pair-wise regional differences, we again adapted our final measurement models by adding four dummy-coded (i.e., levels of “0” and “1”) variables that encoded participants’ regional origin and regressed the group-level factors onto these four dummy variables (representing tests for pair-wise regional differences). To ensure model stability given the small N at the cultural level of analysis, we again fixed all factor loadings in these models to equal their estimates from the final measurement models. As this approach depends on the choice of a reference group for each model (i.e., the model for which all dummy-coded indicators are “0”, and to which the four other regions are compared to), we repeatedly recoded the reference group and reran the models in order to obtain all possible comparisons (see e.g., Hayes, 2017). For each measured dimension, we corrected for familywise error among the 10 possible pairwise comparisons using a Holm-Bonferroni sequentially adjusted alpha level (Holm, 1979); however, to guard against Type II error, we interpret as ‘marginal’ those findings that reached the conventional threshold for statistical significance (i.e., *p* ≤ .05) but did not meet the more stringent Holm-Bonferroni criterion. Table S24 shows the complete list of estimates for each comparison for personal and perceived normative honor, separately (for a briefer overview, see Table 4, in the main text).

**Perceived normative honor values*.*** These general population samples showed a highly similar pattern of cultural differences in perceived normative honor values to our Study 1 student samples. MENA samples again perceived stronger normative endorsement of honor values than all other samples (*p*s < .001), followed by Southeast European samples whose perceptions of honor values were marginally stronger than Latin European samples (*p* = .05), and significantly stronger than all remaining regions (*p*s < .001). Among the remaining three regions the only difference was that Latin-European samples showed marginally stronger perceived normative honor values than Anglo-Western samples (*p* = .021).

**Personal honor values*.*** Personal endorsement of honor values also largely replicated the pattern observed in Study 1. MENA samples again endorsed honor values more strongly than samples in all other regions (*p* < .001). Few differences emerged among the remaining samples, except that East Asian samples endorsed honor values significantly more than Anglo-Western samples (*p* = .002) and marginally more than Southeast European samples (*p* = .015).

### Gender Comparisons

Perceived normative and personally endorsed honor values were again remarkably consistent across women and men (see Figure 6 in the main text, and Table S25). Across 14 societies, scores for samples of women and men showed highly positive and significant correlations for perceived normative honor values (*r* = .980, *p* < .001) and personally endorsed honor values (*r* = .980, *p* < .001). We tested for gender differences by adapting our measurement models in the same way as in Study 1. We found no significant gender differences in perceived normativity, nor in personal endorsement of honor values.

### Convergence with Study 1

Across the 22 cultural groups included in Study 1, factor scores representing culture-level variance in honor values were highly correlated across the two studies (perceived normative honor values: *r* = .90; personally endorsed honor values: *r* = .82). This close convergence supports the validity of relying on student participants in Study 1, especially as informants about the normative prevalence of honor values in the societies they inhabited.

### Exploring Age Trends Within Samples

Given the much wider age range in Study 2, compared to Study 1, we were interested to explore age trends in the within-samples factor scores for perceived normative and personally endorsed honor values. On average, defense of family reputation values were slightly higher among older than among younger participants (perceived normative honor values: *r* = .14, *p* < .001; personal honor values: *r* = .17, *p* < .001), whereas self-promotion and retaliation values were slightly higher among younger than older participants (perceived normative honor values: *r* = -.03, *p* = .034; personal honor values: *r* = -.07, *p* < .001). However, as shown in Table S26, our cultural samples showed considerable variation in these age trends.

To explore for systematic geographical variation in age trends across the five regions in our study, we ran a series of multilevel regression models regressing each of the within-samples factor scores on age (grand mean centered, in decades), region (coded with four dummy contrasts), and the cross-level interactions of age with region. We ran five versions of each analysis in order to obtain simple slopes for the age trends in each region, as well as a full set of pairwise comparisons across regions. As in our previous regional comparisons, we corrected for familywise error among the 10 possible pairwise comparisons using a Holm-Bonferroni sequentially adjusted alpha level, starting from .05/10 = .005 (Holm, 1979). Results of these analyses are summarized in Table S27.

For *defense of family reputation* values, we found significantly stronger age trends in personal values in Southeast European samples compared to Anglo-Western and MENA samples; Latin European and East Asian samples showed intermediate age trends that did not differ significantly from either extreme. Thus, whilst older participants in all regions tended to place a higher value on defense of family reputation, this age trend was strongest in Southeast Europe, suggesting the possibility of generational differences in this region. Note, however, that we did not find significant regional differences in the tendency for older participants to perceive defense of family reputation as more normative within their societies.

For *self-promotion and retaliation* values, we found that age trends in both personal and perceived normative values were significantly higher in Anglo-Western samples than in any other region. Thus, tendencies for younger participants to place a higher value on self-promotion and retaliation, and to perceive this as normative, appeared to be wholly attributable to our Anglo-Western samples, whereas no such tendencies were found in other regions. For perceived normative values, but not personal values, we found a slight trend in the opposite direction among MENA samples, such that older, rather than younger, participants in this region tended on average to perceive self-promotion and retaliation as more normative.

# Study 3: Supplementary Information

We conducted a series of multilevel mediation analyses seeking to provide a first test of the predictive utility of honor values to account for the pattern of regional differences in social orientation and cognitive style, specifically the results provided by Uskul, Kirchner-Häusler, and colleagues (2023). The goal was to evaluate the extent to which cultural variations in perceived normative and personally endorsed honor values would explain these previously reported average differences in social orientation and cognitive style between samples from Mediterranean societies and those from Anglo-Western or East Asian societies (described in more detail below).

To test this research question, we again used the data of Study 1, which also included ten further tasks to measure aspects of social orientation and cognitive style (Uskul et al., 2023) (see Table 5 in the main text). In particular, we sought to test the prediction by Uskul, Kirchner-Häusler, and colleagues (2023) that a cultural logic of honor might account for differences observed between Mediterranean and non-Mediterranean societies on four of these measures (our *a-priori hypotheses*): tendencies to experience socially disengaging more than engaging emotions, to link happiness more to socially disengaging rather than engaging emotions, to view the self as ‘inflated’ relative to others, and to remember events from a third rather than a first-person perspective. We decided to examine the role of honor in the remaining six tasks in an exploratory manner (*exploratory analyses*).

To test the mediation effects when it came to the four measures with *a-priori hypotheses*, we conducted a series of multilevel mediation models. Since perceived normative and personal endorsement measures of honor values were strongly correlated (*r* = .654) at the between-samples level, we conducted separate sets of analyses for these two mediators (resulting in 8 models: four for perceived normative honor, and four for personal honor). Please refer to the main text for more details; results of these models can be found in Tables 6 and 7.

## Exploratory Analyses

For the six additional indices of social cognitive tendencies, we tested exploratory models parallel to our main mediation analyses for *a priori* hypotheses. As in our main analyses, at the between-samples level, we modeled variation in each dependent measure as a function of cultural region, coded with two orthogonal contrast variables: the first indicating category membership in Anglo-Western versus East Asian societies (coded: -0.5 = East Asian societies, 0.5 = Anglo-Western societies, 0 = Mediterranean societies) and the second, our focal contrast, indicating category membership in Mediterranean societies (coded: -0.5 = East Asian societies, -0.5 = Anglo-Western societies, 0.5 = Mediterranean societies). We included factor scores for honor values as a potential mediator of variation across the three regions in each dependent measure. Since perceived normative and personal endorsement measures of honor values were strongly correlated (*r* = .654) at the between-samples level, we conducted separate sets of analyses for these two mediators, resulting in 12 exploratory models (six for perceived normative honor, six for personal honor). Accordingly, we corrected the significance levels of mediation effects in these twelve models using Holm-Bonferroni corrections with a sequentially adjusted significance threshold starting from .05/12 = .0042. To guard against Type II error, in all analyses we again interpreted as ‘marginal’ those findings that reached a conventional threshold for statistical significance (i.e., *p* ≤ .05) but did not meet our adjusted criterion.

All mediation analyses were conducted using Mplus Version 8.5 (Muthén & Muthén, 1998). All participants were again clustered in their respective gender groups in each society. Estimates for each indirect mediation effect were obtained using the “*model indirect*” command. In all analyses, we also included paths exploring whether within-sample variance in each outcome would be explained by the two dimensions of within-sample variation in honor values (*defense of family reputation*, and *self-promotion and retaliation*), as well as age and socio-economic status. Results of these models can be found in Tables S28 and S29.

## Exploratory Results

As shown in Table S28, we found evidence of indirect effects through perceived normative honor values for two of the six outcomes with exploratory analyses: lower nepotism in reward situations (tendency to reward friends more than strangers for the same behavior), and a tendency to attribute behavior to dispositional rather than situational causes. For nepotism in reward situations, Mediterranean societies were perceived to be higher in honor endorsement (*ß* = 0.687, *p* < .001; as in all models), and perceived normative honor endorsement predicted *lower* nepotism (*ß* = -0.810, *p* < .001), resulting in a significant negative indirect effect: *ß* = -0.557, *p* < .001. For dispositional attribution bias, perceived normative honor endorsement predicted this outcome positively (*ß* = 0.339, *p* = .015); however, the resulting positive indirect effect only reached our threshold of marginal significance: *ß* = 0.233, *p* = .027. We found no further signs of mediation in the remaining variables.

As shown in Table S29, we found no evidence of mediation by personally endorsed honor values, given that Mediterranean cultural groups did not score significantly higher overall in personal endorsement of honor values (*ß* = 0.125, *p* = .436). Nevertheless, culture-level variation in personal honor values significantly predicted lower nepotism in reward situations and somewhat more inclusion of contextual information when deciding what was relevant in a scenario-based task (see Table S29).

## Within-Sample Paths

Within-sample paths in our main analyses, shown in Tables 6 and 7 in the main article, reveal small relationships between the two dimensions of honor values and selected outcome measures, which were slightly stronger for personal values than for perceived normative values. Notably, individuals within each sample who scored higher on the self-promotion and retaliation dimension of personal values showed a somewhat greater tendency to experience disengaging (vs. engaging) emotions (*β* = .168, *p* < .001) and a slight tendency to remember events from a third-person memory perspective (*β* = .042, *p* = .032), apparently mirroring at the individual level our culture-level findings that these two outcomes were more prevalent in cultural samples with higher perceived normative honor values. However, the second of these relationships was complicated by a countervailing effect such that individuals who valued defense of family reputation to a greater extent were *less* likely to remember events from a third-person memory perspective (*β* = -.072, *p* = .004).

Within-sample relationships in our exploratory analyses, shown in Tables S28 and S29, similarly showed small and sometimes complex associations with social cognitive outcomes, which again tended to be slightly stronger for personally endorsed values than for perceived normative values. Similar to our main findings for third-person memory perspective, within-sample individual differences in dispositional attribution were predicted in opposite directions by the two individual-level honor dimensions—higher among those who valued defense of family reputation, but lower among those who valued self-promotion and retaliation to a greater extent. In contrast, within-sample variation in nepotism in reward situations was not predicted by either dimension of honor values.

Overall, neither the pattern of within-sample relationships nor their magnitude suggested that culture-level associations of honor values with social cognitive tendencies could be reduced to an individual-level explanation. Thus, our findings seemingly reflect the social cognitive implications of living in a society where honor values are normatively perceived as prevalent—i.e., where a certain “cultural logic” prevails—rather than being explicable as aggregated effects of individuals’ personal endorsement of honor values, nor even as effects of their individual perceptions of their cultural environments. This is consistent with viewing honor logic as a property of cultural groups, not individuals, as we discuss further below.

# References

Adler, N. E., Epel, E. S., Castellazzo, G., & Ickovics, J. R. (2000). Relationship of subjective and objective social status with psychological and physiological functioning: Preliminary data in healthy, White women. *Health Psychology*, *19*(6), 586–592.

Alnabulsi, H., Drury, J., Vignoles, V. L., & Oogink, S. (2020). Understanding the impact of the Hajj: Explaining experiences of self-change at a religious mass gathering. *European Journal of Social Psychology*, *50*(2), 292–308.

Asparouhov, T., & Muthén, B. (2018). *SRMR in Mplus. Technical appendix*. Los Angeles, CA: Muthén & Muthén.[Google Scholar].

Guerra, V. M., Giner-Sorolla, R., & Vasiljevic, M. (2013). The importance of honor concerns across eight countries. *Group Processes & Intergroup Relations*, *16*(3), 298–318.

Hayes, A. F. (2017). *Introduction to Mediation, Moderation, and Conditional Process Analysis, Second Edition: A Regression-Based Approach*. Guilford Publications.

Holm, S. (1979). A Simple Sequentially Rejective Multiple Test Procedure. *Scandinavian Journal of Statistics*, *6*(2), 65–70.

Hu, L., & Bentler, P. M. (1999). Cutoff criteria for fit indexes in covariance structure analysis: Conventional criteria versus new alternatives. *Structural Equation Modeling: A Multidisciplinary Journal*, *6*(1), 1–55.

Kline, R. B. (2023). *Principles and Practice of Structural Equation Modeling*. Guilford Publications.

Marsh, H. W., Hau, K.-T., & Wen, Z. (2004). In Search of Golden Rules: Comment on Hypothesis-Testing Approaches to Setting Cutoff Values for Fit Indexes and Dangers in Overgeneralizing Hu and Bentler’s (1999) Findings. *Structural Equation Modeling: A Multidisciplinary Journal*, *11*(3), 320–341.

Mensah, Y. M., & Chen, H.-Y. (2012). Global Clustering of Countries by Culture – An Extension of the GLOBE Study. *SSRN Electronic Journal*.

Muthén, L., & Muthén, B. (1998). *Mplus user’s guide* (8th ed.). Muthén & Muthén.

Rodriguez Mosquera, P. M., Manstead, A. S. R., & Fischer, A. H. (2002). The role of honour concerns in emotional reactions to offences. *Cognition and Emotion*, *16*(1), 143–163.

Uskul, A. K., Kirchner-Häusler, A., Vignoles, V. L., Rodriguez-Bailón, R., Castillo, V. A., Cross, S. E., Yalçın, M. G., Harb, C., Husnu, S., Ishii, K., Jin, S., Karamaouna, P., Kafetsios, K., Kateri, E., Matamoros-Lima, J., Liu, D., Miniesy, R., Na, J., Özkan, Z., … Uchida, Y. (2023). Neither Eastern nor Western: Patterns of independence and interdependence in Mediterranean societies. *Journal of Personality and Social Psychology*, *125*(3), 471–495.

Welkenhuysen-Gybels, J., Billiet, J., & Cambré, B. (2003). Adjustment for Acquiescence in the Assessment of the Construct Equivalence of Likert-Type Score Items. *Journal of Cross-Cultural Psychology*, *34*(6), 702–722.

## Table S1

#### Exploratory Factor Analysis Loadings of Perceived Normative Honor Value Items (Two-Factor Solution) (Study 1)

| **Item** | **Defense of  Family Reputation** | **Self-Promotion &  Retaliation** |
| --- | --- | --- |
| People should be concerned about defending their families’ reputation. | **.828** | .007 |
| People should be concerned about damaging their families’ reputation. | **.800** | -.049 |
| People should be concerned about their family having a bad reputation. | **.742** | .076 |
| People should not allow others to insult their family. | **.706** | -.048 |
| People must always be ready to defend their honor. | **.499** | .291 |
| Men need to protect their women’s reputation at all costs. | **.432** | .271 |
| People always need to show off their power in front of their competitors. | -.023 | **.788** |
| If a person gets insulted and they don’t respond, he or she will look weak. | -.001 | **.695** |
| You must punish people who insult you. | .059 | **.654** |
| It is important to promote oneself to others. | .059 | **.547** |
| *Note.* Shown are the standardized loadings for the final two-factor solution of the exploratory factor analysis conducted with the perceived normative honor value items (sorted by primary loading strength on each factor). | | |

## Table S2

#### Confirmatory Factor Analysis Loadings of Perceived Normative Value Items (Four-Factor Solution) (Study 1)

| **Item** | **Dignity** | **Face** | **Defense of  Family Reputation** | **Self-Promotion & Retaliation** |
| --- | --- | --- | --- | --- |
| People should be true to themselves regardless of what others think. | .692 |  |  |  |
| People should make decisions based on their own opinions and not based on what others think. | .663 |  |  |  |
| People should stand up for what they believe in even when others disagree. | .571 |  |  |  |
| People should speak their mind. | .552 |  |  |  |
| People should not care what others around them think. | .539 |  |  |  |
| How much a person respects himself is far more important than how much others respect him. | .523 |  |  |  |
| People should be extremely careful not to embarrass others. |  | .574 |  |  |
| People should be very humble to maintain good relationships. |  | .539 |  |  |
| People should never criticize others in public. |  | .516 |  |  |
| People should minimize conflict in social relationships at all costs. |  | .492 |  |  |
| People should control their behavior in front of others. |  | .485 |  |  |
| It is important to maintain harmony within one’s group. |  | .463 |  |  |
| People should be concerned about defending their families’ reputation. |  |  | .672 |  |
| People should be concerned about their family having a bad reputation. |  |  | .657 |  |
| People should be concerned about damaging their families’ reputation. |  |  | .603 |  |
| People must always be ready to defend their honor. |  |  | .470 |  |
| Men need to protect their women’s reputation at all costs. |  |  | .463 |  |
| People should not allow others to insult their family. |  |  | .453 |  |
| If a person gets insulted and they don’t respond, he or she will look weak. |  |  |  | .641 |
| People always need to show off their power in front of their competitors. |  |  |  | .634 |
| You must punish people who insult you. |  |  |  | .581 |
| It is important to promote oneself to others. |  |  |  | .409 |
| *Note.* Shown are the standardized loadings for the final four-factor solution of the within-samples confirmatory factor analysis conducted with the perceived normative value items (sorted by primary loading strength on each factor). | | | | |

## Table S3

#### Multilevel Confirmatory Factor Analysis Loadings of Perceived Normative Value Items (Study 1)

| **Item** | **Within-Samples (Level 1)** | | | |  | **Between-Samples (Level 2)** | | |
| --- | --- | --- | --- | --- | --- | --- | --- | --- |
|  | **Dignity** | **Face** | **Defense of**  **Family Reputation** | **Self-Promotion & Retaliation** |  | **Dignity** | **Face** | **Honor** |
| People should stand up for what they believe in even when others disagree. | .577 |  |  |  |  | .960 |  |  |
| People should not care what others around them think. | .541 |  |  |  |  | .927 |  |  |
| People should speak their mind. | .546 |  |  |  |  | .912 |  |  |
| People should be true to themselves regardless of what others think. | .686 |  |  |  |  | .843 |  | -.471 |
| People should make decisions based on their own opinions and not based on what others think. | .674 |  |  |  |  | .684 |  | -.644 |
| How much a person respects himself is far more important than how much others respect him. | .552 |  |  |  |  | .644 |  |  |
| It is important to maintain harmony within one’s group. |  | .447 |  |  |  |  | .878 |  |
| People should control their behavior in front of others. |  | .473 |  |  |  |  | .837 |  |
| People should be very humble to maintain good relationships. |  | .522 |  |  |  |  | .782 |  |
| People should be extremely careful not to embarrass others. |  | .584 |  |  |  |  | .778 |  |
| People should never criticize others in public. |  | .550 |  |  |  |  | - |  |
| People should minimize conflict in social relationships at all costs. |  | .492 |  |  |  |  | - |  |
| You must punish people who insult you. |  |  |  | .561 |  |  |  | .973 |
| People should be concerned about defending their families’ reputation. |  |  | .634 |  |  |  |  | .971 |
| People should be concerned about their family having a bad reputation. |  |  | .633 |  |  |  |  | .967 |
| People should be concerned about damaging their families’ reputation. |  |  | .596 |  |  |  |  | .952 |
| Men need to protect their women’s reputation at all costs. |  |  | .352 |  |  |  |  | .927 |
| People should not allow others to insult their family. |  |  | .419 |  |  |  |  | .923 |
| If a person gets insulted and they don’t respond, he or she will look weak. |  |  |  | .668 |  |  |  | .903 |
| People always need to show off their power in front of their competitors. |  |  |  | .570 |  |  |  | .827 |

*Note.* Shown are the standardized loadings for the final solution of the multilevel confirmatory factor analysis conducted with the perceived normative value items (with four factors at the within-samples and three factors at the between-samples level; items are sorted by primary loading strength on between-samples level factors).

## Table S4

#### Exploratory Factor Analysis Loadings of Perceived Normative Honor Concern Items (Four-Factor Solution) (Study 1)

| **Items** | **Family Authority** | **Family  Reputation** | **Integrity** | **Sexual Propriety** |
| --- | --- | --- | --- | --- |
| ...you lacked authority over your own family? | **.849** | -.013 | .009 | .020 |
| ...you were known as someone who lacks authority over your own family? | **.843** | .033 | -.004 | .020 |
| *...you were known as someone who cannot support a family?* | *.248* | *.469* | *.146* | *-.004* |
| *...you had the reputation of being someone without sexual experience?* | *.233* | *.194* | *-.166* | *-.066* |
| ...your family had a bad reputation? | .016 | **.689** | .000 | .136 |
| ...you let other people insult your family? | -.023 | **.674** | .150 | -.045 |
| ...you did something to damage your family’s reputation? | -.010 | **.609** | .268 | .018 |
| ...your sister or mother had the reputation of sleeping around? | .053 | **.562** | -.146 | .281 |
| ...you lied to others? | -.012 | -.035 | **.816** | .028 |
| ...you did not keep your word? | .052 | -.001 | **.801** | -.007 |
| ...you were hypocritical? | -.041 | .044 | **.774** | .009 |
| ...you had the reputation of being dishonest with others? | .028 | .187 | **.633** | .015 |
| ...you were known as someone who has had many different sexual partners? | -.031 | .043 | .002 | **.850** |
| ...you slept with someone without starting a serious relationship with that person? | .033 | -.083 | .083 | **.786** |
| ...you were known as someone whom it is easy to sleep with? | -.037 | .224 | -.026 | **.711** |
| ...you changed boyfriend/girlfriend often? | .071 | .007 | .255 | **.558** |
| *Note.* Shown are the standardized loadings for the final four-factor solution of the exploratory factor analysis conducted with the perceived normative honor concern items (sorted by primary loading strength on each factor). Italicized items were excluded at this step from future analyses. | | | | |

## Table S5

#### Confirmatory Factor Analysis Loadings of Perceived Normative Concern Items (Six-Factor Solution) (Study 1)

| **Item** | **Family Reputation** | **Sexual Propriety** | **Family authority** | **Integrity** | **Face** | **Dignity** |
| --- | --- | --- | --- | --- | --- | --- |
| ...your sister or mother had the reputation of sleeping around? | .523 |  |  |  |  |  |
| ...your family had a bad reputation? | .401 |  |  |  |  |  |
| ...you let other people insult your family? | .180 |  |  |  |  |  |
| ...you did something to damage your family’s reputation? | .129 |  |  |  |  |  |
| ...you were known as someone who has had many different sexual partners? |  | .742 |  |  |  |  |
| ...you slept with someone without starting a serious relationship with that person? |  | .663 |  |  |  |  |
| ...you were known as someone whom it is easy to sleep with? |  | .624 |  |  |  |  |
| ...you changed boyfriend/girlfriend often? |  | .494 |  |  |  |  |
| ...you were known as someone who lacks authority over your own family? |  |  | .717 |  |  |  |
| ...you lacked authority over your own family? |  |  | .677 |  |  |  |
| ...you lied to others? |  |  |  | .633 |  |  |
| ...you were hypocritical? |  |  |  | .584 |  |  |
| ...you did not keep your word? |  |  |  | .572 |  |  |
| ...you had the reputation of being dishonest with others? |  |  |  | .382 |  |  |
| ...you criticized somebody else in public? |  |  |  |  | .569 |  |
| ...you failed to show humility about your achievements? |  |  |  |  | .481 |  |
| ...you caused conflict in your social relationships? |  |  |  |  | .383 |  |
| ...you lost control over your behavior in front of others? |  |  |  |  | .336 |  |
| ...you were not true to yourself? |  |  |  |  |  | .559 |
| ...you made decisions based on others’ opinions and not your own? |  |  |  |  |  | .464 |
| ...you did not stand up for what you believe? |  |  |  |  |  | .443 |
| ...you cared about what others think of you more than your own thoughts? |  |  |  |  |  | .405 |
| *Note.* Shown are the standardized loadings for the final six-factor solution of the within-samples confirmatory factor analysis conducted with the perceived normative concern items (sorted by primary loading strength on each factor). | | | | | | |

## Table S6

#### Multilevel Confirmatory Factor Analysis Loadings of Perceived Normative Concern Items (Study 1)

| **Item** | **Within-Samples (Level 1)** | | | | | |  | **Between-Samples (Level 2)** | | | | | |
| --- | --- | --- | --- | --- | --- | --- | --- | --- | --- | --- | --- | --- | --- |
|  | **Family**  **Reputation** | **Sexual Propriety** | **Family Authority** | **Integrity** | **Face** | **Dignity** |  | **Family Reputation** | **Sexual Propriety** | **Family Authority** | **Integrity** | **Face** | **Dignity** |
| ...your family had a bad reputation? | .313 |  |  |  |  |  |  | .949 |  |  |  |  |  |
| ...you did something to damage your family’s reputation? | .063 |  |  |  |  |  |  | .865 |  |  |  |  |  |
| ...your sister or mother had the reputation of sleeping around? | .431 |  |  |  |  |  |  | .839 |  |  |  |  |  |
| ...you let other people insult your family? | .120 |  |  |  |  |  |  | .802 |  |  |  |  |  |
| ...you were known as someone who has had many different sexual partners? |  | .685 |  |  |  |  |  |  | .980 |  |  |  |  |
| ...you were known as someone whom it is easy to sleep with? |  | .567 |  |  |  |  |  |  | .944 |  |  |  |  |
| ...you slept with someone without starting a serious relationship with that person? |  | .600 |  |  |  |  |  |  | .922 |  |  |  |  |
| ...you changed boyfriend/girlfriend often? |  | .495 |  |  |  |  |  |  | .852 |  |  |  |  |
| ...you were known as someone who lacks authority over your own family? |  |  | .680 |  |  |  |  |  |  | .958 |  |  |  |
| ...you lacked authority over your own family? |  |  | .685 |  |  |  |  |  |  | .957 |  |  |  |
| ...you lied to others? |  |  |  | .628 |  |  |  |  |  |  | .881 |  |  |
| ...you did not keep your word? |  |  |  | .575 |  |  |  |  |  |  | .788 |  |  |
| ...you were hypocritical? |  |  |  | .591 |  |  |  |  |  |  | .729 |  |  |
| ...you had the reputation of being dishonest with others? |  |  |  | .383 |  |  |  |  |  |  | - |  |  |
| ...you criticized somebody else in public? |  |  |  |  | .560 |  |  |  |  |  |  | .896 |  |
| ...you caused conflict in your social relationships? |  |  |  |  | .376 |  |  |  |  |  |  | .640 |  |
| ...you failed to show humility about your achievements? |  |  |  |  | .483 |  |  |  |  |  |  | .530 |  |
| ...you lost control over your behavior in front of others? |  |  |  |  | .320 |  |  |  |  |  |  | - |  |
| ...you made decisions based on others’ opinions and not your own? |  |  |  |  |  | .473 |  |  |  |  |  |  | .917 |
| ...you cared about what others think of you more than your own thoughts? |  |  |  |  |  | .403 |  |  |  |  |  |  | .903 |
| ...you were not true to yourself? |  |  |  |  |  | .548 |  |  |  |  |  |  | .740 |
| ...you did not stand up for what you believe? |  |  |  |  |  | .446 |  |  |  |  |  |  | - |

*Note.* Shown are the standardized loadings for the final solution of the multilevel confirmatory factor analysis conducted with the perceived normative concern items (with six factors at the within-samples and six factors at the sample-level; items are sorted by primary loading strength on sample-level factors).

## Table S7

#### Exploratory Factor Analysis Loadings of Own Honor Value Items (Two-Factor Solution) (Study 1)

| **Items** | **Defense of  Family Reputation** | **Self-Promotion &  Retaliation** |
| --- | --- | --- |
| People should be concerned about their family having a bad reputation. | **.779** | .045 |
| People should be concerned about defending their families’ reputation. | **.871** | -.004 |
| People should be concerned about damaging their families’ reputation. | **.867** | -.095 |
| People should not allow others to insult their family. | **.558** | .076 |
| Men need to protect their women’s reputation at all costs. | **.476** | .220 |
| People must always be ready to defend their honor. | **.463** | .314 |
| People always need to show off their power in front of their competitors. | -.003 | **.677** |
| You must punish people who insult you. | .026 | **.617** |
| If a person gets insulted and they don’t respond, he or she will look weak. | -.072 | **.547** |
| It is important to promote oneself to others. | .019 | **.485** |
| *Note.* Shown are the standardized loadings for the final two-factor solution of the exploratory factor analysis conducted with the perceived normative honor value items (sorted by primary loading strength on each factor). | | |

## Table S8

#### Confirmatory Factor Analysis Loadings of Own Value Items (Four-Factor Solution) (Study 1)

| **Item** | **Dignity** | **Face** | **Defense of  Family Reputation** | **Self-Promotion &  Retaliation** |
| --- | --- | --- | --- | --- |
| People should be true to themselves regardless of what others think. | .554 |  |  |  |
| People should stand up for what they believe in even when others disagree. | .522 |  |  |  |
| People should make decisions based on their own opinions and not based on what others think. | .472 |  |  |  |
| People should not care what others around them think. | .445 |  |  |  |
| How much a person respects himself is far more important than how much others respect him. | .293 |  |  |  |
| People should speak their mind. | .286 |  |  |  |
| People should minimize conflict in social relationships at all costs. |  | .604 |  |  |
| People should be extremely careful not to embarrass others. |  | .486 |  |  |
| People should be very humble to maintain good relationships. |  | .449 |  |  |
| People should never criticize others in public. |  | .423 |  |  |
| It is important to maintain harmony within one’s group. |  | .385 |  |  |
| People should control their behavior in front of others. |  | .341 |  |  |
| People should be concerned about defending their families’ reputation. |  |  | .796 |  |
| People should be concerned about their family having a bad reputation. |  |  | .756 |  |
| People should be concerned about damaging their families’ reputation. |  |  | .750 |  |
| Men need to protect their women’s reputation at all costs. |  |  | .556 |  |
| People must always be ready to defend their honor. |  |  | .552 |  |
| People should not allow others to insult their family. |  |  | .493 |  |
| You must punish people who insult you. |  |  |  | .608 |
| People always need to show off their power in front of their competitors. |  |  |  | .545 |
| If a person gets insulted and they don’t respond, he or she will look weak. |  |  |  | .503 |
| It is important to promote oneself to others. |  |  |  | .378 |

*Note.* Shown are the standardized loadings for the final four-factor solution of the within-samples confirmatory factor analysis conducted with the perceived normative value items (sorted by primary loading strength on each factor).

## Table S9

#### Multilevel Confirmatory Factor Analysis Loadings of Personal Value Items (Study 1)

| **Item** | **Within-Samples (Level 1)** | | | |  | **Between-Samples (Level 2)** | | |
| --- | --- | --- | --- | --- | --- | --- | --- | --- |
|  | **Dignity** | **Face** | **Defense of  Family Reputation** | **Self-Promotion &  Retaliation** |  | **Dignity** | **Face** | **Honor** |
| People should not care what others around them think. | .292 |  |  |  |  | .955 |  |  |
| People should be true to themselves regardless of what others think. | .490 |  |  |  |  | .799 |  |  |
| People should stand up for what they believe in even when others disagree. | .475 |  |  |  |  | .793 |  |  |
| People should make decisions based on their own opinions and not based on what others think. | .479 |  |  |  |  | .613 |  |  |
| How much a person respects himself is far more important than how much others respect him. | .271 |  |  |  |  | .509 |  |  |
| People should speak their mind. | .307 |  |  |  |  | - |  |  |
| People should minimize conflict in social relationships at all costs. |  | .534 |  |  |  |  | .816 |  |
| People should be extremely careful not to embarrass others. |  | .454 |  |  |  |  | .694 |  |
| People should never criticize others in public. |  | .359 |  |  |  |  | .563 |  |
| People should be very humble to maintain good relationships. |  | .407 |  |  |  |  | .555 |  |
| It is important to maintain harmony within one’s group. |  | .400 |  |  |  |  | .292 |  |
| People should control their behavior in front of others. |  | .381 |  |  |  |  | - |  |
| People should be concerned about their family having a bad reputation. |  |  | .746 |  |  |  |  | .888 |
| People should be concerned about defending their families’ reputation. |  |  | .770 |  |  |  |  | .827 |
| Men need to protect their women’s reputation at all costs. |  |  | .491 |  |  |  |  | .819 |
| People should be concerned about damaging their families’ reputation. |  |  | .758 |  |  |  |  | .805 |
| You must punish people who insult you. |  |  |  | .628 |  |  |  | .755 |
| People always need to show off their power in front of their competitors. |  |  |  | .449 |  |  |  | .730 |
| People should not allow others to insult their family. |  |  | .476 |  |  |  |  | .599 |
| If a person gets insulted and they don’t respond, he or she will look weak. |  |  |  | .554 |  |  |  | .504 |

*Note.* Shown are the standardized loadings for the final solution of the multilevel confirmatory factor analysis conducted with the personal value items (with four factors at the within-samples and three factors at the sample-level; items are sorted by primary loading strength on sample-level factors).

## Table S10

#### Exploratory Factor Analysis Loadings of Own Honor Concern Items (Four-Factor Solution) (Study 1)

| **Items** | **Family Authority** | **Family  Reputation** | **Integrity** | **Sexual Propriety** |
| --- | --- | --- | --- | --- |
| ...you lacked authority over your own family? | **.871** | -.016 | .011 | .003 |
| ...you were known as someone who lacks authority over your own family? | **.861** | .034 | .004 | .008 |
| *...you had the reputation of being someone without sexual experience?* | *.125* | *.184* | *-.118* | *-.084* |
| *...you were known as someone who cannot support a family?* | *.121* | *.415* | *.227* | *.064* |
| ...your family had a bad reputation? | -.004 | **.680** | .017 | .149 |
| ...you let other people insult your family? | -.008 | **.564** | .265 | -.020 |
| ...you did something to damage your family’s reputation? | .002 | **.537** | .307 | -.003 |
| ...your sister or mother had the reputation of sleeping around? | .030 | **.470** | -.110 | .430 |
| ...you lied to others? | .018 | -.120 | **.722** | .057 |
| ...you did not keep your word? | -.010 | -.005 | **.676** | .026 |
| ...you were hypocritical? | .003 | .096 | **.648** | -.026 |
| ...you had the reputation of being dishonest with others? | .010 | .155 | **.614** | -.022 |
| ...you were known as someone who has had many different sexual partners? | -.003 | .014 | -.049 | **.901** |
| ...you slept with someone without starting a serious relationship with that person? | .040 | -.092 | .033 | **.798** |
| ...you were known as someone whom it is easy to sleep with? | -.066 | .145 | .059 | **.725** |
| ...you changed boyfriend/girlfriend often? | .039 | .019 | .195 | **.515** |
| *Note.* Shown are the standardized loadings for the final four-factor solution of the exploratory factor analysis conducted with the personal endorsement honor concern items (sorted by primary loading strength on each factor). Italicized items were excluded at this step from future analyses. | | | | |

## Table S11

#### Confirmatory Factor Analysis Loadings of Own Concern Items (Six-Factor Solution) (Study 1)

| **Item** | **Family Reputation** | **Sexual Propriety** | **Family authority** | **Integrity** | **Face** | **Dignity** |
| --- | --- | --- | --- | --- | --- | --- |
| ...your sister or mother had the reputation of sleeping around? | .721 |  |  |  |  |  |
| ...your family had a bad reputation? | .534 |  |  |  |  |  |
| ...you did something to damage your family’s reputation? | .265 |  |  |  |  |  |
| ...you let other people insult your family? | .264 |  |  |  |  |  |
| ...you were known as someone who has had many different sexual partners? |  | .835 |  |  |  |  |
| ...you slept with someone without starting a serious relationship with that person? |  | .711 |  |  |  |  |
| ...you were known as someone whom it is easy to sleep with? |  | .705 |  |  |  |  |
| ...you changed boyfriend/girlfriend often? |  | .493 |  |  |  |  |
| ...you were known as someone who lacks authority over your own family? |  |  | .827 |  |  |  |
| ...you lacked authority over your own family? |  |  | .744 |  |  |  |
| ...you lied to others? |  |  |  | .556 |  |  |
| ...you did not keep your word? |  |  |  | .350 |  |  |
| ...you were hypocritical? |  |  |  | .326 |  |  |
| ...you had the reputation of being dishonest with others? |  |  |  | .266 |  |  |
| ...you criticized somebody else in public? |  |  |  |  | .424 |  |
| ...you caused conflict in your social relationships? |  |  |  |  | .343 |  |
| ...you failed to show humility about your achievements? |  |  |  |  | .297 |  |
| ...you lost control over your behavior in front of others? |  |  |  |  | .271 |  |
| ...you made decisions based on others’ opinions and not your own? |  |  |  |  |  | .423 |
| ...you were not true to yourself? |  |  |  |  |  | .412 |
| ...you did not stand up for what you believe? |  |  |  |  |  | .402 |
| ...you cared about what others think of you more than your own thoughts? |  |  |  |  |  | .385 |
| *Note.* Shown are the standardized loadings for the final six-factor solution of the within-samples confirmatory factor analysis conducted with the personal endorsement concern items (sorted by primary loading strength on each factor). | | | | | | |

## Table S12

#### Multilevel Confirmatory Factor Analysis Loadings of Personal Concern Items (Study 1)

|  | **Within-Samples (Level 1)** | | | | | |  | **Between-Samples (Level 2)** | | | |
| --- | --- | --- | --- | --- | --- | --- | --- | --- | --- | --- | --- |
| **Item** | **Family Reputation** | **Sexual Propriety** | **Family Authority** | **Integrity** | **Face** | **Dignity** |  | **Family Reputation** | **Sexual Propriety** | **Family Authority** | **Dignity** |
| ...your sister or mother had the reputation of sleeping around? | .687 |  |  |  |  |  |  | .899 |  |  |  |
| ...your family had a bad reputation? | .530 |  |  |  |  |  |  | .602 |  |  |  |
| ...you did something to damage your family’s reputation? | .339 |  |  |  |  |  |  | - |  |  |  |
| ...you let other people insult your family? | .278 |  |  |  |  |  |  | - |  |  |  |
| ...you were known as someone who has had many different sexual partners? |  | .787 |  |  |  |  |  |  | .911 |  |  |
| ...you slept with someone without starting a serious relationship with that person? |  | .633 |  |  |  |  |  |  | .870 |  |  |
| ...you were known as someone whom it is easy to sleep with? |  | .685 |  |  |  |  |  |  | .748 |  |  |
| ...you changed boyfriend/girlfriend often? |  | .516 |  |  |  |  |  |  | .411 |  |  |
| ...you lacked authority over your own family? |  |  | .781 |  |  |  |  |  |  | .873 |  |
| ...you were known as someone who lacks authority over your own family? |  |  | .770 |  |  |  |  |  |  | .871 |  |
| ...you had the reputation of being dishonest with others? |  |  |  | .284 |  |  |  |  |  |  |  |
| ...you lied to others? |  |  |  | .535 |  |  |  |  |  |  |  |
| ...you were hypocritical? |  |  |  | .373 |  |  |  |  |  |  |  |
| ...you did not keep your word? |  |  |  | .389 |  |  |  |  |  |  |  |
| ...you criticized somebody else in public? |  |  |  |  | .447 |  |  |  |  |  |  |
| ...you caused conflict in your social relationships? |  |  |  |  | .345 |  |  |  |  |  |  |
| ...you lost control over your behavior in front of others? |  |  |  |  | .251 |  |  |  |  |  |  |
| ...you failed to show humility about your achievements? |  |  |  |  | .369 |  |  |  |  |  |  |
| ...you cared about what others think of you more than your own thoughts? |  |  |  |  |  | .342 |  |  |  |  | .737 |
| ...you made decisions based on others’ opinions and not your own? |  |  |  |  |  | .371 |  |  |  |  | .727 |
| ...you did not stand up for what you believe? |  |  |  |  |  | .391 |  |  |  |  | .681 |
| ...you were not true to yourself? |  |  |  |  |  | .389 |  |  |  |  | .680 |
| *Note.* Shown are the standardized loadings for the final solution of the multilevel confirmatory factor analysis conducted with the personal endorsement concern items (with six factors at the within-samples and four factors at the between-samples level ; items are sorted by primary loading strength on sample-level factors). | | | | | | | | | | | |

## Table S13

#### Means, standard deviations, and correlations (Study 1)

|  | Variable |  | *M* | *SD* | 1 | 2 | 3 | 4 | 5 | 6 | 7 | 8 | 9 | 10 | 11 | 12 | 13 | 14 | 15 |
| --- | --- | --- | --- | --- | --- | --- | --- | --- | --- | --- | --- | --- | --- | --- | --- | --- | --- | --- | --- |
| **Perceived Normative Values** | | |  |  |  |  |  |  |  |  |  |  |  |  |  |  |  |  |  |
| 1 | Dignity |  | 0.01 | 0.28 |  |  |  |  |  |  |  |  |  |  |  |  |  |  |  |
| 2 | Face |  | -0.01 | 0.23 | -.93** |  |  |  |  |  |  |  |  |  |  |  |  |  |  |
| 3 | Honor |  | 0.02 | 0.43 | -.04* | -.14** |  |  |  |  |  |  |  |  |  |  |  |  |  |
| **Perceived Normative Concerns** | | |  |  |  |  |  |  |  |  |  |  |  |  |  |  |  |  |  |
| 4 | Dignity |  | 0.05 | 0.31 | .55** | -.45** | .30** |  |  |  |  |  |  |  |  |  |  |  |  |
| 5 | Face |  | -0.00 | 0.35 | -.38** | .56** | -.26** | .02 |  |  |  |  |  |  |  |  |  |  |  |
| 6 | Family Reputation |  | 0.04 | 0.39 | -.17** | -.03 | .75** | .22** | -.10** |  |  |  |  |  |  |  |  |  |  |
| 7 | Sexual Propriety |  | 0.03 | 0.74 | -.59** | .55** | .43** | -.06** | .47** | .60** |  |  |  |  |  |  |  |  |  |
| 8 | Family Authority |  | 0.05 | 0.43 | -.02 | -.05** | .70** | .44** | -.37** | .57** | .44** |  |  |  |  |  |  |  |  |
| 9 | Integrity |  | 0.01 | 0.26 | .04* | .22** | -.37** | .41** | .81** | -.36** | -.01 | -.35** |  |  |  |  |  |  |  |
| **Personal Values** | |  |  |  |  |  |  |  |  |  |  |  |  |  |  |  |  |  |  |
| 10 | Dignity |  | 0.01 | 0.45 | .74** | -.77** | .32** | .40** | -.65** | .24** | -.21** | .44** | -.45** |  |  |  |  |  |  |
| 11 | Face |  | -0.05 | 0.47 | -.12** | .10** | .67** | .01 | .06** | .25** | .38** | .34** | -.09** | .10** |  |  |  |  |  |
| 12 | Honor |  | -0.06 | 0.55 | -.28** | .24** | .61** | -.06** | .11** | .27** | .43** | .36** | -.06** | -.02 | .88** |  |  |  |  |
| **Personal Concerns** | |  |  |  |  |  |  |  |  |  |  |  |  |  |  |  |  |  |  |
| 13 | Dignity |  | 0.06 | 0.36 | .46** | -.41** | .07** | .57** | -.54** | -.03 | -.49** | .38** | -.08** | .44** | -.29** | -.41** |  |  |  |
| 14 | Family Reputation |  | 0.01 | 0.77 | -.61** | .60** | .19** | -.10** | .45** | .14** | .38** | .08** | .32** | -.57** | .25** | .58** | -.36** |  |  |
| 15 | Sexual Propriety |  | -0.01 | 0.97 | -.65** | .73** | .05** | -.17** | .69** | .03 | .73** | .12** | .37** | -.56** | .33** | .51** | -.60** | .68** |  |
| 16 | Family Authority |  | 0.08 | 0.68 | -.13** | .27** | -.00 | .37** | -.03 | -.18** | -.05** | .43** | .26** | -.12** | -.11** | -.03 | .59** | .32** | .20** |

*Note.* N = 22 cultural samples. *M* and *SD* are used to represent mean and standard deviation, respectively. * *p* < .05. ** *p* < .01.

## Table S14

#### Pairwise Comparisons of Perceived Normative Dignity, Face, and Honor Values between 5 Regions (Study 1)

| **Region 1** | **Region 2** | **Dignity** | | | |  | **Face** | | | |  | **Honor** | | | |
| --- | --- | --- | --- | --- | --- | --- | --- | --- | --- | --- | --- | --- | --- | --- | --- |
|  |  | ***b*** | ***SE*** | ***p*** | ***d*** |  | ***b*** | ***SE*** | ***p*** | ***d*** |  | ***b*** | ***SE*** | ***p*** | ***d*** |
| Anglo-Western | Latin  Europe | **-0.409** | **0.090** | **< .001** | **-0.909** |  | -0.133 | 0.155 | .388 | -0.404 |  | 0.068 | 0.092 | .457 | 0.102 |
|  | Southeast Europe | **-0.359** | **0.060** | **< .001** | **-0.798** |  | -0.155 | 0.141 | .273 | -0.469 |  | **0.298** | **0.069** | **< .001** | **0.447** |
|  | MENA | **-0.547** | **0.042** | **< .001** | **-1.214** |  | 0.146 | 0.173 | .400 | 0.443 |  | **1.036** | **0.091** | **< .001** | **1.550** |
|  | East Asia | **-1.063** | **0.034** | **< .001** | **-2.359** |  | **0.438** | **0.149** | **.003** | **1.327** |  | -0.217 | 0.121 | .074 | -0.324 |
| Latin  Europe | Southeast Europe | 0.050 | 0.105 | .634 | 0.111 |  | -0.022 | 0.093 | .814 | -0.066 |  | **0.230** | **0.083** | **.006** | **0.344** |
|  | MENA | -0.137 | 0.096 | .154 | -0.305 |  | 0.279 | 0.140 | .045 | 0.847 |  | **0.967** | **0.100** | **< .001** | **1.447** |
|  | East Asia | **-0.654** | **0.094** | **< .001** | **-1.451** |  | **0.570** | **0.105** | **< .001** | **1.730** |  | -0.286 | 0.130 | .028 | -0.427 |
| Southeast Europe | MENA | **-0.187** | **0.071** | **.008** | **-0.416** |  | 0.301 | 0.125 | .016 | 0.913 |  | **0.738** | **0.080** | **< .001** | **1.103** |
|  | East Asia | **-0.704** | **0.066** | **< .001** | **-1.562** |  | **0.591** | **0.083** | **< .001** | **1.795** |  | **-0.516** | **0.117** | **< .001** | **-0.771** |
| MENA | East Asia | **-0.516** | **0.050** | **< .001** | **-1.146** |  | 0.291 | 0.135 | .032 | 0.884 |  | **-1.253** | **0.125** | **< .001** | **-1.874** |

*Note*. Estimates represent the unstandardized estimates for the dummy coded-region variables added to the final measurement models. All possible combinations were obtained by recoding the reference group. Effect sizes were obtained from a model in which the outcome variable, but not the predictors, were standardized in MPLUS (STDY model). Bolded rows indicate comparisons that are significant following Holm-Bonferroni correction. *Anglo-Western*: United States, United Kingdom; *Latin Europe*: Spain, Italy; *Southeast Europe*: Greece, Greek Cypriot community; *MENA*: Turkey, Lebanon, Egypt; *East Asia*: South Korea, Japan.

## Table S15

#### Pairwise Comparisons of Perceived Normative Dignity, Face, and Honor Concerns between 5 Regions (Study 1)

| **Region 1** | **Region 2** | **Dignity** | | | |  | **Face** | | | |  | **Family Reputation** | | | |  | **Family Authority** | | | |  | **Sexual Propriety** | | | |  | **Integrity** | | | |
| --- | --- | --- | --- | --- | --- | --- | --- | --- | --- | --- | --- | --- | --- | --- | --- | --- | --- | --- | --- | --- | --- | --- | --- | --- | --- | --- | --- | --- | --- | --- |
|  |  | ***b*** | ***SE*** | ***p*** | ***d*** |  | ***b*** | ***SE*** | ***p*** | ***d*** |  | ***b*** | ***SE*** | ***p*** | ***d*** |  | ***b*** | ***SE*** | ***p*** | ***d*** |  | ***b*** | ***SE*** | ***p*** | ***d*** |  | ***b*** | ***SE*** | ***p*** | ***d*** |
| Anglo-Western | Latin Europe | 0.044 | 0.093 | .637 | 0.103 |  | -0.164 | 0.213 | .442 | -0.362 |  | 0.149 | 0.162 | .358 | 0.262 |  | 0.074 | 0.184 | .687 | 0.118 |  | -0.200 | 0.205 | .327 | -0.203 |  | -0.117 | 0.181 | .518 | -0.396 |
|  | Southeast Europe | **0.353** | **0.094** | **< .001** | **0.832** |  | 0.068 | 0.174 | .697 | 0.149 |  | **0.737** | **0.151** | **< .001** | **1.293** |  | **0.559** | **0.130** | **< .001** | **0.889** |  | 0.405 | 0.263 | .123 | 0.410 |  | 0.017 | 0.069 | .806 | 0.057 |
|  | MENA | 0.127 | 0.177 | .472 | 0.299 |  | -0.032 | 0.255 | .901 | -0.070 |  | **0.958** | **0.114** | **< .001** | **1.680** |  | **0.971** | **0.141** | **< .001** | **1.545** |  | **1.374** | **0.268** | **< .001** | **1.389** |  | -0.156 | 0.225 | .488 | -0.527 |
|  | East Asia | **-0.488** | **0.113** | **< .001** | **-1.148** |  | 0.604 | 0.299 | .044 | 1.333 |  | 0.442 | 0.286 | .123 | 0.775 |  | 0.041 | 0.219 | .852 | 0.065 |  | **1.224** | **0.251** | **< .001** | **1.237** |  | 0.060 | 0.137 | .663 | 0.202 |
| Latin  Europe | Southeast Europe | **0.309** | **0.076** | **< .001** | **0.728** |  | 0.232 | 0.165 | .160 | 0.512 |  | **0.588** | **0.190** | **.002** | **1.032** |  | **0.485** | **0.162** | **.003** | **0.772** |  | 0.605 | 0.260 | .020 | 0.612 |  | 0.134 | 0.170 | .429 | 0.454 |
|  | MENA | 0.083 | 0.167 | .620 | 0.196 |  | 0.133 | 0.252 | .599 | 0.293 |  | **0.809** | **0.161** | **< .001** | **1.419** |  | **0.897** | **0.167** | **< .001** | **1.428** |  | **1.574** | **0.267** | **< .001** | **1.592** |  | -0.038 | 0.274 | .889 | -0.130 |
|  | East Asia | **-0.531** | **0.096** | **< .001** | **-1.251** |  | 0.768 | 0.296 | .009 | 1.696 |  | 0.293 | 0.308 | .341 | 0.514 |  | -0.033 | 0.237 | .889 | -0.053 |  | **1.424** | **0.249** | **< .001** | **1.440** |  | 0.177 | 0.210 | .399 | 0.599 |
| Southeast Europe | MENA | -0.226 | 0.166 | .173 | -0.533 |  | -0.099 | 0.223 | .656 | -0.219 |  | 0.221 | 0.147 | .134 | 0.387 |  | **0.413** | **0.105** | **< .001** | **0.657** |  | **0.969** | **0.314** | **.002** | **0.980** |  | -0.172 | 0.220 | .433 | -0.582 |
|  | East Asia | **-0.841** | **0.098** | **< .001** | **-1.980** |  | 0.538 | 0.271 | .047 | 1.186 |  | -0.294 | 0.303 | .332 | -0.515 |  | **-0.516** | **0.196** | **.009** | **-0.822** |  | **0.820** | **0.299** | **.006** | **0.829** |  | 0.044 | 0.126 | .724 | 0.150 |
| MENA | East Asia | **-0.615** | **0.179** | **.001** | **-1.447** |  | 0.636 | 0.326 | .051† | 1.404 |  | -0.515 | 0.286 | .072 | -0.904 |  | **-0.930** | **0.205** | **< .001** | **-1.480** |  | -0.150 | 0.304 | .621 | -0.152 |  | 0.216 | 0.249 | .386 | 0.729 |

*Note*. Estimates represent the unstandardized estimates for the dummy coded-region variables added to the final measurement models. All possible combinations were obtained by recoding the reference group. Effect sizes were obtained from a model in which the outcome variable, but not the predictors, were standardized in MPLUS (STDY model). Bolded rows indicate comparisons that are significant following Holm-Bonferroni correction. *Anglo-Western*: United States, United Kingdom; *Latin Europe*: Spain, Italy; *Southeast Europe*: Greece, Greek Cypriot community; *MENA*: Turkey, Lebanon, Egypt; *East Asia*: South Korea, Japan.

## Table S16

#### Pairwise Comparisons of Personal Dignity, Face, and Honor Values between 5 Regions (Study 1)

| **Region 1** | **Region 2** | **Dignity** | | | |  | **Face** | | | |  | **Honor** | | | |  |
| --- | --- | --- | --- | --- | --- | --- | --- | --- | --- | --- | --- | --- | --- | --- | --- | --- |
|  |  | ***b*** | ***SE*** | ***p*** | ***d*** |  | ***b*** | ***SE*** | ***p*** | ***d*** |  | ***b*** | ***SE*** | ***P*** | ***d*** |  |
| Anglo-Western | Latin Europe | -0.556 | 0.248 | .025 | -0.865 |  | 0.026 | 0.154 | .865 | 0.040 |  | -0.427 | 0.221 | .053 | -0.549 |  |
|  | Southeast Europe | **-0.282** | **0.051** | **< .001** | **-0.438** |  | -0.452 | 0.259 | .080 | -0.685 |  | -0.169 | 0.230 | .463 | -0.217 |  |
|  | MENA | -0.337 | 0.153 | .028 | -0.523 |  | **0.759** | **0.162** | **< .001** | **1.149** |  | **0.904** | **0.256** | **< .001** | **1.163** |  |
|  | East Asia | **-1.275** | **0.099** | **< .001** | **-1.981** |  | -0.192 | 0.154 | .211 | -0.291 |  | 0.194 | 0.172 | .258 | 0.250 |  |
| Latin  Europe | Southeast Europe | 0.274 | 0.245 | .262 | 0.427 |  | -0.478 | 0.256 | .062 | -0.724 |  | 0.258 | 0.266 | .332 | 0.332 |  |
|  | MENA | 0.220 | 0.284 | .439 | 0.341 |  | **0.733** | **0.156** | **< .001** | **1.109** |  | **1.331** | **0.289** | **< .001** | **1.711** |  |
|  | East Asia | **-0.718** | **0.258** | **.005** | **-1.117** |  | -0.219 | 0.150 | .145 | -0.331 |  | **0.621** | **0.217** | **.004** | **0.799** |  |
| Southeast  Europe | MENA | -0.055 | 0.146 | .708 | -0.085 |  | **1.211** | **0.260** | **< .001** | **1.833** |  | **1.073** | **0.296** | **< .001** | **1.380** |  |
|  | East Asia | **-0.993** | **0.088** | **< .001** | **-1.543** |  | 0.260 | 0.257 | .312 | 0.393 |  | 0.363 | 0.226 | .109 | 0.467 |  |
| MENA | East Asia | **-0.938** | **0.169** | **< .001** | **-1.458** |  | **-0.951** | **0.157** | **< .001** | **-1.440** |  | **-0.710** | **0.253** | **.005** | **-0.913** |  |

*Note*. Estimates represent the unstandardized estimates for the dummy coded-region variables added to the final measurement models. All possible combinations were obtained by recoding the reference group. Effect sizes were obtained from a model in which the outcome variable, but not the predictors, were standardized in MPLUS (STDY model). Bolded rows indicate comparisons that are significant following Holm-Bonferroni correction. *Anglo-Western*: United States, United Kingdom; *Latin Europe*: Spain, Italy; *Southeast Europe*: Greece, Greek Cypriot community; *MENA*: Turkey, Lebanon, Egypt; *East Asia*: South Korea, Japan.

## Table S17

#### Pairwise Comparisons of Personal Dignity, Face, and Honor Concerns between 5 Regions (Study 1)

| **Region 1** | **Region 2** | **Dignity** | | | |  | **Family Reputation** | | | |  | **Family Authority** | | | |  | **Sexual Propriety** | | | |
| --- | --- | --- | --- | --- | --- | --- | --- | --- | --- | --- | --- | --- | --- | --- | --- | --- | --- | --- | --- | --- |
|  |  | ***b*** | ***SE*** | ***p*** | ***d*** |  | ***b*** | ***SE*** | ***p*** | ***d*** |  | ***b*** | ***SE*** | ***p*** | ***d*** |  | ***b*** | ***SE*** | ***p*** | ***d*** |
| Anglo-Western | Latin  Europe | 0.305 | 0.204 | .135 | 0.620 |  | -0.246 | 0.535 | .646 | -0.229 |  | 0.154 | 0.360 | .669 | 0.199 |  | -0.659 | 0.418 | .115 | -0.525 |
|  | Southeast Europe | 0.359 | 0.167 | .032 | 0.730 |  | 0.900 | 0.393 | .022 | 0.838 |  | 0.600 | 0.380 | .114 | 0.778 |  | 0.008 | 0.321 | .980 | 0.006 |
|  | MENA | 0.046 | 0.192 | .811 | 0.094 |  | **1.169** | **0.421** | **.006** | **1.089** |  | 0.298 | 0.560 | .595 | 0.386 |  | **0.963** | **0.354** | **.006** | **0.768** |
|  | East Asia | -0.495 | 0.201 | .014 | -1.005 |  | **1.513** | **0.343** | **< .001** | **1.410** |  | 0.104 | 0.592 | .860 | 0.135 |  | **1.599** | **0.356** | **< .001** | **1.273** |
| Latin  Europe | Southeast Europe | 0.054 | 0.233 | .816 | 0.110 |  | 1.145 | 0.460 | .013 | 1.067 |  | 0.447 | 0.203 | .028 | 0.579 |  | 0.667 | 0.474 | .160 | 0.531 |
|  | MENA | -0.259 | 0.252 | .303 | -0.526 |  | **1.415** | **0.483** | **.003** | **1.318** |  | 0.144 | 0.458 | .754 | 0.186 |  | **1.622** | **0.496** | **.001** | **1.292** |
|  | East Asia | **-0.800** | **0.258** | **.002** | **-1.624** |  | **1.759** | **0.417** | **< .001** | **1.639** |  | -0.049 | 0.497 | .921 | -0.064 |  | **2.257** | **0.499** | **< .001** | **1.798** |
| Southeast Europe | MENA | -0.313 | 0.221 | .156 | -0.636 |  | 0.270 | 0.316 | .394 | 0.251 |  | -0.303 | 0.476 | .524 | -0.393 |  | 0.956 | 0.406 | .019 | 0.761 |
|  | East Asia | **-0.854** | **0.231** | **< .001** | **-1.734** |  | **0.613** | **0.206** | **.003** | **0.572** |  | -0.496 | 0.513 | .333 | -0.643 |  | **1.591** | **0.421** | **< .001** | **1.267** |
| MENA | East Asia | -0.541 | 0.249 | .030 | -1.098 |  | 0.344 | 0.255 | .177 | 0.320 |  | -0.193 | 0.657 | .769 | -0.250 |  | 0.635 | 0.446 | .154 | 0.506 |

*Note*. Estimates represent the unstandardized estimates for the dummy coded-region variables added to the final measurement models. All possible combinations were obtained by recoding the reference group. Effect sizes were obtained from a model in which the outcome variable, but not the predictors, were standardized in MPLUS (STDY model). Bolded rows indicate comparisons that are significant following Holm-Bonferroni correction. *Anglo-Western*: United States, United Kingdom; *Latin Europe*: Spain, Italy; *Southeast Europe*: Greece, Greek Cypriot community; *MENA*: Turkey, Lebanon, Egypt; *East Asia*: South Korea, Japan.

## Table S18

#### Pairwise Comparisons of Cultural Values and Concerns between Genders (Study 1)

| **Outcome** | | **Women**  *(compared to men as the reference group)* | | | |
| --- | --- | --- | --- | --- | --- |
|  | | ***b*** | ***SE*** | ***p*** | ***d*** |
| **Perceived Normative Values** | |  |  |  |  |
|  | Dignity | -0.035 | 0.146 | .809 | -0.115 |
|  | Face | -0.085 | 0.125 | .494 | -0.330 |
|  | Honor | -0.151 | 0.207 | .464 | -0.315 |
| **Perceived Normative Concerns** | |  |  |  |  |
|  | Dignity | 0.110 | 0.149 | .460 | 0.327 |
|  | Face | 0.259 | 0.188 | .167 | 0.655 |
|  | Family Reputation | 0.129 | 0.204 | .525 | 0.294 |
|  | Sexual Propriety | 0.761 | 0.286 | .008 | 0.909 |
|  | Family Authority | -0.069 | 0.207 | .738 | -0.144 |
|  | Integrity | 0.026 | 0.143 | .856 | 0.092 |
| **Personal Values** | |  |  |  |  |
|  | Dignity | -0.017 | 0.215 | .937 | -0.034 |
|  | Face | -0.192 | 0.222 | .387 | -0.382 |
|  | Honor | -0.448 | 0.250 | .073 | -0.694 |
| **Personal Concerns** | |  |  |  |  |
|  | Dignity | -0.202 | 0.187 | .281 | -0.491 |
|  | Family Reputation | -0.625 | 0.352 | .076 | -0.623 |
|  | Sexual Propriety | 0.450 | 0.420 | .284 | -0.695 |
|  | Family Authority | -0.489 | 0.326 | .134 | 0.446 |
| *Note*. Estimates represent the unstandardized estimates for the dummy coded gender variables added to the final measurement models. Effect sizes were obtained from a model in which the outcome variable, but not the predictors, were standardized in MPLUS (STDY model). | | | | | |

## Table S19

#### Confirmatory Factor Analysis Loadings of Perceived Normative Honor Values (Two-Factor Solution) (Study 2)

| **Item** | **Defense of  Family Reputation** | **Self-Promotion &  Retaliation** |
| --- | --- | --- |
| People should be concerned about defending their families’ reputation. | .734 |  |
| People should be concerned about damaging their families’ reputation. | .707 |  |
| People should be concerned about their family having a bad reputation. | .684 |  |
| People should not allow others to insult their family. | .629 |  |
| Men need to protect their women’s reputation at all costs. | .607 |  |
| You must punish people who insult you. |  | .681 |
| People always need to show off their power in front of their competitors. |  | .641 |
| If a person gets insulted and they don’t respond, he or she will look weak. |  | .577 |
| *Note.* Shown are the standardized loadings for the final four-factor solution of the within-samples confirmatory factor analysis conducted with the perceived normative value items (sorted by primary loading strength on each factor). | | |

## Table S20

#### Multilevel Confirmatory Factor Analysis Loadings of Perceived Normative Value Items (Study 2)

| **Item** | **Within-Samples (Level 1)** | |  | **Between-Samples (Level 2)** |
| --- | --- | --- | --- | --- |
|  | **Defense of  Family Reputation** | **Self-Promotion & Retaliation** |  | **Honor** |
| People should be concerned about defending their families’ reputation. | .714 |  |  | .959 |
| Men need to protect their women’s reputation at all costs. | .565 |  |  | .943 |
| People should be concerned about damaging their families’ reputation. | .703 |  |  | .934 |
| People always need to show off their power in front of their competitors. |  | .611 |  | .929 |
| People should be concerned about their family having a bad reputation. | .669 |  |  | .883 |
| You must punish people who insult you. |  | .649 |  | .842 |
| People should not allow others to insult their family. | .626 |  |  | .837 |
| If a person gets insulted and they don’t respond, he or she will look weak. |  | .596 |  | .557 |

*Note.* Shown are the standardized loadings for the final solution of the multilevel confirmatory factor analysis conducted with the personal value items (with four factors at the within-samples and three factors at the sample-level; items are sorted by primary loading strength on sample-level factor).

## Table S21

#### Confirmatory Factor Analysis Loadings of Personal Honor Values (Two-Factor Solution) (Study 2)

| **Item** | **Defense of  Family Reputation** | **Self-Promotion &  Retaliation** |
| --- | --- | --- |
| People should be concerned about defending their families’ reputation. | .754 |  |
| People should be concerned about damaging their families’ reputation. | .712 |  |
| People should be concerned about their family having a bad reputation. | .685 |  |
| Men need to protect their women’s reputation at all costs. | .617 |  |
| People should not allow others to insult their family. | .601 |  |
| You must punish people who insult you. |  | .710 |
| People always need to show off their power in front of their competitors. |  | .599 |
| If a person gets insulted and they don’t respond, he or she will look weak. |  | .527 |
| *Note.* Shown are the standardized loadings for the final four-factor solution of the within-samples confirmatory factor analysis conducted with the perceived normative value items (sorted by primary loading strength on each factor). | | |

## Table S22

#### Multilevel Confirmatory Factor Analysis Loadings of Personal Value Items (Study 2)

| **Item** | **Within-Samples (Level 1)** | |  | **Between-Samples (Level 2)** |
| --- | --- | --- | --- | --- |
|  | **Defense of  Family Reputation** | **Self-Promotion & Retaliation** |  | **Honor** |
| People should be concerned about defending their families’ reputation. | .726 |  |  | .968 |
| Men need to protect their women’s reputation at all costs. | .564 |  |  | .941 |
| People always need to show off their power in front of their competitors. |  | .563 |  | .925 |
| People should be concerned about damaging their families’ reputation. | .709 |  |  | .909 |
| People should be concerned about their family having a bad reputation. | .670 |  |  | .853 |
| You must punish people who insult you. |  | .656 |  | .820 |
| People should not allow others to insult their family. | .597 |  |  | .742 |
| If a person gets insulted and they don’t respond, he or she will look weak. |  | .556 |  | .459 |

*Note.* Shown are the standardized loadings for the final solution of the multilevel confirmatory factor analysis conducted with the personal value items (with four factors at the within-samples and three factors at the sample-level; items are sorted by primary loading strength on sample-level factor).

## Table S23

#### Means, standard deviations, and correlation (Study 2)

| Variable | *M* | *SD* | *r* |
| --- | --- | --- | --- |
| **Honor Values** |  |  |  |
| 1. Personal Honor | 0.01 | 0.42 |  |
| 2. Perceived Normative Honor | -0.00 | 0.36 | .92** |
| *Note.* *N* = 22 cultural samples. *M* and *SD* are used to represent mean and standard deviation, respectively.  ** *p* < .01 | | | |

## Table S24

#### Pairwise Comparisons of Perceived Normative and Personal Honor Values between 5 Regions (Study 2)

| **Region 1** | **Region 2** | **Perceived Normative Honor** | | | |  | **Personal Honor** | | | |
| --- | --- | --- | --- | --- | --- | --- | --- | --- | --- | --- |
|  |  | **b** | **SE** | **p** | **d** |  | **b** | **SE** | **p** | **d** |
| Anglo-Western | Latin  Europe | 0.197 | 0.086 | .021 | 0.403 |  | 0.199 | 0.123 | .105 | 0.352 |
|  | Southeast Europe | **0.366** | **0.067** | **< .001** | **0.749** |  | 0.068 | 0.111 | .540 | 0.121 |
|  | MENA | **0.793** | **0.097** | **< .001** | **1.620** |  | **0.871** | **0.128** | **< .001** | **1.546** |
|  | East Asia | 0.107 | 0.071 | .130 | 0.219 |  | **0.306** | **0.096** | **.002** | **0.543** |
| Latin  Europe | Southeast Europe | 0.169 | 0.086 | .050 | 0.346 |  | -0.130 | 0.123 | .291 | -0.232 |
|  | MENA | **0.596** | **0.112** | **< .001** | **1.217** |  | **0.673** | **0.140** | **< .001** | **1.194** |
|  | East Asia | -0.090 | 0.090 | .315 | -0.184 |  | 0.107 | 0.111 | .332 | 0.191 |
| Southeast Europe | MENA | **0.426** | **0.098** | **< .001** | **0.871** |  | **0.803** | **0.129** | **< .001** | **1.425** |
|  | East Asia | **-0.259** | **0.072** | **< .001** | -0.530 |  | 0.238 | 0.098 | .015 | 0.422 |
| MENA | East Asia | **-0.686** | **0.101** | **< .001** | **-1.401** |  | **-0.565** | **0.117** | **< .001** | **-1.003** |

*Note*. Estimates represent the unstandardized estimates for the dummy coded-region variables added to the final measurement models. All possible combinations were obtained by recoding the reference group. Effect sizes were obtained from a model in which the outcome variable, but not the predictors, were standardized in MPLUS (STDY model). Bolded rows indicate comparisons that are significant following Holm-Bonferroni correction. *Anglo-Western*: United States, Canada, United Kingdom; *Latin Europe*: Spain, Italy; *Southeast Europe*: Greece, Greek Cypriot community; *MENA*: Turkey, Turkish Cypriot Community, Lebanon, Egypt, Tunisia; *East Asia*: South Korea, Japan.

## Table S25

#### Pairwise Comparisons of Cultural Values and Concerns between Genders (Study 2)

| **Outcome** | **Women**  *(compared to men as the reference group)* | | | |
| --- | --- | --- | --- | --- |
|  | ***b*** | ***SE*** | ***p*** | ***d*** |
| Perceived Normative Honor Values | -0.125 | 0.141 | .375 | -0.338 |
| Personal Honor Values | -0.217 | 0.161 | .176 | -0.495 |
| *Note*. Estimates represent the unstandardized estimates for the dummy coded gender variables added to the final measurement models. Effect sizes were obtained from a model in which the outcome variable, but not the predictors, were standardized in MPLUS (STDY model). | | | | |

## Table S26

#### Zero-Order Correlations of Within-Samples Variation in Perceived Normative and Personally Endorsed Honor Values with Age (Study 2)

| **Cultural Samples** | **Perceived Normative Honor Values** | | **Personal Honor Values** | |
| --- | --- | --- | --- | --- |
|  | **Defense of  Family Reputation** | **Self-Promotion  & Retaliation** | **Defense of  Family Reputation** | **Self-Promotion  & Retaliation** |
| Canada - Female | .11 [-.03, .24] | -.10 [-.23, .04] | .09 [-.04, .22] | -.23*** [-.35, -.10] |
| Canada - Male | .24*** [ .10, .36] | -.13 [-.27, .01] | .21** [ .07, .34] | -.13 [-.26, .01] |
| Cyprus North - Female | .12 [-.02, .26] | .12 [-.02, .26] | -.07 [-.21, .08] | -.07 [-.21, .08] |
| Cyprus North - Male | .19** [ .06, .31] | .12 [-.02, .25] | .07 [-.06, .21] | -.03 [-.16, .11] |
| Cyprus South - Female | .02 [-.15, .19] | -.07 [-.24, .10] | .14 [-.03, .30] | -.09 [-.25, .08] |
| Cyprus South - Male | .16* [ .00, .32] | .00 [-.16, .16] | .32*** [ .16, .45] | .04 [-.12, .20] |
| Egypt - Female | .15* [ .01, .29] | .13 [-.01, .26] | .13 [-.01, .27] | .04 [-.11, .17] |
| Egypt - Male | .16* [ .02, .29] | .09 [-.05, .23] | .27*** [ .14, .39] | .14* [ .00, .27] |
| Greece - Female | .15* [ .02, .29] | .10 [-.04, .24] | .30*** [ .17, .42] | .17* [ .03, .30] |
| Greece - Male | .24*** [ .10, .37] | .11 [-.02, .25] | .33*** [ .20, .45] | .07 [-.07, .21] |
| Italy - Female | .12 [-.02, .26] | .01 [-.13, .15] | .23*** [ .10, .36] | .03 [-.11, .17] |
| Italy - Male | .22** [ .09, .35] | -.04 [-.18, .10] | .24*** [ .10, .36] | -.13 [-.27, .00] |
| Japan - Female | .23** [ .09, .35] | .05 [-.09, .19] | .19** [ .05, .32] | -.14* [-.27, .00] |
| Japan - Male | .12 [-.02, .26] | -.09 [-.22, .05] | .19** [ .05, .32] | -.06 [-.20, .08] |
| Korea - Female | .15* [ .01, .28] | -.09 [-.23, .05] | .09 [-.05, .23] | -.04 [-.18, .10] |
| Korea - Male | .26*** [ .12, .38] | -.08 [-.22, .06] | .32*** [ .19, .44] | .06 [-.08, .20] |
| Lebanon - Female | .22** [ .08, .35] | .15* [ .01, .28] | .2** [ .07, .33] | .05 [-.09, .19] |
| Lebanon - Male | .19** [ .05, .32] | .18** [ .05, .31] | .13 [-.01, .26] | .04 [-.10, .17] |
| Spain - Female | .28*** [ .15, .40] | -.04 [-.18, .10] | .27*** [ .13, .39] | -.01 [-.15, .13] |
| Spain - Male | .28*** [ .15, .40] | -.05 [-.19, .09] | .31*** [ .18, .43] | -.05 [-.19, .09] |
| Tunisia - Female | .07 [-.07, .21] | .06 [-.08, .20] | .01 [-.13, .15] | -.04 [-.18, .10] |
| Tunisia - Male | .11 [-.03, .25] | .02 [-.12, .16] | .25*** [ .11, .37] | .04 [-.10, .18] |
| Turkey - Female | .07 [-.07, .21] | -.06 [-.20, .08] | .04 [-.10, .18] | -.11 [-.24, .03] |
| Turkey - Male | .18* [ .04, .31] | .06 [-.08, .19] | .29*** [ .16, .42] | -.09 [-.22, .05] |
| UK - Female | .11 [-.03, .24] | -.05 [-.19, .09] | .16 [ .02, .29] | -.19** [-.32, -.05] |
| UK - Male | .25*** [ .12, .38] | -.18* [-.31, -.04] | .43*** [ .31, .53] | -.19** [-.32, -.05] |
| United States - Female | .10 [-.04, .23] | -.29** [-.41, -.16] | .08 [-.06, .22] | -.37*** [-.48, -.24] |
| United States - Male | .03 [-.11, .17] | -.37** [-.49, -.25] | .05 [-.09, .19] | -.47*** [-.57, -.35] |
| Overall (pooled samples) | .14*** [.12, .17] | -.03* [-.06, .00] | .17*** [.14, .19] | -.07*** [-.10, -.04] |

*Note*. Presented are the Pearson correlation coefficients for each cultural sample. Values in brackets represent 95% confidence intervals.
*** *p* < .001, ** *p* < .01, * *p* < .05

## Table S27

#### Multilevel Regression Parameters Testing for Regional Variation in Age Trends in Perceived Normative and Personally Endorsed Honor Values (Study 2)

| **Cultural Samples** | **Perceived Normative Honor Values** | | | |  | **Personal Honor Values** | | | |
| --- | --- | --- | --- | --- | --- | --- | --- | --- | --- |
|  | **B** | **SE** | **[95% CI]** | ***p*** |  | **B** | **SE** | **[95% CI]** | ***p*** |
| **Defense of Family Reputation** | | | | | | | | | |
|  |  |  |  |  |  |  |  |  |  |
| Pooled age trends | 0.09 | 0.01 | [ 0.07, 0.10] | <.001 |  | 0.11 | 0.01 | [ 0.10, 0.13] | <.001 |
|  |  |  |  |  |  |  |  |  |  |
| Simple slopes for age trends |  |  |  |  |  |  |  |  |  |
| Anglo-West | 0.07_a_ | 0.02 | [ 0.04, 0.10] | <.001 |  | 0.10_a_ | 0.02 | [ 0.06, 0.13] | <.001 |
| Latin Europe | 0.12_a_ | 0.02 | [ 0.08, 0.16] | <.001 |  | 0.15_ab_ | 0.02 | [ 0.11, 0.19] | <.001 |
| Southeast Europe | 0.10_a_ | 0.02 | [ 0.05, 0.14] | <.001 |  | 0.20_b_ | 0.03 | [ 0.15, 0.25] | <.001 |
| MENA | 0.11_a_ | 0.02 | [ 0.08, 0.14] | <.001 |  | 0.10_a_ | 0.02 | [ 0.07, 0.13] | <.001 |
| East Asia | 0.10_a_ | 0.02 | [ 0.06, 0.15] | <.001 |  | 0.12_ab_ | 0.02 | [ 0.07, 0.17] | <.001 |
|  |  |  |  |  |  |  |  |  |  |
| Pairwise comparisons of age trends |  |  |  |  |  |  |  |  |  |
| Anglo-West |  |  |  |  |  |  |  |  |  |
| vs. Latin Europe | 0.05 | 0.03 | [ 0.00, 0.10] | .053 |  | 0.06 | 0.03 | [ 0.00, 0.11] | .043 |
| vs. Southeast Europe | 0.02 | 0.03 | [-0.03, 0.08] | .411 |  | 0.10 | 0.03 | [ 0.04, 0.16] | <.001* |
| vs. MENA | 0.04 | 0.02 | [-0.01, 0.08] | .117 |  | 0.00 | 0.02 | [-0.04, 0.05] | .876 |
| vs. East Asia | 0.03 | 0.03 | [-0.02, 0.09] | .257 |  | 0.02 | 0.03 | [-0.04, 0.08] | .471 |
| Latin Europe |  |  |  |  |  |  |  |  |  |
| vs. Southeast Europe | -0.03 | 0.03 | [-0.09, 0.04] | .407 |  | 0.05 | 0.03 | [-0.02, 0.11] | .155 |
| vs. MENA | -0.01 | 0.03 | [-0.07, 0.04] | .594 |  | -0.05 | 0.03 | [-0.11, 0.00] | .063 |
| vs. East Asia | -0.02 | 0.03 | [-0.08, 0.04] | .559 |  | -0.03 | 0.03 | [-0.10, 0.03] | .299 |
| Southeast Europe |  |  |  |  |  |  |  |  |  |
| vs. MENA | 0.01 | 0.03 | [-0.05, 0.07] | .677 |  | -0.10 | 0.03 | [-0.16, -0.04] | .001* |
| vs. East Asia | 0.01 | 0.03 | [-0.06, 0.07] | .808 |  | -0.08 | 0.04 | [-0.15, -0.01] | .022 |
| MENA |  |  |  |  |  |  |  |  |  |
| vs. East Asia | -0.00 | 0.03 | [-0.06, 0.05] | .886 |  | 0.02 | 0.03 | [-0.04, 0.08] | .557 |
| **Self-Promotion & Retaliation** | | | | | | | | | |
|  |  | | | | | | | | |
| Pooled age trends | -0.02 | 0.01 | [-0.03, 0.00] | .034 |  | -0.04 | 0.01 | [-0.05, -0.02] | <.001 |
|  |  |  |  |  |  |  |  |  |  |
| Simple slopes for age trends |  |  |  |  |  |  |  |  |  |
| Anglo-West | -0.10_a_ | 0.02 | [-0.13, -0.07] | <.001 |  | -0.13_a_ | 0.01 | [-0.16, -0.10] | <.001 |
| Latin Europe | -0.01_b_ | 0.02 | [-0.05, 0.02] | .479 |  | -0.02_b_ | 0.02 | [-0.06, 0.02] | .279 |
| Southeast Europe | 0.02_bc_ | 0.02 | [-0.02, 0.07] | .329 |  | 0.03_b_ | 0.02 | [-0.01, 0.07] | .141 |
| MENA | 0.05_c_ | 0.02 | [ 0.02, 0.09] | <.001 |  | -0.01_b_ | 0.01 | [-0.04, 0.02] | .444 |
| East Asia | -0.03_b_ | 0.02 | [-0.07, 0.02] | .226 |  | -0.02_b_ | 0.02 | [-0.06, 0.02] | .332 |
|  |  |  |  |  |  |  |  |  |  |
| Pairwise comparisons of age trends |  |  |  |  |  |  |  |  |  |
| Anglo-West |  |  |  |  |  |  |  |  |  |
| vs. Latin Europe | 0.08 | 0.02 | [ 0.04, 0.13] | <.001* |  | 0.11 | 0.02 | [ 0.06, 0.15] | <.001* |
| vs. Southeast Europe | 0.12 | 0.03 | [ 0.07, 0.17] | <.001* |  | 0.16 | 0.03 | [ 0.11, 0.21] | <.001* |
| vs. MENA | 0.15 | 0.02 | [ 0.11, 0.20] | <.001* |  | 0.12 | 0.02 | [ 0.08, 0.16] | <.001* |
| vs. East Asia | 0.07 | 0.03 | [ 0.02, 0.12] | .008* |  | 0.11 | 0.03 | [ 0.06, 0.16] | <.001* |
| Latin Europe |  |  |  |  |  |  |  |  |  |
| vs. Southeast Europe | 0.04 | 0.03 | [-0.02, 0.10] | .229 |  | 0.05 | 0.03 | [ 0.00, 0.11] | .068 |
| vs. MENA | 0.07 | 0.02 | [ 0.02, 0.12] | .006* |  | 0.01 | 0.02 | [-0.04, 0.05] | .721 |
| vs. East Asia | -0.01 | 0.03 | [-0.07, 0.04] | .656 |  | -0.00 | 0.03 | [-0.05, 0.05] | .986 |
| Southeast Europe |  |  |  |  |  |  |  |  |  |
| vs. MENA | 0.03 | 0.03 | [-0.02, 0.09] | .253 |  | -0.04 | 0.03 | [-0.10, 0.01] | .099 |
| vs. East Asia | -0.05 | 0.03 | [-0.11, 0.01] | .123 |  | -0.05 | 0.03 | [-0.11, 0.01] | .083 |
| MENA |  |  |  |  |  |  |  |  |  |
| vs. East Asia | -0.08 | 0.03 | [-0.13, -0.03] | .003* |  | -0.01 | 0.03 | [-0.06, 0.04] | .728 |
|  |  |  |  |  |  |  |  |  |  |

*Note*. Presented are parameters from four sets of multilevel regression models predicting within-cultures factor scores for honor values as a function of age (in decades, grand-mean centered), geographical regions (dummy coded), and their cross-level interactions. Within the findings for each honor dimension, pairwise comparisons marked with a * meet a Holm-Bonferroni sequentially adjusted alpha level starting at .05 / 10 = .005, adjusting for familywise error across the 10 possible pairs of regions, and simple slopes that do not share a subscript thus differ from each other significantly, according to this Holm-Bonferroni criterion.

## Table S28

#### Further Exploratory Mediation Results for Cultural Differences in Social Cognitive Tendencies via Perceived Normative Honor Values (Study 3)

| Model Parameters | **Outcome** | | | | | | | | | | | | | | | | | | | | | | | | | | | | |
| --- | --- | --- | --- | --- | --- | --- | --- | --- | --- | --- | --- | --- | --- | --- | --- | --- | --- | --- | --- | --- | --- | --- | --- | --- | --- | --- | --- | --- | --- |
|  | **Ingroup  Closeness Bias** | | | |  | **Nepotism (Reward Situations)** | | | |  | **Nepotism  (Punishment Situations)** | | | |  | **Dispositional  Attribution Bias** | | | |  | **Inclusion of  Contextual Information** | | | |  | **Thematic  Categorization** | | | |
|  | ***β*** | ***SE*** | ***p*** | ***95% CI*** |  | ***β*** | ***SE*** | ***p*** | ***95%CI*** |  | ***β*** | ***SE*** | ***p*** | ***95%CI*** |  | ***β*** | ***SE*** | ***p*** | ***95%CI*** |  | ***β*** | ***SE*** | ***p*** | ***95%CI*** |  | ***β*** | ***SE*** | ***p*** | ***95%CI*** |
| **Between-samples parameters** |  |  |  |  |  |  |  |  |  |  |  |  |  |  |  |  |  |  |  |  |  |  |  |  |  |  |  |  |  |
| Mediterranean contrast |  |  |  |  |  |  |  |  |  |  |  |  |  |  |  |  |  |  |  |  |  |  |  |  |  |  |  |  |  |
| Region 🡪 Honor (path a) | .687 | .081 | < .001 | .529, .845 |  | .687 | .081 | < .001 | .529, .845 |  | .687 | .081 | < .001 | .529, .845 |  | .687 | .081 | < .001 | .529, .845 |  | .690 | .081 | < .001 | .531, .850 |  | .687 | .081 | < .001 | .529, .845 |
| Honor 🡪 Outcome (path b) | -.377 | .229 | .100 | -.827, .073 |  | -.810 | .183 | < .001 | -1.169, -.452 |  | -.178 | .319 | .577 | -.803, .447 |  | .339 | .140 | .015 | .065, .614 |  | .242 | .189 | .199 | -.127, .612 |  | -.440 | .346 | .204 | -1.119, .239 |
| Region 🡪 Outcome (path c') | .758 | .186 | < .001 | .394, 1.121 |  | -.094 | .230 | .684 | -.545, .358 |  | .427 | .430 | .320 | -.415, 1.269 |  | .241 | .144 | .093 | -.041, .522 |  | -.791 | .169 | < .001 | -1.122, -.460 |  | .658 | .159 | < .001 | .347, .969 |
| Total effect (path c) | .499 | .136 | < .001 | .233, .765 |  | -.650 | .204 | .001 | -1.049, .251 |  | .305 | .346 | .378 | -.374, .984 |  | .474 | .120 | < .001 | .240, .708 |  | -.624 | .136 | < .001 | -.890, -.358 |  | .356 | .196 | .070 | -.029, .740 |
| Indirect effect (a * b) | -.259 | .167 | .121 | -.586, .069 |  | -.557 | .143 | < .001* | -.838, -.275 |  | -.122 | .220 | .580 | -.554, .310 |  | .233 | .105 | .027† | .027, .439 |  | .167 | .135 | .215 | -.097, .432 |  | -.302 | .240 | .209 | -.774, .169 |
|  |  |  |  |  |  |  |  |  |  |  |  |  |  |  |  |  |  |  |  |  |  |  |  |  |  |  |  |  |  |
| West-East contrast |  |  |  |  |  |  |  |  |  |  |  |  |  |  |  |  |  |  |  |  |  |  |  |  |  |  |  |  |  |
| Region 🡪 Honor (path a2) | .057 | .076 | .448 | -.091, .206 |  | .057 | .076 | .448 | -.091, .206 |  | .057 | .076 | .448 | -.483, .093 |  | .057 | .076 | .448 | -.091, .206 |  | .070 | .091 | .445 | -.109, .249 |  | .057 | .076 | .448 | -.091, .206 |
| Honor 🡪 Outcome (path b) | -.377 | .229 | .100 | -.827, .073 |  | -.810 | .183 | < .001 | -1.169, -.452 |  | -.178 | .319 | .577 | -.803, .447 |  | .339 | .140 | .015 | .065, .614 |  | .242 | .189 | .199 | -.127, .612 |  | -.440 | .346 | .204 | -1.119, .239 |
| Region 🡪 Outcome (path c2') | .483 | .139 | .001 | .210, .756 |  | -.409 | .178 | .022 | -.758, -.060 |  | -.304 | .292 | .297 | -.875, .267 |  | .663 | .126 | < .001 | .416, .909 |  | -.474 | .112 | < .001 | -.693, -.256 |  | -.119 | .078 | .127 | -.272, .034 |
| Total effect (path c2) | .461 | .140 | .001 | .186, .737 |  | -.456 | .148 | .002 | -.747, -.165 |  | -.314 | .276 | .256 | -.856, .227 |  | .682 | .126 | < .001 | .436, .928 |  | -.457 | .104 | < .001 | -.661, -.253 |  | -.144 | .060 | .016 | -.262, -.027 |
| Indirect effect (a2 * b) | -.022 | .033 | .517 | -.087, .044 |  | -.047 | .062 | .454 | -.168, .075 |  | -.010 | .022 | .637 | -.053, .032 |  | .019 | .027 | .472 | -.034, .072 |  | .017 | .025 | .499 | -.032, .066 |  | -.025 | .038 | .506 | -.100, .049 |
|  |  |  |  |  |  |  |  |  |  |  |  |  |  |  |  |  |  |  |  |  |  |  |  |  |  |  |  |  |  |
| Modelled variance |  |  |  |  |  |  |  |  |  |  |  |  |  |  |  |  |  |  |  |  |  |  |  |  |  |  |  |  |  |
| *R^2^* (Honor) |  |  | 47.5% |  |  |  |  | 47.5% |  |  |  |  | 47.5% |  |  |  |  | 47.5% |  |  |  |  | 48.1% |  |  |  |  | 47.5% |  |
| *R^2^* (Outcome) |  |  | 53.6% |  |  |  |  | 97.5% |  |  |  |  | 20.8% |  |  |  |  | 75.1% |  |  |  |  | 62.9% |  |  |  |  | 24.9% |  |
|  |  |  |  |  |  |  |  |  |  |  |  |  |  |  |  |  |  |  |  |  |  |  |  |  |  |  |  |  |  |
| **Within-samples parameters** |  |  |  |  |  |  |  |  |  |  |  |  |  |  |  |  |  |  |  |  |  |  |  |  |  |  |  |  |  |
| Defense of Family Reputation   🡪 Outcome | .014 | .016 | .381 | -.017, .045 |  | .007 | .018 | .716 | -.029, .043 |  | -.042 | .012 | .001 | -.066, -.018 |  | .022 | .018 | .230 | -.014, .058 |  | -.014 | .018 | .158 | -.049, .022 |  | -.019 | .023 | .406 | -.064, .026 |
| Self-Promotion & Retaliation   🡪 Outcome | -.086 | .023 | < .001 | -.132, -.041 |  | -.001 | .020 | .973 | -.040, .039 |  | .035 | .022 | .105 | -.007, .077 |  | .037 | .018 | .039 | .002, .072 |  | .010 | .019 | .609 | -.028, .047 |  | .007 | .021 | .720 | -.033, .048 |
| Age 🡪 Outcome | -.060 | .021 | .004 | -.101, -.020 |  | -.016 | .017 | .349 | -.049, .017 |  | .025 | .015 | .106 | -.005, .054 |  | .019 | .016 | .297 | .013, .052 |  | .016 | .015 | .299 | -.014, .046 |  | .010 | .018 | .581 | -.044, .025 |
| SES 🡪 Outcome | .074 | .024 | .002 | .027, .121 |  | -.002 | .019 | .926 | -.038, .035 |  | .014 | .016 | .386 | -.017, .045 |  | .001 | .020 | .950 | -.037, .040 |  | -.010 | .014 | .461 | -.038, .017 |  | -.029 | .018 | .102 | -.063, .006 |
|  |  |  |  |  |  |  |  |  |  |  |  |  |  |  |  |  |  |  |  |  |  |  |  |  |  |  |  |  |  |
| Modelled variance |  |  |  |  |  |  |  |  |  |  |  |  |  |  |  |  |  |  |  |  |  |  |  |  |  |  |  |  |  |
| *R^2^* (Outcome) |  |  | 1.7% |  |  |  |  | < 0.1% |  |  |  |  | 0.3% |  |  |  |  | 0.3% |  |  |  |  | 0.1% |  |  |  |  | 0.1% |  |

*Note*. Presented are the standardized estimates for models testing the role of culture-level perceived normative honor values as a mediator of regional differences in exploratory outcomes. Models control for within-culture differences in two dimensions of perceived honor values, age, and SES. Cultural regions were coded using orthogonal contrasts for West-East category membership (0.5 = Anglo-West, -0.5 = East-Asia, 0 = Mediterranean) and for Mediterranean category membership (-0.5 = Anglo-West, -0.5 = East-Asia, 0.5 = Mediterranean). Indirect effects marked with a * meet a Holm-Bonferroni sequentially adjusted alpha level starting at .05 / 12 = .0042, to adjust for familywise error in combination with the corresponding tests involving personally endorsed honor values in Table S29.

## Table S29

#### Further Exploratory Mediation Results for Cultural Differences in Social Cognitive Tendencies via Personal Honor Values (Study 3)

| Model Parameters | **Outcome** | | | | | | | | | | | | | | | | | | | | | | | | | | | | |
| --- | --- | --- | --- | --- | --- | --- | --- | --- | --- | --- | --- | --- | --- | --- | --- | --- | --- | --- | --- | --- | --- | --- | --- | --- | --- | --- | --- | --- | --- |
|  | **Ingroup  Closeness Bias** | | | |  | **Nepotism (Reward Situations)** | | | |  | **Nepotism  (Punishment Situations)** | | | |  | **Dispositional  Attribution Bias** | | | |  | **Inclusion of  Contextual Information** | | | |  | **Thematic  Categorization** | | | |
|  | ***β*** | ***SE*** | ***p*** | ***95% CI*** |  | ***β*** | ***SE*** | ***p*** | ***95%CI*** |  | ***β*** | ***SE*** | ***p*** | ***95%CI*** |  | ***β*** | ***SE*** | ***p*** | ***95%CI*** |  | ***β*** | ***SE*** | ***p*** | ***95%CI*** |  | ***β*** | ***SE*** | ***p*** | ***95%CI*** |
| **Between-samples parameters** |  |  |  |  |  |  |  |  |  |  |  |  |  |  |  |  |  |  |  |  |  |  |  |  |  |  |  |  |  |
| Mediterranean contrast |  |  |  |  |  |  |  |  |  |  |  |  |  |  |  |  |  |  |  |  |  |  |  |  |  |  |  |  |  |
| Region 🡪 Honor (path a) | .125 | .160 | .436 | -.189, .438 |  | .125 | .160 | .436 | -.189, .438 |  | .125 | .160 | .436 | -.189, .438 |  | .125 | .160 | .436 | -.189, .438 |  | -.070 | .190 | .713 | -.443, .303 |  | .125 | .160 | .436 | -.189, .438 |
| Honor 🡪 Outcome (path b) | -.181 | .171 | .290 | -.516, .154 |  | -.664 | .140 | < .001 | -.939, -.390 |  | -.381 | .219 | .082 | -.810, .048 |  | .164 | .113 | .147 | -.058, .385 |  | .359 | .174 | .039 | .017, .701 |  | -.440 | .263 | .094 | -.956, .075 |
| Region 🡪 Outcome (path c') | .524 | .129 | < .001 | .272, .776 |  | -.537 | .207 | .010 | -.944, -.131 |  | .344 | .329 | .296 | -.301, .989 |  | .450 | .117 | < .001 | .221, .680 |  | -.588 | .127 | < .001 | -.836, -.340 |  | .412 | .158 | .009 | .102, .723 |
| Total effect (path c) | .502 | .138 | < .001 | .232, .771 |  | -.620 | .228 | .006 | -1.066, -.174 |  | .296 | .336 | .379 | -.363, .956 |  | .471 | .120 | < .001 | .235, .707 |  | -.613 | .138 | < .001 | -.883, -.343 |  | .358 | .196 | .068 | -.026, .741 |
| Indirect effect (a * b) | -.023 | .041 | .586 | -.104, .059 |  | -.083 | .104 | .425 | -.286, .120 |  | -.047 | .075 | .529 | -.195, .100 |  | .020 | .031 | .507 | -.040, .081 |  | -.025 | .064 | .695 | -.151, .101 |  | -.055 | .090 | .543 | -.232, -.122 |
|  |  |  |  |  |  |  |  |  |  |  |  |  |  |  |  |  |  |  |  |  |  |  |  |  |  |  |  |  |  |
| West-East contrast |  |  |  |  |  |  |  |  |  |  |  |  |  |  |  |  |  |  |  |  |  |  |  |  |  |  |  |  |  |
| Region 🡪 Honor (path a2) | -.105 | .079 | .184 | -.261, .050 |  | -.105 | .079 | .184 | -.261, .050 |  | -.105 | .079 | .184 | -.261, .050 |  | -.105 | .079 | .184 | -.261, .050 |  | -.153 | .114 | .182 | -.377, .071 |  | -.105 | .079 | .184 | -.261, .050 |
| Honor 🡪 Outcome (path b) | -.181 | .171 | .290 | -.516, .154 |  | -.664 | .140 | < .001 | -.939, -.390 |  | -.381 | .219 | .082 | -.810, .048 |  | .164 | .113 | .147 | -.058, .385 |  | .359 | .174 | .039 | .017, .701 |  | -.440 | .263 | .094 | -.956, .075 |
| Region 🡪 Outcome (path c2') | .427 | .136 | .002 | .160, .693 |  | -.487 | .169 | .004 | -.818, -.156 |  | -.339 | .268 | .207 | -.864, .187 |  | .702 | .123 | < .001 | .460, .944 |  | -.393 | .100 | < .001 | -.590, -.196 |  | -.192 | .079 | .015 | -.347, -.037 |
| Total effect (path c2) | .446 | .140 | .001 | .171, .721 |  | -.417 | .145 | .004 | -.701, -.133 |  | -.298 | .275 | .278 | -.838, .241 |  | .685 | .126 | < .001 | .438, .931 |  | -.448 | .103 | < .001 | -.651, -.246 |  | -.145 | .061 | .018 | -.265, -.025 |
| Indirect effect (a2 * b) | .019 | .020 | .341 | -.020, .058 |  | .070 | .052 | .180 | -.032, .172 |  | .040 | .039 | .307 | -.037, .117 |  | -.017 | .017 | .302 | -.050, .015 |  | -.055 | .049 | .261 | -.151, .041 |  | .046 | .042 | .269 | -.036, .129 |
|  |  |  |  |  |  |  |  |  |  |  |  |  |  |  |  |  |  |  |  |  |  |  |  |  |  |  |  |  |  |
| Modelled variance |  |  |  |  |  |  |  |  |  |  |  |  |  |  |  |  |  |  |  |  |  |  |  |  |  |  |  |  |  |
| *R^2^* (Honor) |  |  | 2.7% |  |  |  |  | 2.7% |  |  |  |  | 2.7% |  |  |  |  | 2.7% |  |  |  |  | 2.8% |  |  |  |  | 2.7% |  |
| *R^2^* (Outcome) |  |  | 48.3% |  |  |  |  | 98.8% |  |  |  |  | 31.8% |  |  |  |  | 71.6% |  |  |  |  | 70.2% |  |  |  |  | 33.8% |  |
|  |  |  |  |  |  |  |  |  |  |  |  |  |  |  |  |  |  |  |  |  |  |  |  |  |  |  |  |  |  |
| **Within-samples parameters** |  |  |  |  |  |  |  |  |  |  |  |  |  |  |  |  |  |  |  |  |  |  |  |  |  |  |  |  |  |
| Defense of Family Reputation   🡪 Outcome | .197 | .021 | < .001 | .156, .237 |  | .039 | .018 | .026 | .005, .074 |  | .034 | .022 | .131 | -.010, .077 |  | .053 | .022 | .016 | .010, .096 |  | -.025 | .016 | .110 | -.056, .006 |  | .091 | .030 | .003 | .031, .150 |
| Self-Promotion & Retaliation   🡪 Outcome | -.092 | .026 | < .001 | -.144, -.041 |  | .041 | .018 | .025 | .005, .076 |  | -.002 | .019 | .913 | -.040, .035 |  | -.094 | .019 | < .001 | -.130, -.057 |  | .050 | .019 | .007 | .014, .087 |  | .000 | .022 | .994 | -.044, .043 |
| Age 🡪 Outcome | -.063 | .020 | .002 | -.102, -.024 |  | -.014 | .019 | .466 | -.050, .023 |  | .021 | .014 | .124 | -.006, .048 |  | .021 | .017 | .218 | -.013, .055 |  | .017 | .015 | .265 | -.013, .047 |  | -.015 | .017 | .380 | -.048, .018 |
| SES 🡪 Outcome | .057 | .024 | .020 | .009, .104 |  | -.007 | .018 | .718 | -.043, .029 |  | .007 | .015 | .650 | -.023, .037 |  | -.002 | .020 | .914 | -.041, .037 |  | -.010 | .014 | .479 | -.038, .018 |  | -.042 | .017 | .016 | -.077, -.008 |
|  |  |  |  |  |  |  |  |  |  |  |  |  |  |  |  |  |  |  |  |  |  |  |  |  |  |  |  |  |  |
| Modelled variance |  |  |  |  |  |  |  |  |  |  |  |  |  |  |  |  |  |  |  |  |  |  |  |  |  |  |  |  |  |
| *R^2^* (Outcome) |  |  | 4.1% |  |  |  |  | 0.5% |  |  |  |  | 0.2% |  |  |  |  | 0.8% |  |  |  |  | 0.3% |  |  |  |  | 0.9% |  |

*Note*. Presented are the standardized estimates for models testing the role of culture-level perceived normative honor values as a mediator of regional differences in exploratory outcomes. Models control for within-culture differences in two dimensions of perceived honor values, age, and SES. Cultural regions were coded using orthogonal contrasts for West-East category membership (0.5 = Anglo-West, -0.5 = East-Asia, 0 = Mediterranean) and for Mediterranean category membership (-0.5 = Anglo-West, -0.5 = East-Asia, 0.5 = Mediterranean). Indirect effects marked with a * meet a Holm-Bonferroni sequentially adjusted alpha level starting at .05 / 12 = .0042, to adjust for familywise error in combination with the corresponding tests involving personally endorsed honor values in Table S28.

1. Modification indices overestimated the size of this cross-loading. When added into the model, it was estimated at 0.315, whereas the primary loading remained substantially higher at 0.469. [↑](#footnote-ref-2)
2. Owing to space constraints, we did not include measures of face and dignity in Study 2; focusing on honor reflected our goal of situating Mediterranean (“honor”) contexts in the global landscape. We chose honor values over honor concerns as these items showed the clearest geographical pattern in Study 1 and were substantially shorter in length. [↑](#footnote-ref-3)
